# Tunneling nanotubes regulate mitochondrial homeostasis between glioblastoma and astrocytes, and between tumor cells in vivo

**DOI:** 10.1038/s41467-026-76619-9

**Published:** 2026-09-15

**Authors:** Ines Saenz-de-Santa-Maria, Anna Pepe, Jean-Yves Tinevez, Christel Brou, Yakov Vitrenko, Thomas Cokelaer, Elizabeth Cohen-Jonathan Moyal, Roberto Weigert, Chiara Zurzolo

**Affiliations:** 1 Membrane Traffic and Pathogenesis Unit, Institut Pasteur, Université Paris Cité, CNRS UMR 3691, Paris, France; 2 Centro de Investigación Biomédica en Red de Cáncer (CIBERONC), Instituto de Salud Carlos III (ISCIII), Madrid, Spain; 3 Polymer Therapeutics Lab, Prince Felipe Research Center (CIPF), Valencia, Spain; 4 Institut Pasteur, Université Paris Cité, Image Analysis Hub, Paris, France; 5 Institut Pasteur, Université Paris Cité, Plate-forme Technologique Biomics, Paris, France; 6 Institut Pasteur, Université Paris Cité, Bioinformatics and Biostatistics Hub, Paris, France; 7 Université de Toulouse, Cancer Research Center of Toulouse (CRCT), Institut National de la Santé et de la Recherche Médicale (INSERM), Oncopole Claudius Regaud, Toulouse, France; 8 National Institutes of Health, Center for Cancer Research, National Cancer Institute, Laboratory of Cellular and Molecular Biology, Bethesda, MD USA

**Keywords:** Energy metabolism, Cancer stem cells

## Abstract

Tumor progression is driven by cancer cells’ ability to establish a cellular network through tunneling nanotube-like connections (TNTs), which enable mitochondrial exchange both within the tumor cells and with the tumor microenvironment (TME). However, the functional consequences of mitochondrial transfer between tumor and non-tumor cells, and its occurrence in vivo, remain poorly understood. Here we show bidirectional mitochondrial transfer between Glioblastoma (GBM) cells and non-tumoral astrocytes (AS). We report that transfer of damaged mitochondria from GBM cells to AS is associated with activation of mitophagy in recipient cells, while astrocyte-derived mitochondria to GBM cells correlates with changes in mitochondrial activity and metabolic readouts. Furthermore, intravital subcellular microscopy (ISMic) in a live animal model allows the visualization of TNT connections with characteristics similar to those observed in vitro and supported TNT-mediated mitochondrial transfer in vivo. These findings reveal a potential mechanism of tumor adaptation and highlight TNTs as promising therapeutic targets.

## Abstract (web summary)

Tunneling nanotube-like (TNT) connections allow mitochondrial exchange between tumor cells and its tumor microenvironment. Here, the authors show that TNT connections mediate bidirectional mitochondrial transfer between glioblastoma tumor cells and astrocytes in vitro, and between head and neck squamous cell carcinoma cells in vivo.

## Introduction

Mitochondria are double membrane bound organelles crucial for cell life and death. In addition to producing ATP via oxidative phosphorylation (OXPHOs), they also regulate programmed cell death and are the main source of reactive oxygen species (ROS)^1^. Mitochondrial exchanges either among tumor cells or among tumor and non-tumoral cells in the tumor microenvironment (TME), have been implicated in diverse processes, including metabolic reprogramming, immune suppression, cancer progression, modulation of therapeutic responses, activation of cellular rescue mechanisms, and evasion of apoptosis in recipient cells^2–15^.

A primary mechanism for mitochondria horizontal transfer occurs via transient intercellular structures known as tunneling nanotubes (TNTs)^16^. TNTs, described in vitro in a wide variety of tumors including Glioblastoma (GBM)^17,18^ and head and neck squamous cell carcinomas (HNSCC)^19^, are heterogeneous, highly dynamic membranous bridges that facilitate direct cytoplasmic exchanges between connected cells. They are classified into two major types: (1) Actin-based TNTs, which are short (up to 100 µm), thin ( ≤ 0.7 μm), and dynamic^20^; and (2) Microtubule (MT)-based TNTs, which are longer (up to 250 µm), thicker ( ≥ 0.7 μm), and more stable^12,21^. The latter increase in cancer cells when subjected to stress and have been proposed to favor survival of cells damaged by ischemia^22^, as well as following chemo/radiotherapy^12^_._ Indeed, the transfer of mitochondria between tumor cells and/or between tumor cells and non-tumoral cells by TNTs has an impact on tumor progression and resistance to chemotherapy^4,6–8,10,17,23,24^. Yet, how the composition of the TNTs influences the mitochondrial exchanges between cancer cells is unclear.

GBM is the most aggressive primary brain tumor in adults with no cure^25^. In GBM, tumor aggressiveness and progression are associated with a network of two types of intercellular connections: Tumor Microtubes (TMs) and TNTs^3,17,26,27^. A key feature of GBM is its complex TME, which includes non-tumoral cells such as astrocytes (AS)^28^. Notably, mitochondrial transfer from AS to GBM stem cells (GSCs) via TMs, enhances tumorigenesis through a mechanism depending on GAP-43 (growth associated protein enriched in growth cones)^3^. Interestingly, TNTs have been shown to coexist with TMs in 3D GBM organoids formed by GSCs, and are hypothesized to cooperate with TMs in promoting tumor radio-resistance^17^. Consistently, TNT mediated mitochondria transfer from mesenchymal stem cells (MSC) to GSCs has been shown to induce GSC metabolic reprogramming and resistance to temozolomide (TMZ)^10^. Conversely, TNTs have also been demonstrated to mediate mitochondrial transfer from GBM cell lines to immortalized non-tumoral AS, facilitating their metabolic adaptation and hypoxia response within the TME^24^. Furthermore, AS have been shown to play a neuroprotective role by degrading damaged mitochondria^29^ and transferring healthy ones to neurons via extracellular vesicles (EV)^30^.

Based on these findings, we hypothesize that GBM cells exploit TNTs for the bidirectional transfer of both healthy and damaged mitochondria with non-malignant cells in the TME, particularly AS, as a mechanism to enhance cancer cell survival.

To date, the existence of TNTs in cancer in vivo has been questioned due to technological limitations that made their detection arduous in living animals. The optical resolution of classical microscopy does not allow for the morphological characterization of these thin connections in a tissue environment, and there is currently no TNT-specific marker available^21^. Only TM, thicker neurite-like connections, have been visualized in cancer^26^ in vivo. These structures have recently been proposed to mediate mitochondria transfer, by confocal imaging of fixed tissue samples^3^. Consequently, whether functional TNTs facilitate mitochondrial transfer in tumors in living animals remains an open and challenging question.

Here we investigated whether different types of GBM cells communicate with each other and with AS through TNTs, focusing on the horizontal transfer of mitochondria. We used various cell models, including U251 GBM cell line, patient derived GSCs: GSC (-) and GSC (*) (also known as SRC1 and SRC2), with different metabolic activities and responsible of tumor relapse^17,31^, as well as primary murine AS, to investigate bidirectional mitochondrial transfer. Our findings revealed that damaged mitochondria from GBM cells undergo mitophagy after transfer in AS. Conversely, RNA sequencing of GSCs receiving AS-derived healthy mitochondria demonstrated upregulation of mitochondrial metabolic pathways, supporting a role for AS derived-mitochondria in enhancing the metabolic functions of GBM cells.

To investigate whether mitochondrial transfer via TNTs occurs in cancer in vivo, we employed Intravital Subcellular Microscopy (ISMic) a cutting-edge technique that enables real-time visualization of biological processes in live animal^32–34^. Using this approach, we observed TNTs exhibiting characteristics similar to those identified in vitro, containing mitochondria within the structures. Furthermore, we detect exogenous mitochondria in the receiving cell population, providing direct evidence of mitochondrial transfer in vivo.

Our multidisciplinary approach, which combines in vitro and in vivo systems, provides significant insights into TNT-mediated interactions within TME and their role in cancer resilience, underscoring the potential of targeting this process in future therapeutic strategies.

## Results

### Dynamic analysis of TNT-mediated mitochondrial transfer between live cancer cells

**Fig. 1:**
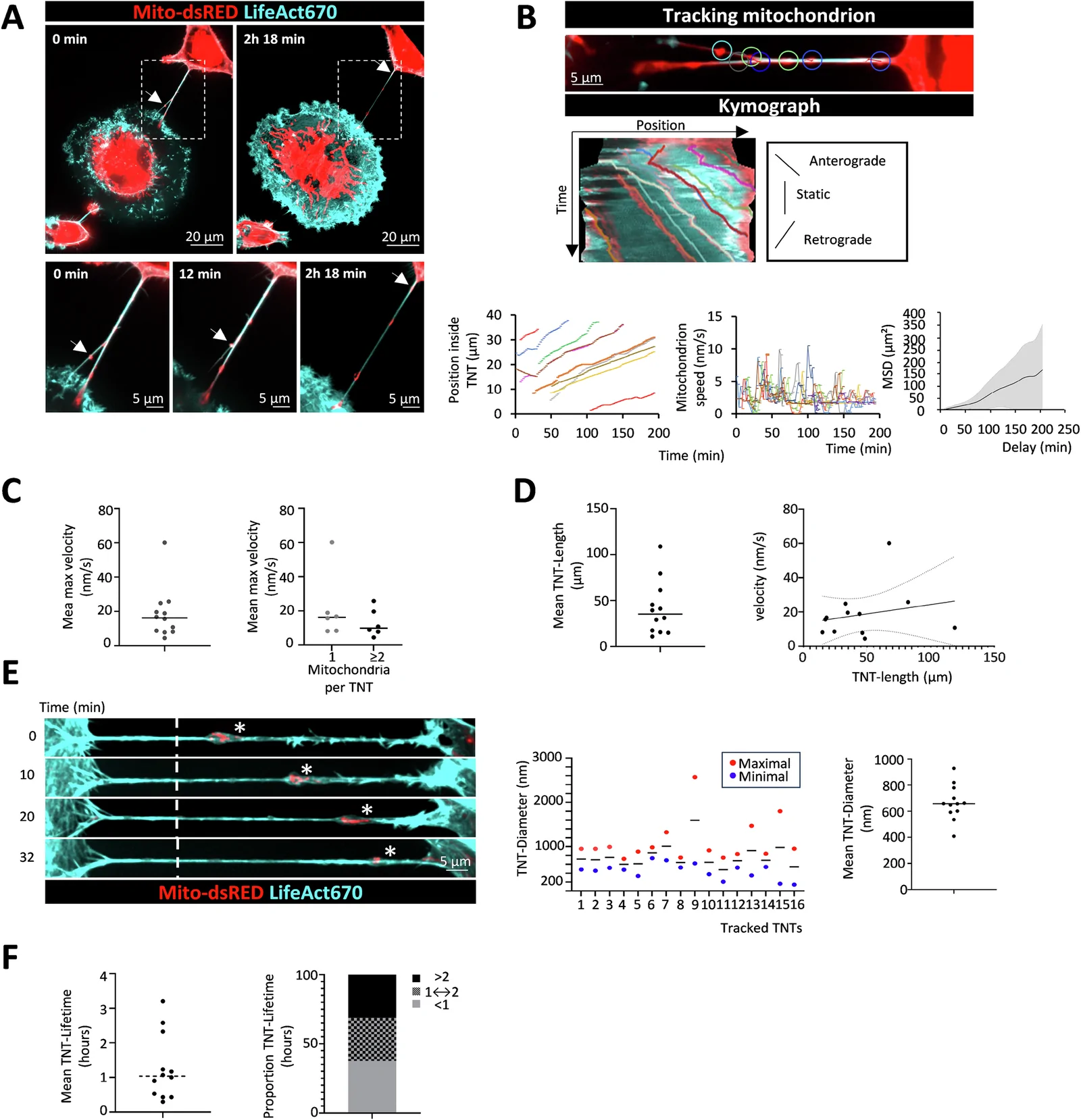
In-depth analysis of mitochondrial transfer through TNTs in U251 cells. **A** Time-lapse imaging of mitochondrial transfer through TNTs in U251 cells co-transduced with pLV-lifeAct-670 (actin, light blue) and pLV-mitoDsRED (mitochondria, red). Insets show mitochondrial movement within a branched TNT (Supplementary Movie 1). White arrows indicate migrating mitochondria. Six independent experiments. Scale bars, 20 µm (top) and 5 µm (bottom). **B** Mitochondrial tracking and representative kymographs generated using the TrackMate plugin show anterograde, retrograde and static trajectories. Kymograph-based quantification of distance travelled (left) and mitochondrial speed (middle) is shown. Mean squared displacement (MSD; right) was calculated from 18 mitochondrial tracks across nine independent biological experiments. An MSD slope >1 indicates active transport (one-sided *t*-test, *P* = 1.1 × 10⁻¹⁶). **C** Left, mean maximum mitochondrial velocity per independent experiment (*n* = 12; 56 mitochondria from 16 TNTs). Horizontal line, median (16.2 nm/s); the highest mean maximum mitochondrial velocity was 60.2 nm/s. Right, comparison of mean maximum mitochondrial velocity between TNTs containing one mitochondrion (7 tracked) or ≥2 mitochondria (49 tracked) (*n* = 6 per group). Horizontal line, mean. Mann–Whitney *U* test, ns. **D** Left, mean TNT length per independent experiment (*n* = 12; 16 TNTs; 530 time points). Horizontal line, median (35.31 µm). Right, correlation between mean maximum TNT length and mean maximum mitochondrial velocity (linear regression, *R*² = 0.05049). **E** TNT apparent diameter measured over time using the LifeAct intensity profile, excluding membrane bulging (Supplementary Movie 2). Representative measurements are shown on the left; dashed lines indicate measurement sites and asterisks indicate membrane bulging containing mitochondria. Scale bar, 5 µm. Centre, maximal (red) and minimal (blue) apparent diameters for each of 16 TNTs. Right, mean apparent TNT diameter per independent experiment (*n* = 12; 16 TNTs; 464 time points). Horizontal line, median (657.9 nm; range, 446.2–928.6 nm). **F** TNT persistence analysis. Left, mean TNT lifetime per independent biological experiment (*n* = 12; 16 TNTs). Horizontal line, median (1.03 h). Right, distribution of TNT lifetime categories ( < 1 h, 1–2 h and >2 h). Exact *P* values and summary statistics are provided in the Source Data.

TNT-mediated intercellular communication between distant cells is well documented in several tumor cells in vitro, including GBM^17,19,35,36^. However, most of the features of the TNTs, including diameter, length, and cytoskeletal composition were determined in fixed samples. Since chemical fixation partially disrupts the thinner TNTs supported exclusively by actin filaments (here referred as Actin-based TNTs), we used time-lapse imaging to study: i) the dynamics of TNT-mediated mitochondrial transfer between different cells, and ii) the TNT cytoskeletal composition and its role in TNT-mediated cell-to-cell communication. To this end, we used U251, the most widely used GBM-derived cell line, and GSCs derived from infiltrative regions of the same GBM patient, which exhibit different metabolic activities (GSC - and GSC +) and have been linked to relapse^31^. First, we co-transfected U251 cells with an inner mitochondrial membrane marker fused to RFP (pLV-mitodsRED) and the F-actin sensor Life-Act fused to iRFP670 (pLV-lifeAct-670). We found that cells established TNT-like connections that allowed mitochondria to be transferred (Fig. 1A and Supplementary Movie 1) and observed that mitochondrial speeds vary throughout the transfer process, exhibiting a saltatory motion with frequent stopping phases (Fig. 1B left-middle). Mean-square displacement (MSD) measures of mitochondrial trajectories suggested that mitochondrial transfer is due to active transport through the connections (MSD slope curve > 1, Fig. 1B right)^37^.

Time-resolved tracking of individual mitochondria showed a median maximum mitochondrial velocity of 16.22 nm/s (56 mitochondria tracked within 16 TNTs, 12 independent experiments), consistent with previously reported mitochondrial migration velocities via TNTs^19,38^ (Fig. 1C left, and Supplementary Fig. 1). Individual mitochondria inside TNTs showed no differences average maximum velocity than mitochondria moving in groups (Graph 1 C, right).

Due to the movements of the connected cells the TNT length varied over time. Repeated measurements from 16 TNTs (530 time points) showed lengths ranging from 10.92 to 108.9 µm. The median mean TNT length across 12 independent biological experiments was 35.31 µm (Fig. 1D, left). Maximum mitochondrial velocity was independent of TNT length (Fig. 1D, right).

A key feature distinguishing TNTs from other types of connections, such as TMs, is their diameter. TNT diameters ranged from 20 to 700 nm (thin canonical TNTs) and up to 1 µm (thick TNTs)^20,21^. In several TNTs, we observed membrane dilations (previously termed gondolas^39^) filled with mitochondria moving along the TNT during mitochondrial transfer (Fig. 1E, Supplementary Movie 2). These gondolas temporarily increased the diameter of the TNT and facilitated the exchange of globular or tubular mitochondria, with diameters ranging from 0.5 to 1 µm.

We measured the apparent TNT diameter over time throughout the entire movies, excluding gondolas. All connections fell within the typical size range of TNTs, with a median apparent diameter of 657.9 nm (range, 446.2–928.6 nm; Fig. 1E), supporting the idea that local membrane remodeling (i.e., gondolas) facilitates the transfer of mitochondria through thin TNTs ( < 700 nm). All values were consistent with the diffraction-limited resolution of spinning-disk confocal microscopy.

Several studies have shown that TNTs can persist from several min to several h^19,21^, with their stability influenced by actin dynamics, presence of MT^38^ or other proteins such as EPS8, IRSp53^40^, and CD9^41^. The mean TNT lifetime was 1.03 ± 1 h, with the longest recorded lifetime reaching up to 7 h in rare cases (Fig. 1F, Supplementary Movie 3). Moreover, we confirmed that TNTs formed between two distinct cells and not between daughter cells post-division, as shown by co-culturing cells expressing lifeAct fused with different fluorophores, 670 and BFP2 (Supplementary Fig. 1 and Movies 3–4).

### TNT-transferred mitochondria integrate into the recipient cell’s mitochondrial network

**Fig. 2:**
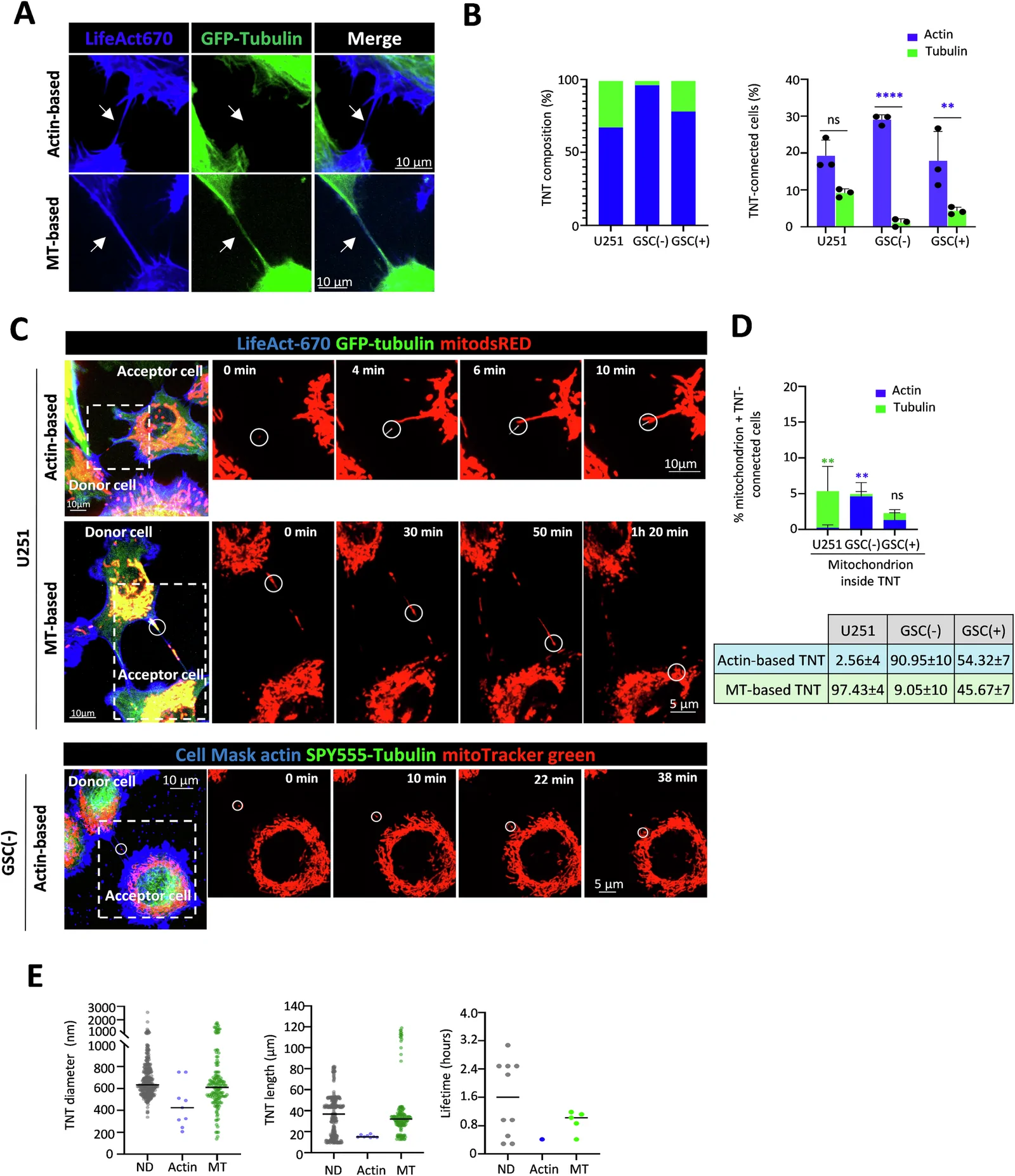
Mitochondria transferred via TNTs fuse with the recipient mitochondrial network regardless of TNT cytoskeletal composition. **A** U251 cells co-transduced with pLV-LifeAct670 (actin, blue) and pLV-GFP-tubulin (microtubules, green), showing Actin-based TNTs (actin only) and MT-based TNTs (actin and microtubules). Arrowheads indicate TNTs. Scale bar, 10 µm. Representative image from three independent biological experiments (807 cells analyzed). **B** TNT composition in U251, GSC(−), and GSC(+) cells. Left, proportion of Actin-based (blue) and MT-based (green) TNTs; subtype distribution differed among cell types (χ² test). Right, percentage of TNT-connected cells by subtype. GSC(−) and GSC(+) cells showed more Actin-based than MT-based TNTs, whereas no significant difference was observed in U251 cells (two-way ANOVA). Total cells analyzed: U251, *n* = 807; GSC(−), *n* = 1313; GSC(+), *n* = 1295; *N* = 3 independent biological experiments. **C** Mitochondrial transfer through Actin- and MT-based TNTs followed by fusion with the recipient mitochondrial network (Supplementary Movies 10–12). Left, time 0; right, transferred mitochondrion (white circle) immediately before fusion. Top, representative Actin-based transfer in U251 (one complete event in one of six live-cell imaging experiments). Middle, representative MT-based transfer in U251 (five complete events across six experiments). Bottom, representative Actin-based transfer in GSCs (one complete event in one of three experiments). **D** Percentage of mito⁺ TNT-connected cells. Blue and green bars indicate Actin- and MT-based TNTs, respectively. A significant interaction between cell type and TNT subtype was detected (two-way ANOVA). Significant differences between subtypes were observed in U251 and GSC(−), but not GSC(+) cells. Cell numbers are as in (**B**). **E** Apparent TNT diameter, length, and lifetime of ND-, Actin-, and MT-based TNTs. Each dot represents one measurement at an individual time point. Multiple measurements from the same TNT illustrate temporal variability and were excluded from statistical inference. Measurements from the single Actin-based TNT, corresponding to one mitochondrial transfer event, are shown for descriptive purposes only and were excluded from statistical analyses. Summary statistics are provided in the Source Data.

Next, we sought to determine whether the cytoskeletal composition of TNTs in GB/GSC affects mitochondrial transfer. To simultaneously label mitochondria, actin filaments, and MT, we co-transduced U251, GSC (-), and GSC (+) cells with three different plasmids: pLV-mito-dsRED, pLV-lifeAct fused to either BFP2 (blue) or iRFP670 (far-red), and pLV-GFP-tubulin (Fig. 2 A). The expression of these probes did not disrupt the normal mitotic behavior of the cells (Supplementary Fig. 2 and Movie 5). The nature of TNTs appears to be cell specific. One-third of the TNTs in U251 cells (32.76 ± 7%) contained both F-actin and MT (MT-based TNTs), while the remaining TNTs contained only F-actin (Actin-based TNTs). In contrast, GSC (-) contained primarily Actin-based TNTs (96.20 ± 4%) whereas GSC (+) cells also displayed a predominance of Actin-based TNTs (78.48 ± 10% vs. 21.52 ± 10% MT-based) (Fig. 2B).

Both types of TNTs supported the transfer of mitochondria from donor (D) to recipient cells (Fig. 2C, Supplementary Fig. 3, and Supplementary Movies 6–7 and 8 for Actin- and MT-based TNTs, respectively), although the extent of the transfer was cell-type dependent. Specifically, in U251 cells we found that MT-based TNTs transported the vast majority of the mitochondria present in TNTs (97.43 ± 4%), despite the higher frequency of Actin-based TNTs cells (Fig. 2D). On the other hand, in GSC (-) mitochondria were transported almost exclusively by Actin-based TNTs (90.95 ± 10%), whereas in GSC (+) both kind of TNTs equally contributed to the mitochondrial transport (45.67 ± 7% MT-based, 54.32 ± 7% Actin-based). Notably, these results show that the transport of mitochondria does not correlate with the number of a particular kind of TNTs.

We also analyzed the apparent diameter, length, and lifetime of the different mitochondria-transferring TNTs. Among six independent live-cell imaging experiments, mitochondrial transfer through an Actin-based TNT was observed only once, allowing measurements of TNT length and apparent diameter over time. Throughout the imaging period, this Actin-based TNT exhibited a smaller apparent diameter and shorter length than MT-based and non-determined (ND) TNTs (Fig. 2E, left). The mean apparent diameter of the Actin-based TNT was 446 ± 201 nm, compared with 669 ± 297 nm for MT-based TNTs and 684 ± 191 nm for ND-TNTs. The mean TNT length was 15.69 ± 1.27 µm, compared with 37.24 ± 25.01 µm for MT-based TNTs and 35.31 ± 19.18 µm for ND-TNTs (Fig. 2E). Because these measurements derive from a single mitochondrial transfer event, they are presented descriptively and were not subjected to statistical analysis. No significant differences in TNT diameter, length, or lifetime were detected between MT- and ND-based TNTs (Supplementary Fig. 3B), consistent with the predominant role of MT-based TNTs in mitochondrial transfer shown in Fig. 2D.

To overcome the diffraction limit of spinning-disk microscopy and accurately quantify TNT diameter, we examined TNT ultrastructure by cryo-electron microscopy (cryo-EM). Consistent with our previous work in SH-SY5Y cells^20^, TNTs in U251 cells that appeared as single tubes were instead composed of multiple individual TNTs (iTNTs), with diameters of 60–120 nm, containing actin alone (Actin-TNTs) or actin together with microtubules (MT-TNTs). We also observed bundles of iTNTs with diameters up to **~**1 µm, which may explain the thickest TNTs observed by spinning-disk microscopy (Supplementary Fig. 4 and Supplementary Movie 9).

Finally, the Actin-based TNT remained stable for 26 min (Fig. 2E, right) whereas the median lifetime of ND- and MT-based TNTs was 1.67 h and 1.07 h, respectively (Supplementary Fig. 3B), supporting the hypothesis that MTs increase the stability of TNTs^21^. Because this observation is based on a single Actin-based mitochondrial transfer event, it is presented descriptively. No significant difference in TNT lifetime was detected between ND- and MT-based TNTs (Supplementary Fig. 3B). Regardless of whether transfer occurred through Actin- or MT-based TNTs, transferred mitochondria successfully integrated into the mitochondrial network of the recipient cell, as demonstrated by pseudo-colored time-resolved imaging (Fig. 2C, Supplementary Fig. 3D, and Movies 10–12).

### Mitochondria transfer bidirectionally between tumor and healthy cells through TNTs

**Fig. 3:**
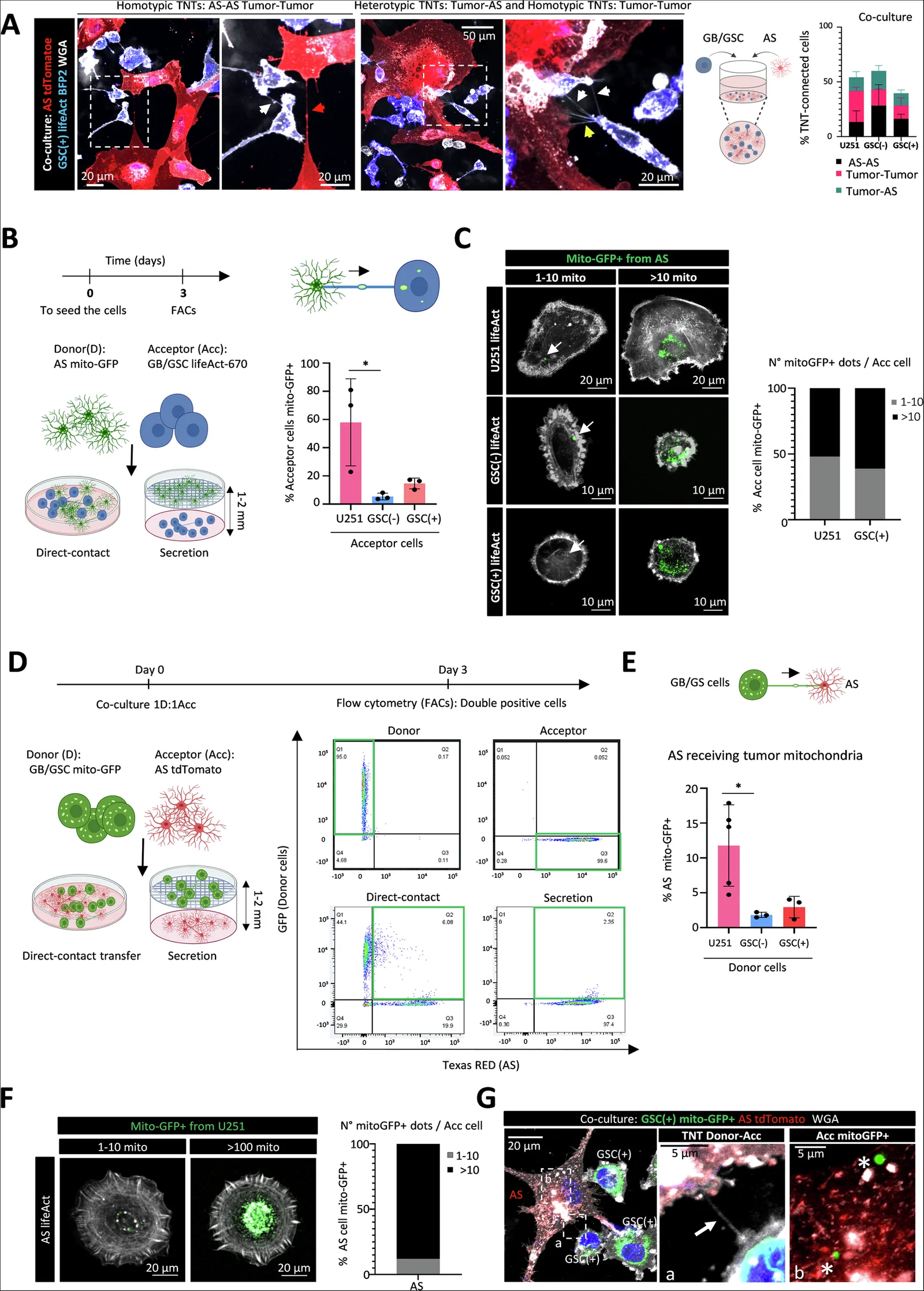
GB/GS cells form functional heterotypic TNTs with primary cortical AS, enabling mitochondrial transfer. **A** GSC(+) cells expressing pLV-LifeAct-BFP2 (actin, blue) were co-cultured with primary cortical AS expressing tdTomato (red) and stained with WGA (white). Homotypic (Tumor–Tumor and AS–AS) and heterotypic (Tumor–AS) TNTs are indicated by white/red and yellow arrowheads, respectively. Scale bars: 20 µm (left), 50 µm (right), and 20 µm (insets). Representative of three independent biological experiments. Right, percentage of homotypic (AS–AS, black; Tumor–Tumor, pink) and heterotypic (Tumor–AS, green) TNT-connected cells. Total cells analyzed: U251–AS, *n* = 1,409; GSC( − )–AS, *n* = 1,223; GSC( + )–AS, *n* = 2,234; *N* = 3 (U251–AS and GSC( + )–AS) and *N* = 2 (GSC( − )–AS). Representative images of U251–AS and GSC( − )–AS co-cultures are shown in Supplementary Fig. 5D. **B** AS-to-tumor mitochondrial transfer. AS expressing mitoGFP were co-cultured with tumor cells either in direct contact or separated by a 1 µm pore filter (secretion control). After 3 days, flow cytometry quantified the percentage of tumor cells containing AS-derived mitochondria after subtraction of secretion-mediated uptake. At least 10,000 events were acquired by condition. Each independent experiment included 3 technical replicates for the direct-contact condition and 2 secretion-control conditions. Data are presented as the mean of 3 independent experiments (Kruskal–Wallis test with Dunn’s multiple-comparisons test; *N* = 3). **C** Sorted tumor cells containing AS-derived mitochondria. Representative cells containing 1–10 or >10 mitochondrial particles (left) and mitochondrial particle distribution in U251 and GSC(+) cells (right). Scale bars: 20 µm (U251) and 10 µm (GSC(+). Total cells analyzed: U251, *n* = 77; GSC(+), *n* = 98; *N* = 5 (U251) and *N* = 3 (GSC(+)). **D** Representative flow cytometry plots of direct-contact and secretion-control conditions. **E** Flow cytometry quantification of the percentage of AS cells containing tumor-derived mitochondria after subtraction of secretion-mediated uptake, as in experiment B (Kruskal–Wallis test with Dunn’s multiple-comparisons test; *N* = 5 for U251; *N* = 3 for GSC(−) and GSC(+)). **F** Representative image of sorted AS containing tumor-derived mitoGFP⁺ mitochondria. Distribution of AS by mitochondrial particle number. Scale bar, 20 µm. Total AS analyzed: *n* = 110; *N* = 3. **G** GSC(+) mitoGFP (green) co-cultured with tdTomato-expressing AS (red). Insets show a heterotypic TNT (arrow) and an AS containing tumor-derived mitochondria (white asterisks). Scale bars: 20 µm (left) and 5 µm (insets). Qualitative observation (*N* = 3). Exact *P* values and summary statistics are provided in the Source Data. Created in BioRender. Saenz-de-Santa-Maria, I. (2026) https://BioRender.com/1tdm77o.

To assess whether tumoral cells communicate via TNTs with non-malignant cells from the TME, we decided to focus on AS because they make up 40–50% of the glial cells in the brain and play a crucial role in supporting neurons^30^. Moreover, Brisudova and colleagues have recently identified AS and not microglia as the predominant mitochondrial donors in the brain^14^. We co-cultured non-tumoral primary murine AS (Supplementary Fig. 5A, B) with GB/GS cells. We observed that both homotypic (GB/GS-GB/GS or AS-AS) and heterotypic (GB/GS-AS) TNTs were established in 2D-culture and in tumor organoids (Fig. 3A, Supplementary Fig. 5 C-E). Moreover, individual tumor cells formed heterotypic and homotypic connections simultaneously (Fig. 3A). To determine the directionality of the transfer, we transfected one of the cell populations (termed D) with a GFP-tagged mitochondrial marker (mito-GFP).

Since mitochondria can be transferred by extracellular vesicles, we used a co-culture system where both populations were separated by a 1 µm diameter filter, but in contact with the same culture medium, as a secretion control. This filter prevents direct contact between D and acceptors (Acc) cells while allowing the passage of mitochondria (0.5–1 µm diameter).

The percentage of mitochondrial transfer in both conditions—secretion and normal co-culture—was assessed after 3 days using flow cytometry (FACS) by quantifying the double-positive cells. The final percentage of Acc cells receiving mitochondria by direct contact was calculated by subtracting the percentage of mitochondrial transfer via secretion from the overall percentage (Fig. 3B). However, only a minimal double-positive population was detected under secretion conditions, likely reflecting background or artefactual fluorescence rather than effective mitochondrial transfer. In contrast, direct co-culture revealed a clearly distinct double-positive population, supporting TNT-mediated transfer as the predominant mechanism. We have therefore excluded secretion as a major contributor.

We found that AS, when examined as D, transferred mitochondria to 50.72 ± 28% of the U251 cells, as well as 5.43 ± 2% and 17.99 ± 10% to GSC (-) and GSC (+), respectively (Fig. 3B, Supplementary Fig. 6A). The transfer was also confirmed by microscopy (Fig. 3C). Conversely, when AS were examined as Acc, the transfer from GB/GS cells was less efficient. Indeed, only 7.95 ± 3% of the AS cells received mitochondria from U251 cells, and in the case of GSC (-) and GSC ( + ), 1.60 ± 0.3 and 1.39 ± 1%, respectively (Fig. 3E). Also, in this case, we confirmed the transfer of tumor derived-mitochondria by confocal microscopy (Fig. 3F, G, Supplementary Fig. 6A).

These data show that mitochondria can be transferred bidirectionally between AS and tumor cells, with the transfer from AS to tumor cells being quantitatively predominant.

### AS degrade dysfunctional mitochondria transferred from tumor cells via TNTs

**Fig. 4:**
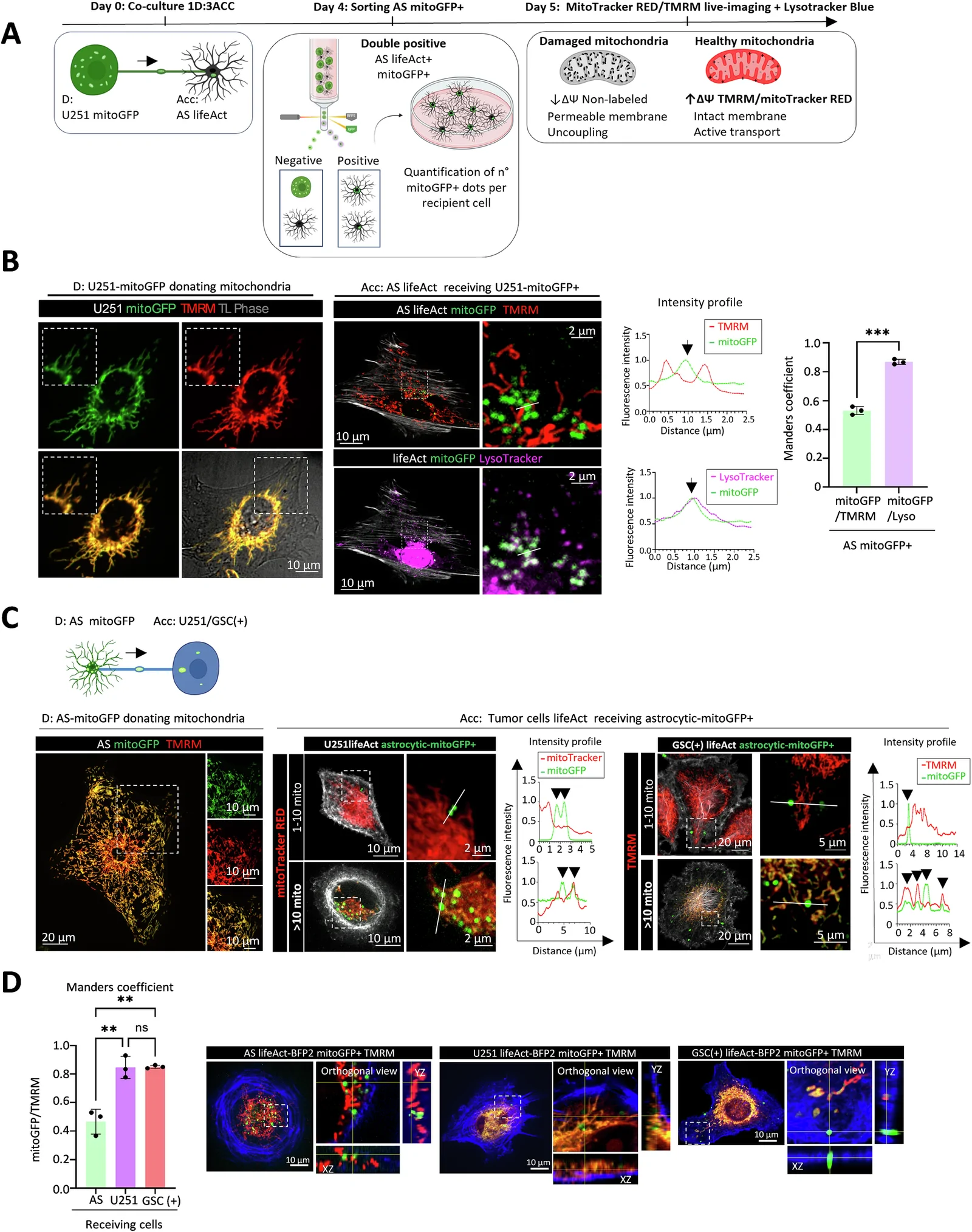
State and fate of mitochondria transferred to AS and GB/GS recipient cells. **A** Experimental design to assess the functional state and fate of mitochondria transferred from U251 cells to AS via TNTs. U251 mitoGFP cells were co-cultured with LifeAct-670-expressing AS (1:3). After 4 days, AS containing tumor-derived mitochondria (LifeAct⁺/mitoGFP⁺) were sorted, cultured overnight, and stained with TMRM or MitoTracker Red CMXRos (ΔΨ, red) together with LysoTracker Blue (lysosomes, magenta) for live-cell imaging. Mitochondria lacking ΔΨ were not labeled by TMRM. **B** Representative colocalization analysis of donor U251 mitoGFP cells (left) and recipient AS (right). AS-derived mitochondria colocalized with TMRM, whereas transferred tumor-derived mitochondria colocalized with LysoTracker but not TMRM, consistent with mitophagy. Intensity profiles are shown on the right; arrowheads indicate representative regions of signal overlap or separation. Manders’ colocalization coefficients showed greater colocalization of transferred tumor-derived mitochondria with LysoTracker than with TMRM. Scale bar, 10 µm. Total cells analyzed: *n* = 59; *N* = 3. Paired two-tailed *t*-test; *P* = 0.0003. **C** Representative images of donor AS mitoGFP cells (left) and recipient U251 and GSC(+) cells (right). Donor AS showed complete mitoGFP/TMRM colocalization. Recipient cells containing 1–10 or >10 are shown. Single transferred AS-derived mitochondria lacked ΔΨ, whereas cells containing >10 transferred AS-derived mitochondria showed progressive integration into the recipient mitochondrial network. Black arrows indicate mitoGFP intensity peaks. Scale bars: 10 and 2 µm (U251) and 20 and 5 µm (GSC(+)). Total cells analyzed: U251, *n* = 77, *N* = 5; GSC(+), *n* = 98, *N* = 3. **D** Mitochondrial activity in recipient AS, U251, and GSC(+) cells quantified by Manders’ colocalization coefficients (mitoGFP/TMRM). Recipient AS showed significantly lower colocalization than U251 and GSC(+) cells, whereas no difference was detected between U251 and GSC(+) cells. Two-way ANOVA with Tukey’s multiple-comparisons test. Total cells analyzed: AS, *n* = 62; U251, *n* = 41; GSC(+), *n* = 101; *N* = 3. Right, Z-projections and orthogonal views show inactive mitoGFP⁺ mitochondria in AS and active mitoGFP⁺/TMRM⁺ mitochondria integrated into the recipient mitochondrial network in U251 and GSC(+) cells. Exact *P* values and summary statistics are provided in the Source Data. Created in BioRender. Saenz-de-Santa-Maria, I. (2026) https://BioRender.com/60ntnq1.

We hypothesized that mitochondrial transfer could enable cancer cells to recruit functional mitochondria from AS and/or dispose of damaged ones. To test this hypothesis, we assessed the status of the transferred mitochondria and investigated their fate in the recipient cell. To monitor the accumulation of mitochondria over time, we extended the co-culture of mitoGFP-U251 and LifeAct670-AS to 4 days (See Fig. 4A). Then, we sorted the AS containing U251 derived-mitoGFP+ (Supplementary Fig. 6B) and quantified the number of mitochondria per Acc cell. The collected cells were plated for an additional 18 h, after which mitochondrial functionality was assessed by labeling them with mitoTracker RED CMXRos or tetramethylrhodamine methyl ester (TMRM), which are retained by energetically active mitochondria^42^.

Colocalization analysis (Mandersˈ overlap coefficient) showed that mitochondria transferred from the GB/GS cells (mitoGFP-labelled) to the AS did not fuse with the Acc mitochondrial network, did not label with TMRM (mitoGFP/TMRM coefficient = 0.48 ± 0.2) and were globular in shape (Fig. 4B). Moreover, they showed strong colocalization with the lysosomal marker LysoTracker Blue as shown by analysis of the Mender coefficient (mitoGFP/LysoTracker coefficient = 0.77 ± 0.2), and the fluorescence intensity profile (Fig. 4B). This indicates a significant association of transferred mitochondria with lysosomes suggesting they underwent mitophagy (Fig. 4B). As expected, the native mitochondria were labelled with TMRM in both D and Acc and exhibited a tubular shape (Fig. 4B). On the other hand, mitochondria transferred from the AS into the GB/GC cells were more heterogenous (Fig. 4C). In cells where few mitochondria were transferred ( < 10/cells), they did not fuse with the existing mitochondria network, whereas in cells receiving a larger number of mitochondria, the transferred organelles fused with the Acc mitochondrial network, colocalized with TMRM, and maintained a tubular morphology (Fig. 4C).

Furthermore, in homotypic U251–U251 transfer experiments, mitoGFP-positive mitochondria co-labeled with MitoTracker™ Red CMXRos were observed traveling through TNTs, indicating preservation of membrane potential during transfer (Supplementary Fig. 7 and Movie 13).

These findings suggested that TNTs serve potentially to maintain viable mitochondria homeostasis in cancer cells by facilitating both the disposal of damaged mitochondria from the cancer cells and the acquisition of functional mitochondria from the healthy cells. This conclusion is supported by Mandersˈ colocalization analysis across the three populations studied, AS, U251, and GSC (+) (Fig. 4D).

### The expression of mitochondrial respiration related genes is enhanced in GSC receiving astrocytic-derived mitochondria

**Fig. 5:**
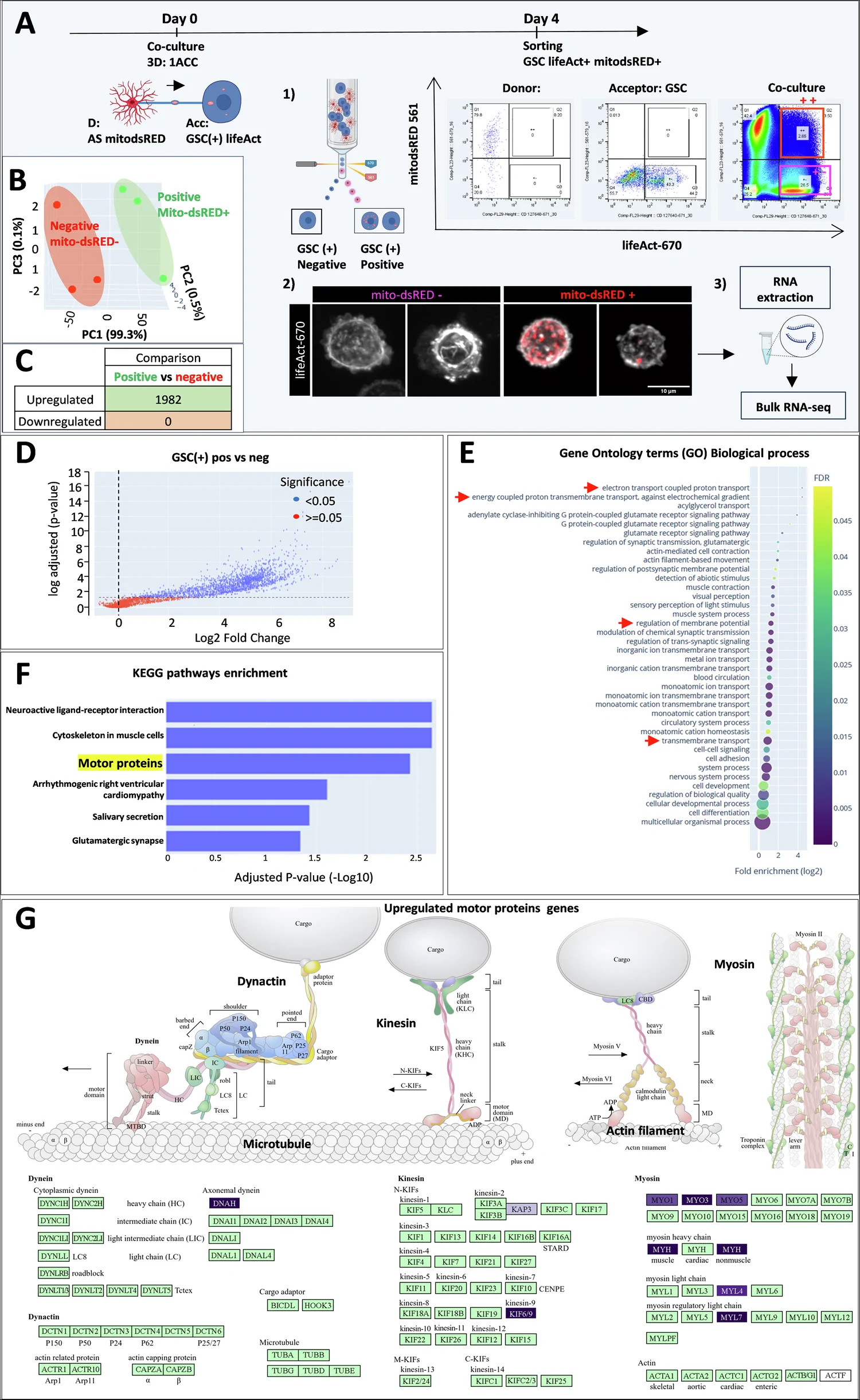
Bulk RNA-seq analysis of GSC(+) cells receiving astrocytic mitochondria. **A** Experimental design for co-culture, sorting of GSC(+) cells with (mitoDsRED + ) or without (mitoDsRED − ) astrocytic mitochondria, representative FACS plot and sorted cells. *N* = 3 independent biological co-culture experiments. **B**–**F** Differential gene expression analysis. **B** Three-dimensional principal component analysis (PCA) showing separation of GSC mitoDsRED⁻ (neg, red) and mitoDsRED⁺ (pos, green) samples. **C** Summary of differentially expressed genes. **D** Volcano plot comparing GSC mitoDsRED⁺ and mitoDsRED⁻ cells. Blue dots indicate significantly differentially expressed genes (adjusted *P* < 0.05); dashed lines indicate fold change >1.5. Differential expression was assessed using the DESeq2 Wald test with Benjamini–Hochberg correction. **E** Gene Ontology analysis of enriched biological processes in GSC mitoDsRED⁺ versus mitoDsRED⁻ cells. **F** KEGG pathway enrichment analysis showing six enriched pathways, including motor proteins. **G** Schematic representation of cytoskeletal motor proteins associated with microtubule- and actin-based transport (top) and expression of the corresponding motor protein genes (bottom). Dark blue indicates genes with log₂ fold change ≥4. Exact *P* values and summary statistics are provided in the Source Data, where applicable. Created in BioRender. Saenz-de-Santa-Maria, I. (2026) https://BioRender.com/wjzuwqe and with Damona project.

We hypothesized that TNTs help cancer cells to maintain or boost their metabolism by transferring whole healthy organelles from AS to tumor cells. To investigate this, we explored the transcriptional changes induced by TNT-dependent transfer from AS to GSC (+). We focused on GSC (+) due to their crucial role in relapse and their reliance on mitochondrial ATP synthesis^31^. We performed bulk RNA sequencing (RNA-seq) on sorted GSC (+) positive for astrocytic-derived mitochondria (mito-dsRED + ) and on GSC (+) negative (mito-dsRED-) as controls (see experimental schematic in Fig. 5A).

Differential gene expression (DGE) analysis using principal component analysis (PCA) identified two clusters: control GSC (+) with no exogenous mitochondria (mito-dsRED-, neg), and GSC (+) that received astrocytic mitochondria (mito-dsRED + , pos) (Fig. 5B). These results suggest that AS-GSC communication may alter the transcriptional program of GSCs.

We observed hundreds of differentially expressed genes between pos and neg clusters. Interestingly, there were 1982 upregulated genes and no downregulated genes (absolute log2 fold change above 1), indicating that the acquisition of astrocytic material acts as an activator rather than a suppressor in GSC (+) (Fig. 5C, and Volcano plot in 5D).

Gene Ontology (GO) enrichment analysis revealed the enrichment of genes related to mitochondrial metabolism, including electron transport coupled proton transport, regulation of membrane potential and transmembrane transport (Fig. 5 E, Supplementary Fig. 8).

KEGG pathways analysis showed that motor proteins associated with mitochondrial transport, specifically KIF6 and KIF9 (MT-based), as well as Myo3 (Actin-based), were upregulated (Fig. 5F, G). Additionally, axonal dynein DNAH, which is involved in the movement of cilia and flagella and retrograde movement of axon, was also identified.

Recipient GSCs also exhibited increased mitochondrial membrane potential (TMRM) and subtle morphological differences.These cells showed a trend toward increased cell area (961.3 ± 404.1 vs 858.2 ± 303.9 µm² in mitoGFP⁺ and mitoGFP⁻ cells, respectively) and perimeter (245.7 ± 76.2 vs 229.4 ± 70.7 µm), together with reduced circularity (0.26 ± 0.04 vs 0.31 ± 0.10) and a modestly lower aspect ratio (1.9 ± 0.2 vs 2.11 ± 0.6); however, none of these morphological differences reached statistical significance. (Supplementary Fig. 9).

### Evidence of TNT-like connections in cancer cells in vivo supporting their role in mitochondria exchange

TNT have been widely studied in vitro in several tumoral cells, in 2D- and 3D-cultures, including organoids^17^, *ex vixo* explants^43^, and tissues^27^. Visualizing the dynamics and function of these structures in live animals has been challenging. However, the development of ISMic, which offers high-resolution imaging comparable to those achieved in vitro provide now with this unique opportunity^32,33,44^.

Nonetheless, ISMic in live rodents—with the resolution required to detect TNTs and mitochondrial transfer—does not work in deep brain regions. To address this limitation, we used an orthotopic tongue cancer model for HNSCC suitable for ISMic^34,45^, as a tool to visualize TNTs in vivo.

First, we characterized TNTs in HN12 cells, a non-invasive HNSCC cell line. We found that HN12 cells form TNTs in vitro with characteristic such as percentage of TNT-connected cells (36.48 ± 14%), average TNT apparent diameter (627.08 ± 200 nm), and TNT length (12.85 ± 3 µm) very similar to U251 cells. Additionally, HN12 TNTs hovered above the dish, as shown in a 3D-rendering movie (Supplementary Fig. 10, and Movie 14).

**Fig. 6:**
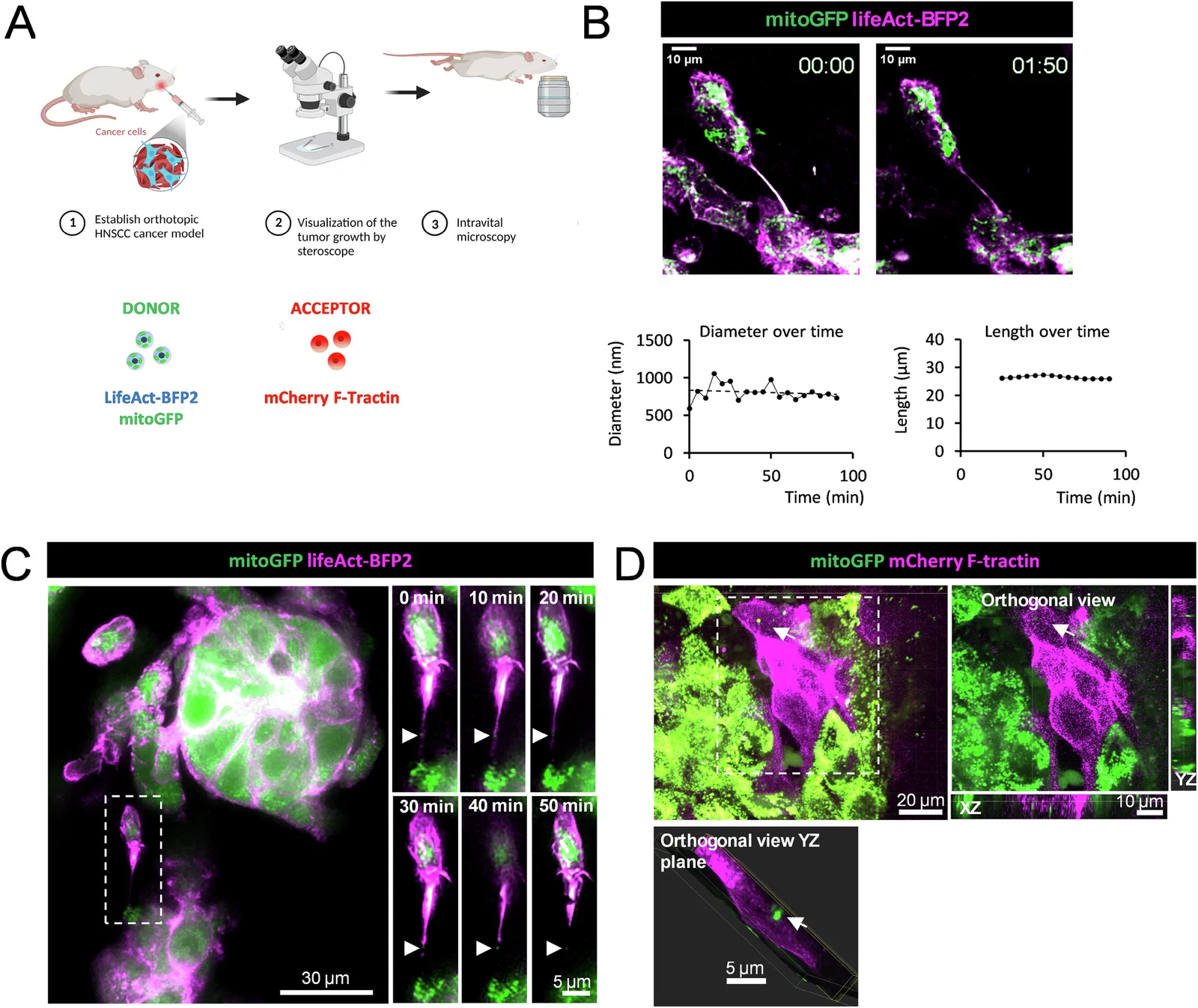
TNT-like connections between HNSCC cells in vivo. **A** Experimental design of the orthotopic HNSCC xenograft model. A 1:1 mixture of engineered HN12 donor (D) cells co-expressing mitoGFP (mitochondria, green) and LifeAct-BFP2 (actin, blue), and acceptor (Acc) cells expressing mCherry-F-tractin (actin, red), was submucosally injected into the anterior tongue of athymic nu/nu mice (500,000 cells/20 µl). Tumor growth was monitored by intravital subcellular microscopy (ISMic). **B** Representative time-lapse sequence showing a persistent TNT-like connection from one of five independent ISMic experiments (upper; Supplementary Movie 18). Bottom, TNT thickness and length over time measured using TrackMate (FIJI). Images were acquired by two-photon microscopy (750 nm excitation; actin, 405 nm emission; mitoGFP, 505–560 nm emission). Maximum projection of a 44-slice Z-stack (0.7 µm steps; 30.8 µm total thickness), acquired every 5 min (18 time points). Magnification, 40×. Scale bar, 10 µm. **C** Representative ISMic time-lapse sequence showing a TNT-like connection containing a mitoGFP⁺ mitochondrion. MitoGFP⁺ mitochondria within TNT-like connections were observed in three of five independent ISMic experiments. Right, magnified view of the boxed region. White arrows indicate mitochondrial position over time. Complete mitochondrial transfer was not observed during the 1 h recording. Maximum projections of 64 optical sections (0.7 µm steps; 44.8 µm total thickness) were acquired every 4 min for 50 min (12 time points). Magnification, 30×. Scale bar, 30 µm. **D** Single XY plane and orthogonal XZ/YZ views of the boxed region showing a mitoGFP⁺ punctum (white arrows) within an HN12 mCherry-F-tractin acceptor cell. Z-stacks were acquired sequentially at 900 nm (mitoGFP) and 1080 nm (mCherry-F-tractin), comprising 15 optical sections (0.7 µm steps; 10.5 µm total thickness). Magnification, 30×. Scale bar, 20 µm. Representative of three independent experiments (*N* = 3), consistent with mitochondrial transfer in vivo. Source data are provided for all graphs. Created in BioRender. Saenz-de-Santa-Maria, I. (2026) https://BioRender.com/lo4kjwt.

Next, to characterize the presence and functionality (mitochondrial transfer) of TNTs in HNSCC in vivo. We injected 500,000 cells of a 1:1 mixture of a D population (HN12 with labeled actin and mitochondria, mitoGFP-lifeAct-BFP2) and an Acc population (HN12 with labeled actin by F-tractin mCherry) into the ventral side of the tongue of immunocompromised mice (Fig. 6A). After tumor development, we performed ISMic on the living animal at different time points up to 40 days post-injection.

Controlling cell density is essential for visualizing and quantifying TNTs. If cells are too far apart, TNT formation is impaired; if they are too close, the TNTs are too short to observe. Most current studies on TNTs use 2D substrates in vitro, which allow for easy control of cell density by adjusting cell concentrations or employing micropatterning to modify cell distances^40^. In vivo, this process is more complex. In our orthotopic model, HN12 cells form a tumor niche resembling human patient HNSCC tissue (Supplementary Fig. 11, and Movies 15 and 16). The high cell density in this niche physically restricts cell movement, limits migration, and hinders visualization of potential TNTs between cells (Supplementary Movie 17).

Nevertheless, we successfully observed TNT-like connections in vivo. These connections exhibited structural features similar to those observed in vitro, including composition (actin filaments), thickness (805 ± 108 nm), length (25–27 µm), and lifetime ( ~ 2 h) (Fig. 6B, Supplementary Movie 18), as well as the presence of mitochondria inside the TNTs (Fig. 6C). Additionally, we visualized GFP+ puncta in Acc cells expressing F-tractin mCherry (Fig. 6D). While we were unable to monitor the complete transfer of mitochondria from D to Acc populations, the presence of mitochondria (mito-GFP + ) in Acc cells and in TNT-like structures suggested that mitochondrial transfer may occur through these structures.

**Fig. 7:**
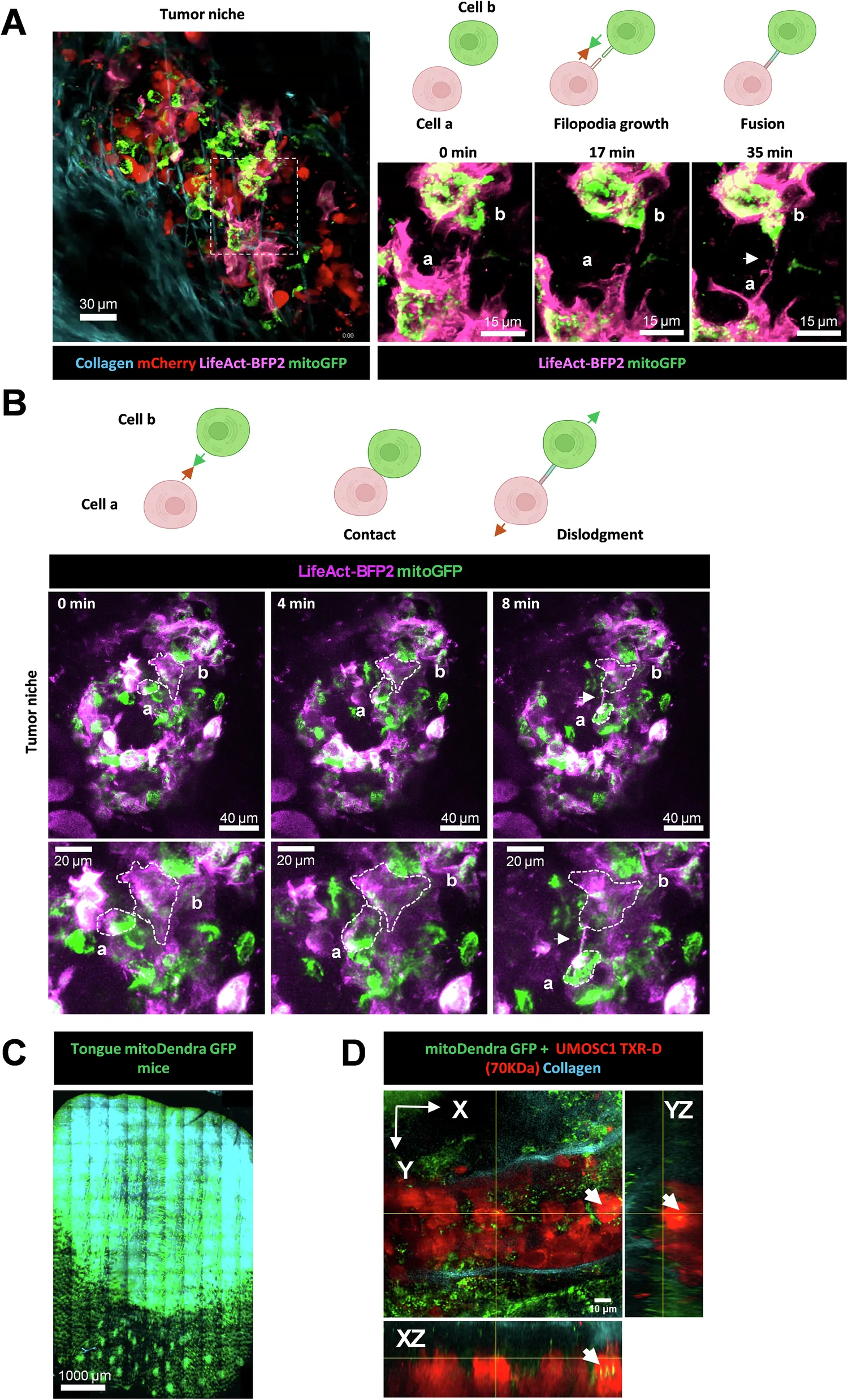
TNT-like connection formation and mitochondrial transfer from the TME to HNSCC cells in vivo. **A**, **B** Two mechanisms of TNT-like connection formation in vivo. **A** Representative ISMic time-lapse images showing TNT formation by the filopodia-growth mechanism within the tumor niche. Filopodia extending from two cells (A and B) establish contact, forming a TNT. A mitoGFP⁺ punctum is visible within the TNT at the final time point (Supplementary Movie 19). **B** Representative ISMic time-lapse images showing TNT formation by the cell dislodgement mechanism. Two adjacent cells progressively move apart while maintaining a TNT connection (Supplementary Movie 20). Insets show magnified views of the region of interest (ROI). Schematics above each panel illustrate the corresponding formation mechanism. White arrows indicate TNTs. Supplementary Movies 19 and 20 show representative qualitative observations from five independent experiments. **C**, **D** Mitochondrial transfer from the TME to HNSCC cells in vivo. **C** Representative image from one of two mito-Dendra1 mice, showing widespread GFP-labelled host mitochondria throughout the tongue. The entire tongue was imaged by two-photon microscopy (500 nm excitation, 505–560 nm emission) using 3D stitching before selection of regions of interest for high-resolution imaging. Magnification, 30×. Scale bar, 1000 µm. **D** Orthogonal reconstruction showing TXR-70-labelled 4MOSC1 HNSCC cells (red) containing host-derived GFP⁺ mitochondria (white arrows). Collagen fibers are shown in light blue. Z-stacks were acquired sequentially at 750 nm (collagen), 900 nm (mitochondria), and 1080 nm (TXR-70-labelled tumor cells). Scale bar, 10 µm. Representative qualitative observation from two independent animals (*N* = 2), consistent with host-to-tumor mitochondrial transfer. Created in BioRender. Saenz-de-Santa-Maria, I. (2026) https://BioRender.com/ggf5nhp.

Furthermore, live imaging of the tumor in the mouse tongue revealed examples or both mechanisms responsible for TNT formation, similar to those described in vitro^21^: i) actin mediated protrusion and ii) cells dislodgement (Fig. 7A-B, Supplementary Movies 19 and 20, respectively). Since we visualized TNTs from two distinct cell populations HN12 mitoGFP-lifeActBFP2 (mitochondria green, actin blue) and HN12 F-tractin-mCherry (actin in red), excluding the possibility that these connections were derived from mitotic events between daughter cells (Supplementary Movie 21).

Taken together these data support the existence of TNT in vivo likely transferring mitochondria between tumor cells. Furthermore, due to the high cell density and the inherent challenges of imaging in living animals, we probably underestimated their prevalence. Moreover, we cannot fully exclude the presence of TMs, as described by Osswald et al. (*Nature*, 2015)^26^, given the complex architecture of the tumor niche. It is therefore plausible that both TMs and TNTs coexist, with TNTs being the primary structures responsible for mitochondrial transfer, as previously reported in 3D organoids^17^.

To assess whether healthy cells from the TME would transfer mitochondria to the tumor we used a syngeneic murine tongue model based on the HNSCC mouse cell line 4MOSC1^46^ orthotopically implanted into transgenic mice expressing the mitochondria-localized probe mito-Dendra-Tom^47^(Fig. 7C). 4MOSC1 were labelled with Texas-RED (MW 70 kDa, TXR-D), which accumulates in the lysosomes and labels the cells for several days before injection into the tongue^48,49^. ISMic showed that TXR-70 HNSCC cells generated tumor in the tongue of mitoDendra1 mice, and we could observe tumor-Acc TXR + containing mitoDendra+ punta inside the cell body (Fig. 7D). This suggests that mitochondria from the TME cells were transferred to HNSCC tumor cells in vivo, serving as a proof-of-concept for monitoring this process in real time in live animals.

## Discussion

Communication between non-tumoral cells and tumors is a hallmark of cancer^50^. The interaction between tumor cells and the surrounding stromal components can drive tumor initiation^51^, progression^7,8^, and metastasis^52^. The horizontal transfer of mitochondria between different cell types in cancer or other diseases has been linked to tissue repair^53^, rescue mechanisms^11,14,54^, and enhanced tumorigenesis^3,4^ and metastasis^13,55^. Specifically, TNT-mediated transfer of healthy mitochondria has been associated with rescuing cells in early apoptotic stages^12^, correcting mitochondrial import failure^11^, and improving the metabolic fitness of T cells^6^. Moreover, various cancer types exploit TNTs to acquire mitochondria from immune cells^4^, mesenchymal stem cells (MSCs)^10^ and cancer-associated fibroblasts (CAFs)^8^—supporting immune evasion^5^, promoting tumor cell survival, increasing malignancy, and driving invasion^56^.

However, the occurrence of mitochondria transfer in cancer in the living animal, as well as the mechanisms and effects of mitochondrial exchange between the TME and cancer cells in vitro, remain under characterized.

Here, we conducted a detailed live imaging analysis to study the physical characteristics of TNTs capable of carrying mitochondria, as well as the dynamics of TNT-mediated mitochondrial transfer between GB/GSCs, and between AS and GB/GSCs in vitro. Additionally, we used ISMic to assess TNT formation in a solid tumor model in live animals.

In vitro TNTs can be classified into two main types based on their cytoskeletal composition: (1) thin and fragile TNTs composed exclusively of actin filaments (Actin-based TNTs)^20^, and (2) thick TNTs composed of both actin filaments and MT (MT-based TNTs)^19,21^. Our findings reveal significant heterogeneity in the cytoskeletal composition of GB/GSCs, with thin Actin-based TNTs being more prevalent across all cell types. We demonstrate that mitochondria are transferred horizontally via both types of TNTs, providing evidence of mitochondria integration from the D population into the mitochondrial network of the recipient cell. While these structures are consistent with TNT-like connections, we acknowledge that in the dense tumor microenvironment the coexistence of TMs, as described by Osswald et al. (2015)^26^, cannot be excluded.

In neurons, short-range movement of mitochondria occurs via the actin cytoskeleton, whereas long-distance transport is MT-dependent^57^. Consistent with this, our findings show that MT-based TNT are longer and more stable compared to the Actin-based ones.

One of the main findings of our study is that mitochondrial transfer via TNTs represents a mode of cell-to-cell communication between GB/GSC tumor cells and the AS from the TME. We demonstrate the bidirectional interchange of mitochondria via TNTs, between primary non-tumoral AS and GB/GSCs. GB/GS cells deliver disrupted mitochondria to AS, which colocalize with astrocytic lysosomes suggesting a clearing mechanism through mitophagy. Conversely, AS shed healthy mitochondria to GB/GS cells, and these mitochondria likely integrate into the mitochondrial network of recipient cells.

**Fig. 8:**
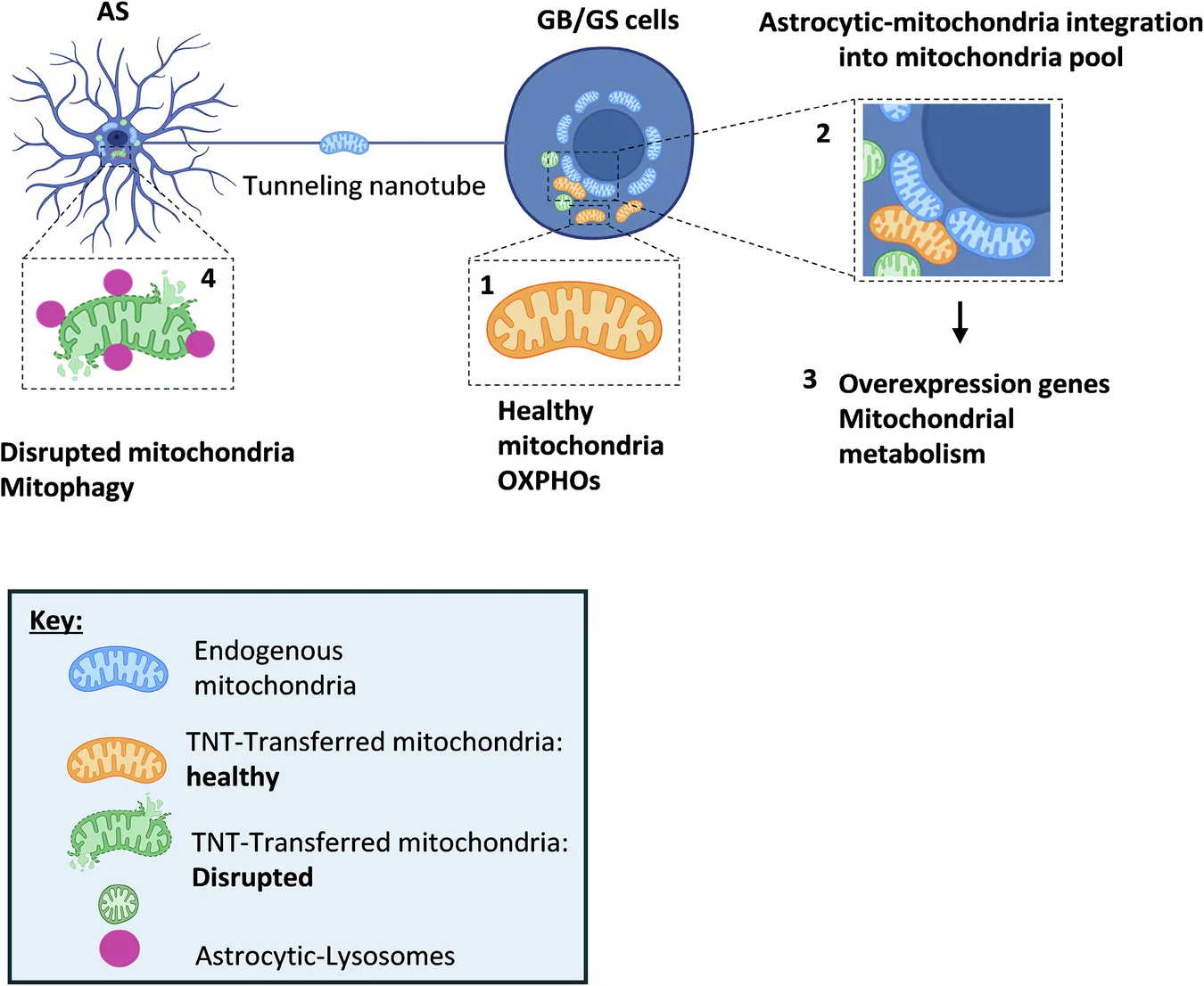
Proposed model of mitochondrial interchange between GB/GS cells and AS via TNTs. (1) TNTs mediate the transfer of healthy mitochondria and a few globular mitochondria from AS to GB/GS cells. (2) Healthy mitochondria integrate into the GB/GS mitochondrial network. (3) This promotes the expression of genes associated with mitochondrial metabolism. (4) TNTs also mediate the transfer of damaged mitochondria from GB/GS cells to AS, facilitating their clearance by astrocytes. *Created in BioRender. Saenz-de-Santa-Maria, I. (2026)* https://BioRender.com/s4ba5kt.

Our RNA bulk seq analysis of GSC containing AS-derived mitochondria reveals that transfer of heathy mitochondria (from AS to GSCs) through TNTs is associated with the upregulation of genes related to mitochondrial metabolism (see model in Fig. 8). These findings suggest that the delivery of healthy mitochondria from AS to tumor cells may contribute to increased expression of genes related to energy production and mitochondrial transport, as well as to metabolic and morphological changes consistent with enhanced oxidative states. Nonetheless, while this suggests a potential functional impact of mitochondrial transfer, we cannot exclude the possibility that pre-existing transcriptional heterogeneity in a subset of GSCs contributes to both mitochondrial uptake and the observed gene expression changes.

Nonetheless, the reciprocal transfer of damaged mitochondria from GB/GS cells to AS may also contribute functionally—possibly by offloading dysfunctional organelles to evade apoptosis and improve tumor cell survival under stress conditions.

Several studies report that horizontal mitochondrial transfer enhances the metabolic capabilities of recipient cells^3,6,10,13–15,23^, while simultaneously depleting the D population, as observed in T cells^4^. Notably, the horizontal transfer of mutated mtDNA from tumor cells to T cells has been identified as a mechanism of immune evasion through complete endogenous mitochondrial replacement^5^.

Recently, Hoover et al. described the transfer of mitochondria from neurons to breast cancer cells, demonstrating that neuronal donation of mitochondria enhances tumor metabolic plasticity and promotes metastatic potential^13^. In addition, Brisudova et al., 2025, showed that the transfer of mitochondria from AS to mitochondrially impaired GBM cells restored mitochondrial respiration in GBM cells in vivo, enabling tumor initiation and growth in the brain^14^. These findings highlight the important role of TME-derived mitochondrial transfer in cancer progression.

Together, our data indicates that GSCs not only eliminate non-functional mitochondria and acquire healthy mitochondria by communicating with neighboring AS but also promote the generation of metabolically active mitochondria. This might contribute to increased metabolic activity in cancer cells. However, it is important to note that our assessment of mitochondrial function is based on limited readouts and does not fully capture mitochondrial homeostasis. Further functional analyses will be required to comprehensively define the impact of mitochondrial transfer on recipient cell metabolism.

In addition, we cannot exclude that mitochondrial transfer may also induce stress responses in recipient cells, which could contribute to some of the observed changes.

These findings are consistent with previous reports in the central nervous system (CNS), where AS transfer healthy mitochondria to neurons following ischemic stroke as a neuroprotective effect^30^, or to GSCs, increasing GBM tumorigenicity^3,14^. Additionally, AS have been shown to degrade disrupted mitochondria from neurons in normal physiological conditions in the mouse optic nerve head in vivo, in a process called transmitophagy^58^. In Alzheimer’s disease, this neuron-AS transmitophagy increases, suggesting that TNTs may serve as a potential pathway for the transcellular transfer of disrupted mitochondria from neurons to AS^29^. Furthermore, studies outside the CNS have demonstrated that exogenous mitochondria derived from mesenchymal stromal cells (MSC) and acquired by endothelial cells trigger mitophagy rather than integrating into the endogenous mitochondrial pool^59^.

However, it is important to note that while we track the transfer of mitochondria, it is likely that other cellular materials, such as mRNA, microRNA, or lncRNAs^60^ are also transferred concomitantly. EVs also mediate horizontal mitochondrial transfer, enabling long- and short-distance communication between different cell types^61^. EV-mediated transfer has been shown from AS to neurons^30^ and to brain endothelial cells^62^. Thus, EVs and TNTs likely act as complementary mechanisms, with EVs supporting less specific exchange and TNTs mediating direct, targeted transfer.

The presence of TNTs in cancer has primarily been described in vitro or ex-vivo due to the difficulty of visualizing these thin and fragile connections with enough resolution in living animals. A breakthrough study from the Winkler’s group in 2015 showed the presence of long and thick intercellular connections, termed TM capable of transferring calcium waves, using two-Photon microscopy in live animals^26^. Recently, Watson and colleagues claimed the transfer of mitochondria from AS to GBM in vivo via TM. However, these connections were identified ex-vivo after fixation^3^, and the distinction between TNTs and TMs remains unclear. We have previously identified both TMs and TNTs in tumor organoids derived from GSCs. Our live imaging data in this 3D in vitro model suggested that only thin TNT-like connections, and not TMs, are capable of transferring mitochondria^17^. Using a similar approach in the developing zebrafish embryo, we have recently shown that TNTs capable of transferring mitochondria exist in living organism^63^.

Here we used ISMic in an oral cancer model, which allowed for less invasive tumor observation compared to the GBM model, eliminating the need for a glass window on the skull^64^. This approach reduced infection risk and minimized imaging artifacts from animal respiration, while enabling the use of higher magnification objectives for enhanced image quality. Through this method, we identified TNT-like connections between HNSCC cells in live animals. Notably, these connections share key characteristics with TNTs observed in 2D in vitro cultures, including length, thickness, and persistence. Furthermore, we observed that TNTs between HNSCC cells in vivo can form through the same mechanisms identified in vitro: cell dislodgment and filopodia-mediated growth^21^. Additionally, the presence of mitochondria within their lumen supports their role in transfer. Our data indicate that tumor cells can acquire mitochondria both from other tumor cells and from the TME, suggesting that mitochondrial exchange operates through similar mechanisms in vivo as observed in vitro with GBM cells, highlighting a critical mechanism of intercellular communication in the TME. This ISMic-based approach represents an initial step toward understanding TME-to-tumor TNT-mediated mitochondrial transfer in vivo. As a next step, a more clinically relevant model—such as the carcinogen-induced tongue cancer model—could be employed^46^.

Collectively, our findings provide strong evidence for TNT-mediated mitochondrial transfer between tumor cells and the TME both in vitro and in vivo and offer insights into its potential consequences for recipient cells.

## Methods

All research was conducted in accordance with all relevant ethical regulations. GSC (-) and GSC (+) were established from a patient enrolled in the STEMRI clinical trial (ClinicalTrials.gov Identifier: NCT01872221). The STEMRI trial was approved by the French Ethics Committee (registration No. 2012-A00585-38) and by the French Drug Administration (ANSM; registration No. B120639-40). Written informed consent was obtained from all participants. All animal procedures were approved by the National Cancer Institute Animal Care and Use Committee (NCI-ACUC, NIH) under protocol LCMB-040-2-F and were performed in accordance with the NIH Guide for the Care and Use of Laboratory Animals.

### Cell culture

The human glioblastoma cell line U-251 MG was purchased from Cytion (CLS Cell Lines Service GmbH, Germany; Cat. No. 300385). GSC (−) and GSC (+) (also known as SRC1 and SRC2, respectively), were established from a single GBM specimen obtained from a patient enrolled in the STEMRI clinical trial (ClinicalTrials.gov Identifier: NCT01872221) ³¹. GSC (−)/and GSC (+) were derived from two spatially distinct biopsies collected from the infiltrative FLAIR region identified by MRI/MRSI-guided surgery. Specifically, GSC (−) originated from the FLAIR/CNI− region, whereas GSC (+) was established from the FLAIR/CNI+ region, a metabolically more active area. Both regions, FLAIR/CNI− and FLAIR/CNI + , were enriched in GSCs. Following tissue dissociation, cells were cultured under serum-free neurosphere conditions supplemented with EGF and FGF-2 to enrich for GSC. Both cell populations formed neurosphere-like aggregates and generated stable primary and secondary neurospheres, demonstrating their self-renewal capacity. GSC cultures were characterized by RT–qPCR analysis of markers associated with different neural cell lineages, including GFAP and CHI3L1 (astrocytic markers), TUBB3 and GAP43 (neuronal markers), and OLIG1, OLIG2, SOX11, and SOX2 (oligodendroglia and stem cell markers). This gene expression profile was maintained throughout serial passaging in culture, indicating preservation of the stem cell properties characteristic of GSCs, as previously described^17^.

U251 was cultured in DMEM-F12 (1:1) medium (Gibco, 31330-038) supplemented with 10% fetal calf serum (FCS, Eurobio) and 1% penicillin/streptomycin (final concentration 100 U/mL; Thermo Fisher Scientific). GSCs were cultured in suspension in DMEM-F12 (Sigma) supplemented with B27 (50×, Gibco), N2 (100×, Gibco), and 20 ng/mL FGF-2 and EGF (Peprotech), as previously described^17^. HN12 HNSCC-derived cell line was cultured in DMEM supplemented with 10% FBS and 1% penicillin/streptomycin. 4MOSC1 syngeneic-derived cell line previously described^46^, was cultured in Keratinocyte medium supplemented with SFM Growth supplement, EGF (5 ng/ml), choler toxin (5×10^11 M) and 1% penicillin/streptomycin. All the cells were maintained at 37 °C in 5% CO2 humidified incubators. Fresh medium was added, or passages were performed to the cell culture every 2–3 days. Absence of mycoplasma contamination was verified with MycoAlertTM Mycoplasma Detection Kit (Lonza). All methods were carried out in accordance with the approved guidelines of our institution.

### Murine primary AS preparation

Primary cortical AS were prepared from C57BL/6 wild-type or ROSAmT/mGmT mice from our in-house colony (Institut Pasteur, Paris, France) and used at postnatal days 0 to 3 (P0–P3). Briefly, following decapitation of the pups, the meninges were removed from the brain, and cortices were pooled before homogenization. Dissociated cortical cells (neocortex) were cultured in 75 cm² flasks coated with 5 µg/mL poly-D-lysine (PDL) in complete AS medium, consisting of DMEM-F12 (1:1) (Gibco) supplemented with 10% FCS (Eurobio) and 1% penicillin/streptomycin (Thermo Fisher). Cultures were maintained at 37 °C in a humidified atmosphere containing 5% CO₂ until they reached confluency (7–10 days in vitro). Culture medium was refreshed every three days. To inhibit proliferation of other glial cells, a mixture of 5-fluoro-2′-deoxyuridine and uridine (10 µg/mL; Sigma-Aldrich) was added for one week. For experiments, confluent cultures were detached with trypsin and seeded onto PDL-coated multi-well plates. Before initiating experiments, AS cultures were characterized by immunostaining with rabbit anti-GFAP, a well-established AS marker (see schematic, Fig. S5 A). Rabbit anti-GFAP antibody (Dako, Cat. No. Z0334) was used for immunofluorescence at a dilution of 1:500.

### Lentiviral constructs

To label mitochondria, the lentiviral plasmids pLV-CMV-mito-GFP (Takara Bio, #632432) and pLV-CMV-DsRed-mito (Takara Bio, #632421) were used. These plasmids encode the mitochondrial targeting sequence of subunit VIII of human cytochrome *c* oxidase fused to GFP or DsRed, respectively. To visualize actin filaments in different colors, the lentiviral plasmids pLenti F-tractin-mCherry PuroR (Addgene plasmid #85131), pLenti Lifeact-mTagBFP2 PuroR (Addgene plasmid #101893), and pLenti Lifeact-iRFP670 BlastR (Addgene plasmid #84385) were used. Each construct was selected to provide distinct fluorescent markers to track actin dynamics in multicolor imaging applications. For MT visualization, L304-EGFP-Tubulin-WT (64060, Addgene) was employed. This plasmid encodes EGFP-tagged tubulin, allowing for the observation of MT structures within the cellular environment.

### Lentiviral particle production and transduction

Lentiviral particles (LVs) were generated in HEK 293 T cells cultured in DMEM-F12 (Gibco), supplemented with 10% FCS (EuroBio) and 1% penicillin/streptomycin (Thermo Fisher) at 37 °C in a humidified atmosphere containing 5% CO₂. The cells were seeded in T75 flasks the day prior to transfection to achieve 50–70% confluency. Transfection was performed using a mixture of plasmids encoding lentiviral components, specifically pCMVR8.74 (Gag-Pol-HIV1) and pMDG2 (VSV-G), along with the plasmid of interest at a ratio of 4:1:4 (μg), respectively. FuGENE HD Transfection reagent (Promega) was used according to the manufacturer’s protocol. After 48 h, LVs were concentrated using LentiX-Concentrator (Takara Bio), and the resulting pellet was resuspended in 1 mL of PBS. For transduction, 500 µL of the resuspended LVs were added directly to the target cells of interest, including U251, GSCs, HN12, or primary cortical AS, which had been plated in T75 flasks the day before infection to achieve 50–70% confluency. The cells were allowed to incubate with the LVs for a minimum of 48 h for effective transduction. Positive cells were subsequently sorted using a BD FACS Aria III cell sorter (BD Biosciences).

### Live imaging

Two microscopes were used to perform time-lapse movies and analysis on live samples in 2D-classical culture: Spinning disk X1 Metamorph RCH (SD M) and Spinning disk W1 Nikon eclipse Ti2 confocal system (SD Ti2) (Nikon Instruments, Melville, NY, USA). These microscopes are equipped with a climate box to maintain humidity at 95%, temperature at 37 °C, and 5% CO2 concentration. The utilized excitation laser was a white light laser (WLL), adjustable across wavelengths from 470 nm to 670 nm. To minimize cell stress, cell death, and photobleaching, the power of the WLL was maintained at the lowest possible level during imaging acquisition. All samples were acquired as z-stacks, with an average overall size of 7 µm, covering the whole volume of cells, either when they were alone or in co-culture, and a z-step size varying between 300 nm and 700 nm. The magnification used for all the mitochondria tracking movies and analysis of mitochondria health ranged from a minimum 40X to 100X, depending on the aim of the experiment.

Objective used:

- 60X oil immersion objective (Nikon APO60x NA = 1.4 CSU) on SD M.
- 40X water immersion objective (Apo LWD 40X NA = 1.15 WD = 610 µm); and oil immersion objective 60x (Plan Apo 60XOIL NA = 1.42 WD = 150 µm) and 100X (Plan Apo 100X OIL NA = 1.45 WD = 130 µm) on SD Ti2.

### Live-imaging analysis

The TNT dimensions, length and diameter were measured using spinning-disk confocal microscopy during live imaging of TNTs actively transferring mitochondria.

### Tracking mitochondrion movement inside the TNTs

The TrackMate program, developed by Jean-Yves Tinevez, was utilized to generate kymographs and analyze mitochondrial length and velocity within the TNTs over time, as detailed in the Data and Methods availability section of this publication in FIJI^65^. This tool can track objects inside a TNT while the connected cells migrate, providing the mitochondrial position relative to the TNT throughout the transfer process and yielding data on velocity and movement direction (anterograde, static, and retrograde).

Since the objects travel along a line following the TNT, assessing their speed is best accomplished via kymograph analysis. However, as the cells connected by the TNT move, the TNT itself changes position and size during imaging, making traditional kymograph tools inadequate. To address this, we developed a custom tool that constructs kymographs across a moving line over time. This tool relies on TrackMate^66^ to manually track the two endpoints of the TNT, and a custom extension enhances it by offering kymograph analysis and object tracking in the reference frame of the TNT.

The analyzed movies ranged from 20 min to 3 h, depending on the cytoskeletal composition, with a 2 min interval between frames. Minimal laser intensity was employed for each channel to avoid phototoxicity.

### TNT thickness/diameter over time

Most analyses of TNT thickness are conducted on fixed samples. However, TNTs are highly dynamic structures that are very sensitive to fixation. Furthermore, the fixation process can disrupt some of these connections, particularly those involving Actin-based TNTs.

In this study, TNT thickness was measured using live imaging assays with a custom Jython macro^65^ (see “Data and Methods”). Briefly, we assessed the intensity profile in the LifeAct fluorescent channel, fitting this profile with a Gaussian function to derive the TNT thickness. This approach allowed us to obtain TNT thickness measurements across different time frames of the recorded movie.

### Mean-square displacement (MSD) of mitochondria

MSD was performed on the mitochondria tracked within the TNT, using a methodology and a MATLAB tool previously developed^37^. The dataset for the analysis included the tracks of 24 mitochondria. We fitted the first 25% of the log-log of the 24 MSD curves by a straight line and kept only the fits with a R^2^ larger than 0.8. The distribution of the slopes of the fits were larger than 1 (one-sided t-test, *p* < 10^-10^, confidence interval [1.58 – ∞]), revealing that the mitochondria are undergoing active motility inside TNTs.

### Manders’ co-localization coefficients assay

The co-localization of D-derived mitoGFP with tetramethylrhodamine methyl ester (TMRM, 50 nM, active mitochondria) or Lysotracker Blue DND-22 (50 nM) in recipient cells was quantified using the JACoP plugin in FIJI, providing a measure of the overlap between two fluorescence signals, with values ranging from 0 (no overlap) to 1 (perfect overlap). After co-culturing D mitoGFP (mitochondria in green) and Acc lifeAct-670 cells (actin filaments in far-red) for 4 days, the double-positive cells were sorted, seeded overnight, and labeled with TMRM ± Lysotracker Blue. Images were acquired using SD at 37 °C and 5% CO₂. For each individual cell, fluorescence channels were extracted, and thresholds were adjusted to calculate the Manders’ co-localization coefficients.

### Mitochondrion fusion into the mitochondrial network of the acceptor cell

Movies highlighting the fusion of donor cell mitochondria with the acceptor cell mitochondria network were generated with Fiji using the Labkit annotation plugin (https://doi.org/10.3389/fcomp.2022.777728). Briefly: the donor mitochondria were manually labelled in Labkit over time, up to the fusion with a the donor cell network. This network was then also manually labelled, first forward in time after the fusion events, then backward in time up to the beginning of the movie.

### Fixed samples imaging

Fixed samples were imaged using a laser scanning confocal microscope LSM 700 (Zeiss). Images were acquired with either a 40X or a 63X oil immersion objective (zoom 0.5), both of which have a numerical aperture of 1.4, using the Zen acquisition software (Zeiss). For each sample, a 3×3 mosaic image was captured with a 10% overlap to expand the field of view. The acquired images were then processed using ICY software (Quantitative Image Analysis Unit, Institut Pasteur, http://icy.bioimageanalysis.org) or FIJI software.

### TNT counting

TNTs were identified according to the protocol of Sáenz-de-Santa-María I. et al.^67^. Cells were plated at 50% confluence for TNT visualization, at a density of 40,000 to 60,000 cells/cm². The adhesion surface was pre-coated with either poly-D-lysine (0.1 mg/ml), for AS alone or AS-U251 co-culture, or laminin (10 mg/ml, Sigma) for AS-GSCs co-cultures. The percentage of TNT-connected cells was analyzed in live, unfixed samples to assess mitochondrial presence within TNTs and to evaluate cytoskeletal composition. For general quantification of TNT-connected cells, fixed samples were stained with the membrane marker Wheat Germ Agglutinin (WGA), which enhances visualization of these structures. To preserve TNT integrity during fixation, cells were treated with solution 1 (2% paraformaldehyde PFA, 0.05% glutaraldehyde, and 0.2 M HEPES in PBS) for 15 min, followed by an additional 15 min with solution 2 (4% PFA and 0.2 M HEPES in PBS) at 37 °C.

### Morphological image analysis

Morphological image analysis (area, perimeter, circularity, and aspect ratio) was performed using FIJI. Cells were segmented from fluorescence images (LifeAct-670 channel) following background subtraction and thresholding. Binary masks were generated and analyzed using the “Analyze Particles” function. For each detected cell, area, perimeter, circularity, and aspect ratio were quantified. Segmentation results were manually curated when necessary to correct for inaccurate detection. Data were exported for downstream statistical analysis using GraphPad Prism. Outliers in area, perimeter, and aspect ratio were identified using the ROUT method (Q = 1%) implemented in GraphPad Prism and were excluded prior to statistical analysis. The number of excluded data points is indicated in the corresponding figure legends where applicable.

### Cryogenic electron tomography (Cryo-ET) data acquisition and tomogram reconstruction

The cryo-EM data were collected from multiple grids at the NanoImaging Core Facility of the Institut Pasteur using a Thermo Fisher Scientific 300-kV Titan Krios G3 cryo-transmission electron microscope equipped with a Gatan BioQuantum energy filter and K3 direct electron detector. Data acquisition was performed using Thermo Fisher Scientific Tomography software. Tomograms were acquired using a dose-symmetric tilt scheme (79), with a tilt range of ±60° and a 2° tilt increment. Tilt images were recorded in counting mode at a calibrated physical pixel size of 3.2 Å. The total electron dose over the full tilt series was 3.295 e⁻/Å², with a dose rate of 39,739 e⁻ per pixel per second and an exposure time of 1 s per tilt image. The defocus range applied was between −3 and −6 µm.

### Quantification mitochondria transfer by flow cytometry assays (FACS)

Transfer assays were performed according to the protocol of Sáenz-de-Santa-María I et al^67^. Experimental triplicates were performed for each co-culture condition. To monitor transfer by secretion in a 2D co-culture setup, D and Acc cells were co-cultured with a 1 µm filter separating them. This filter prevents direct cell-cell contact while allowing the passage of mitochondria (0.5–1 µm in diameter). For FACS analysis, cells were passed through a cell strainer to remove aggregates and then fixed in 2% PFA. FACS data were acquired on a BD LSR Fortessa flow cytometer, with GFP and far-red fluorescence detected at 488 nm, and 670 nm excitation wavelengths, respectively. Ten thousand events were acquired per condition, and data were analyzed using BD FACSDiva. FlowJo v10.1.1. Acc cells were identified as Alexa Fluor 670-positive events, whereas donor-derived mitochondria were detected by mitoGFP fluorescence. Mitochondrial transfer was calculated as the percentage of mitoGFP-positive Acc cells (double-positive events) within the total Acc cell population. Because AS displayed lower transduction efficiency and proliferated at a slower rate than GB/GS cells, the percentage of transferred mitochondria was normalized to a theoretical 1:1 donor-to-acceptor cell ratio. The normalization factor was calculated from the experimentally determined donor/acceptor ratio in each sample, and the normalized transfer efficiency was used for all statistical analyses.

### RNA-seq

GSC (+) have received astrocytic mitochondria (mito-dsRED + ) and without astrocytic mitochondria (mito-dsRED-) were sorted after 4 days of co-culture in 2D-culture with murine AS expressing mito-dsRED in a ratio of 3 D to 1 Acc (GSC +), to allow the accumulation of mitochondria, from three independent experiments. After sorting, the cells were examined by fluorescence microscopy, and RNA was extracted using the RNeasy Micro Kit (Qiagen). cDNA libraries were prepared using Illumina Stranded mRNA library Preparation Kit (Illumina) following the manufacturer’s protocol from 250 ng of RNA. Pooled libraries were additionally purified of unbound adaptors and primer dimers on AMPure XP magnetic beads (Beckman-Coulter). Sequencing was performed on a NextSeq2000 sequencing system (Illumina) to generate 40-60 million reads per sample (150 bp).

The RNA-seq analysis was performed with Sequana version 0.16.11. In particular, we used the RNA-seq pipeline (v0.19.1) available on https://github.com/sequana/sequana_rnaseq, based on Snakemake framework. The RNA-seq pipeline trimmed the reads from adapters and low-quality bases using fastp software v0.20.1. Then it mapped the reads to the Homo Sapiens genome (GRCh38 *hg38*) using STAR. Note that we decided to perform dual mapping by also including the Musculus genome because small contamination with murine AS samples were also present in the reads. For the read count and subsequent statistical analysis, mouse content was ignored. The read count matrix on Human annotation file was then used to identify differentially regulated genes. Differential expression testing was conducted using DESeq2 library 1.30.0^68^, scripts (also available in Sequana v0.16.11).

Finally, HTML reporting was available as the output of the Sequana RNA-seq pipeline. Parameters of the statistical analysis included the significance (Benjamini-Hochberg adjusted *p*-values, false discovery rate FDR < 0.05) and the effect size (fold-change) for each comparison considered. All software used within the RNAseq pipeline was containerized and is available within the Damona project (damona.readthedocs.io) and downloaded automatically by the RNA-seq pipeline (enforcing reproducibility of the analysis).

Concerning the comparison of GSC (+) pos (mito-dsRED + ) versus GSC (+) neg (mito-dsRED), genes with an adjusted *P* value of <0.05 and log2 fold change >1 were selected for an enrichment analysis. We performed GO term enrichment using the Panther database^69^. These enrichments were performed with the Sequana standalone called enrichment-panther feeding the list of differentiated genes of interest. The Panther databases was accessed through the BioServices package^70^.

The RNA-seq dataset and DE results are deposited in a public repository: https://www.ebi.ac.uk/biostudies/ArrayExpress/studies/E-MTAB-15406?key=3b85fab0-11e9-4852-aa9d-455dbff57878, :https://doi.org/10.6019/E-MTAB-15406.

### Animal experimentation

All animal procedures were approved by the National Cancer Institute Animal Care and Use Committee (NCI-ACUC, NIH) under protocol LCMB-040-2-F and were performed in accordance with the NIH Guide for the Care and Use of Laboratory Animals. Female athymic (nu/nu) nude mice or mitoDendra2 (6–8 weeks old, 20–25 g) were used in this study. Mice were housed in sterile filter-top cages under a 12 h light/12 h dark cycle at 22 ± 2 °C and 40–60% relative humidity, with food and water provided ad libitum, and were provided with a soft dough diet from the day of tumor cell injection. Tumor growth and animal health were monitored weekly throughout the study. Animals were euthanized at the predetermined experimental endpoint 40–50 days after tumor cell injection, at which time tissues were collected. No animals reached the predefined humane endpoints during the study, in accordance with the approved animal protocol. The endpoints include Hunched posture, rough hair coat; Dehydration (reduced skin turgor, sunken eyes); Rapid or labored breathing, coughing; Reduced/impaired mobility affecting the ability to obtain food or water; Pallor or cyanosis (evident in the general appearance feet, muzzle or ears which may turn white or blue); Hemorrhage or bleeding from any orifice; Diarrhea, constipation or markedly reduced intake; Signs of neurological impairment for example seizures, paralysis, circling, head tilt; Impaired ability to urinate or defecate; jaundice (yellow discoloration to ears, muzzle, or feet); Loss of > 15% normal body weight from prestudy baseline.

### Tumor endpoints

SQ Tumors: Single tumor approaching 2 cm in any one direction; Tumor Ulceration/Necrosis; Tumors interfering with normal movement impairs their ability to perform bodily functions, significantly impairs gait, or impairs their ability to obtain food or water; Tumors interfering normal bodily functions such as urination, defecation, breathing, chewing, or swallowing; Dyspnea at cage side observation

### Tongue tumor xenograft in immunocompromised athymic nu/nu mice

The tongue xenograft tumor generation was performed as previous described by Amornphimoltham et al.^45^. Briefly, female athymic nude mice (6–8 weeks old; The Jackson Laboratory) were anesthetized using isoflurane 2–5% and maintained under continuous anesthesia through a nose cone. The tip of the tongue was gently pulled out of the mouth. Half a million HN12 cells, consisting of D cells co-expressing mitoGFP-lifeAct-BFP2 and Acc cells expressing F-tractin-mCherry in 20 µl saline, were submucosally injected into the lateral anterior region of the tongue using an insulin syringe. The needle was inserted up to 1–2 mm, injecting the cells as close to the surface as possible. The animals were fed a soft dough diet from the day of injection. Tumor growth was monitored, and ISMic was performed every two days for up to 40 days (Fig. 6A). ISMic was conducted when the tumor reached 150 µm from the surface, allowing the acquisition of high-quality images.

### Syngeneic HNSCC mice model

4MOSC1 derived-cell line, previous described to recapitulates the human tobacco-related HNSCC mutanome^46^, were labeled with dextran red (TXR-D, MW70 KDa). 5 × 10⁵ cells suspended in 20 μL saline were injected into the ventral tongue of female mitoDendra2 mice (6–8 weeks old) which express all mitochondria in green (B6;129S-*Gt(ROSA)26Sor* ^*tm1.1(CAG-COX8A/Dendra2)Dcc*^/J, https://www.jax.org/strain/018397#,The Jackson Laboratory). After the development of the tumor ISMic were performed in the animal alive to visualize the transfer from the TME into de tumoral 4MOSC1 cells.

### Intravital two-photon microscopy in rodents alive (ISMic)

After the tumor developed, we performed ISMic as previous described^34^. Mice were anesthetized with intraperitoneal injections of ketamine (100 mg/kg) and xylazine (10 mg/kg). Animals were placed on a preheated stage with the mouth open, and the tongue was gently retracted using nontoothed forceps and held in a custom-made holder, secured with a glass coverslip. Body temperature was maintained at 37–38 °C using a preheated stage. Imaging was performed by using an inverted laser-scanning two-photon microscope (MPE-RS, Olympus, Center Valley, PA, USA) equipped with a tunable laser (Insight DS + , Spectra Physics, Santa Clara, CA, USA). Three excitations wavelengths to image distinct structures and fluorophores in live samples: 740 nm for collagen fibers via second-harmonic generation (SHG), 900 nm for mitochondria (stained with mito-GFP), and 1080 nm for mCherry–F-tractin and Texas Red–70 kDa dextran. Using silicon-oil immersion objectives (refractive index = 1.40), the theoretical two-photon diffraction-limited resolutions were calculated as follows. For the 30×/1.05 NA objective: ~225 nm/605 nm (lateral/axial) at 740 nm, 275 nm/745 nm at 900 nm, and 330 nm/880 nm at 1080 nm. For the 40×/1.25 NA objective: ~190 nm/425 nm, 235 nm/520 nm, and 277 nm/620 nm, respectively. These values, derived from theoretical point-spread-function limits for two-photon excitation, define the apparent spatial resolution attainable under our imaging conditions.

Emitted light was collected by an appropriate set of mirrors and filters on 3 GaAsP detectors (bandpass filters: Blue = 410–460 nm, Green = 495–540 nm, Red = 575–645 nm). Images were acquired using 37 °C heated objectives: 30X and 40X silicone oil immersion UPLSAPO objectives (NA 1.05 and 1.25, respectively), from Olympus. The first detector collagen fibers through SHG. The second captured mitoGFP and lifeAct-BFP2 signals, while the third detected mCherry and TXR-70. For time-lapse imaging of the live animals, the acquisition speed was set to 2 or 5 min of intervals, and duration up to 2 h.

### Imaging post-acquisition procession: deconvolution and 3D-rendering

Background noise in ISMic movies was reduced by applying a 2 × 2 pixel low-pass filter to each image for one or two rounds using Metamorph software (Molecular Devices). Images were then deconvolved using the Huygens Professional Deconvolution program (Scientific Volume Imaging B.V., Hilversum, Netherlands), which calculated a theoretical point spread function (PSF) based on the microscopy parameters. Deconvolution was performed with an interactive Classical Maximum Likelihood Estimation (CMLE) algorithm. For 3D volume rendering, Imaris 9.8.2 64-bit (Bitplane) was used. Drift correction was applied using the Correct 3D Drift plugin in FIJI. Final preparation of movies and images was managed with FIJI software.

### Statistics and reproducibility

Statistical analyses and graphs were generated using GraphPad Prism version 9 (GraphPad Software). Data distribution was assessed using the Shapiro–Wilk normality test. Data are presented as mean ± SD for normally distributed datasets and median for non-normally distributed datasets unless otherwise indicated. Comparisons between two groups were performed using two-sided unpaired Student’s *t*-tests for normally distributed data or two-sided Mann–Whitney U tests for non-normally distributed data. Comparisons among more than two groups were performed using one- or two-way ANOVA followed by Tukey’s or Šídák’s multiple-comparisons tests, or by Kruskal–Wallis tests followed by Dunn’s multiple-comparisons tests, as appropriate. Correlations were assessed by linear regression, categorical distributions by χ² tests and differential gene expression by the Wald test implemented in DESeq2 with Benjamini–Hochberg correction for multiple testing. Exact *P* values are reported whenever possible, and statistical significance was defined as *P* ≤ 0.05. No statistical method was used to predetermine sample size. No data were excluded from the analyses, except for predefined outliers identified using the ROUT method (*Q* = 1%) for Manders’ colocalization coefficient analyses. The experiments were not randomized. The investigators were not blinded to allocation during experiments or outcome assessment. The number of biological replicates (*N*), technical replicates (*n*), sample sizes, statistical tests, and exact *P* values are provided in the corresponding figure legends.

### Reporting summary

Further information on research design is available in the Nature Portfolio Reporting Summary linked to this article.

## Supplementary information

Supplementary Information

Description of Additional Supplementary Files

Supplementary Movie 1

Supplementary Movie 2

Supplementary Movie 3

Supplementary Movie 4

Supplementary Movie 5

Supplementary Movie 6

Supplementary Movie 7

Supplementary Movie 8

Supplementary Movie 9

Supplementary Movie 10

Supplementary Movie 11

Supplementary Movie 12

Supplementary Movie 13

Supplementary Movie 14

Supplementary Movie 15

Supplementary Movie 16

Supplementary Movie 17

Supplementary Movie 18

Supplementary Movie 19

Supplementary Movie 20

Supplementary Movie 21

Reporting Summary

Transparent Peer Review file

## Source data

Source Data

## Data Availability

The data, materials, and software supporting the findings of this study are described in the main text, figures, Supplementary Information, and the Materials and Methods section. The tool to track mitochondria in TNTs is publicly available as a TrackMate extension in the FIJI software platform. It can be freely installed by subscribing to the *TrackMate-Kymograph* update site in the FIJI updater. All data supporting the findings of this study are available within the paper, supplementary material, and Source data files. The RNA-seq dataset and DE results are deposited in a public repository: https://www.ebi.ac.uk/biostudies/ArrayExpress/studies/E-MTAB-15406?key=3b85fab0-11e9-4852-aa9d-455dbff57878 (https://doi.org/10.6019/E-MTAB-15406). Source data are provided with this paper.
