# Supplementary material for: Tunneling nanotubes regulate mitochondrial homeostasis between glioblastoma and astrocytes, and between tumor cells in vivo
Source: Nat Commun. 2026 Sep 15;17:9788. doi: 10.1038/s41467-026-76619-9 (PMC13578765; doi:10.1038/s41467-026-76619-9)
Supplement: Supplementary file 1 [file 41467_2026_76619_MOESM1_ESM.pdf]

Supplementary Fig. 1

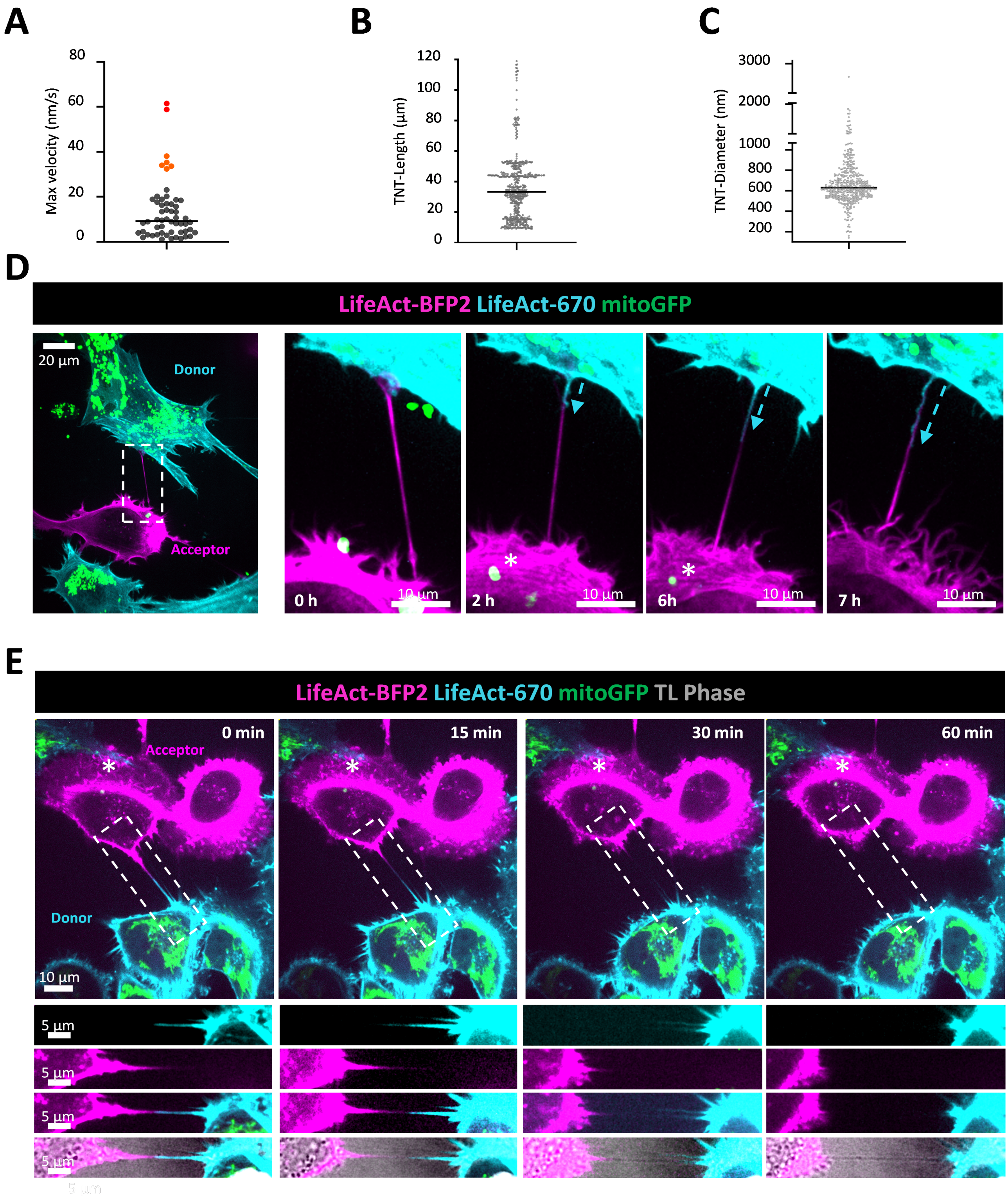

Supplementary Fig. 1 | Event-level measurements underlying the quantitative analysis of mitochondrial transfer through TNTs and representative examples of TNT formation between non-daughter U251 GBM cells in vitro. (A) Maximum mitochondrial velocity during TNT-mediated transfer. Each dot represents one tracked mitochondrion (56 mitochondria from 16 TNTs; *n* = 12 independent biological experiments). Horizontal line, median (9.26 nm s^{-1}). Red dots indicate the highest velocity recorded (61.53 nm/s). **(B)** TNT length over time. Dots represent individual measurements (530 measurements from 16 TNTs; *n* = 12 independent biological experiments). Horizontal line, median (33.32 µm). **(C)** Apparent TNT diameter over time. Dots represent individual measurements (463 measurements from 16 TNTs; *n* = 12 independent biological experiments). Horizontal line, median (630 nm. **(D, E)** U251 donor (D) cells co-expressing pLV-mitoGFP and pLV-LifeAct-670 (mitochondria and actin, green and cyan, respectively) were co-cultured with U251 acceptor (Acc) cells expressing pLV-LifeAct-BFP2 (actin, magenta). **(D)** Time-lapse images showing a protrusion extending from a D cell (cyan) along a pre-existing protrusion of an Acc cell (magenta), resulting in TNT formation (dashed cyan arrows; Supplementary Movie 3). **(E)** Time-lapse images showing D and Acc cells remaining connected by a TNT while moving apart (Supplementary Movie 4). Bottom, magnified views and corresponding fluorescence channels. White asterisks indicate transferred mitochondria. Scale bars, 10 µm (top) and 5 µm (bottom). Panels D and E are representative of three independent biological experiments. Graph data are provided in the Source Data file.

Supplementary Fig. 2

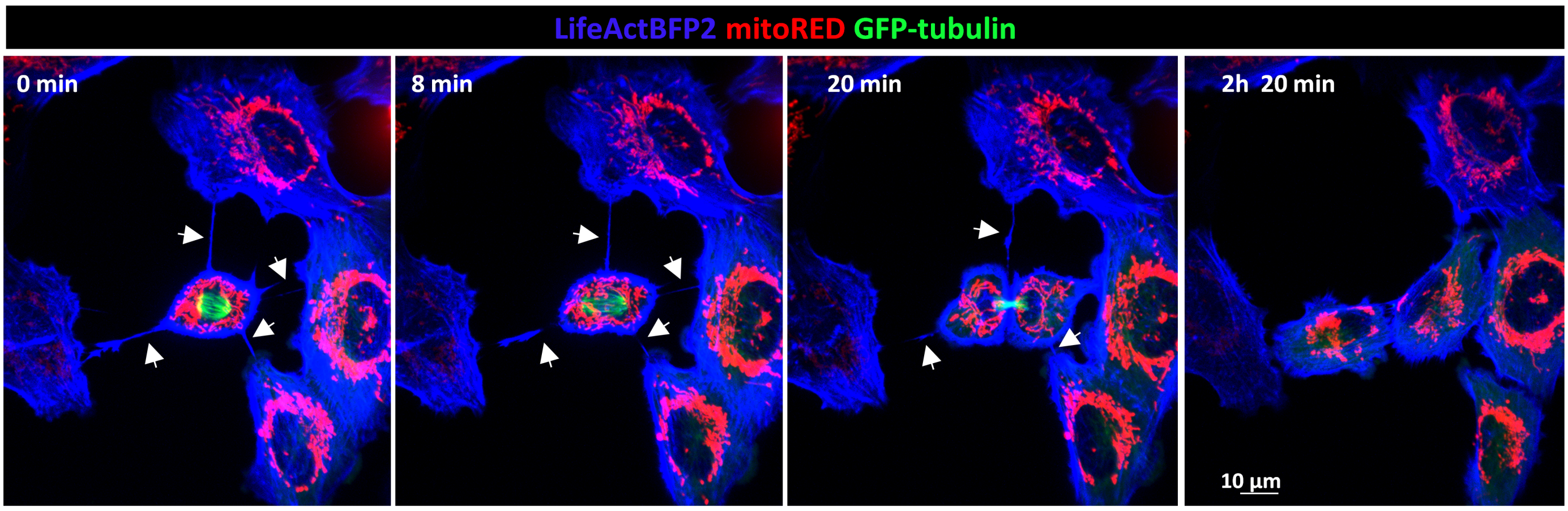

Supplementary Fig. 2 | Fluorescent reporter expression does not impair mitosis in U251 cells. Representative time-lapse images of U251 cells co-expressing pLV-LifeAct-BFP2 (actin, blue), pLV-GFP-tubulin (green), and pLV-mitoDsRed (mitochondria, red) undergoing normal mitosis, demonstrating that expression of these constructs does not interfere with cell division. White arrows indicate Actin-based TNTs connecting the dividing cell with neighboring cells throughout mitosis. No mitochondrial transfer was observed through these TNTs (Supplementary Movie 5). Scale bar, 10 µm. Representative of three independent biological experiments.

Supplementary Fig. 3

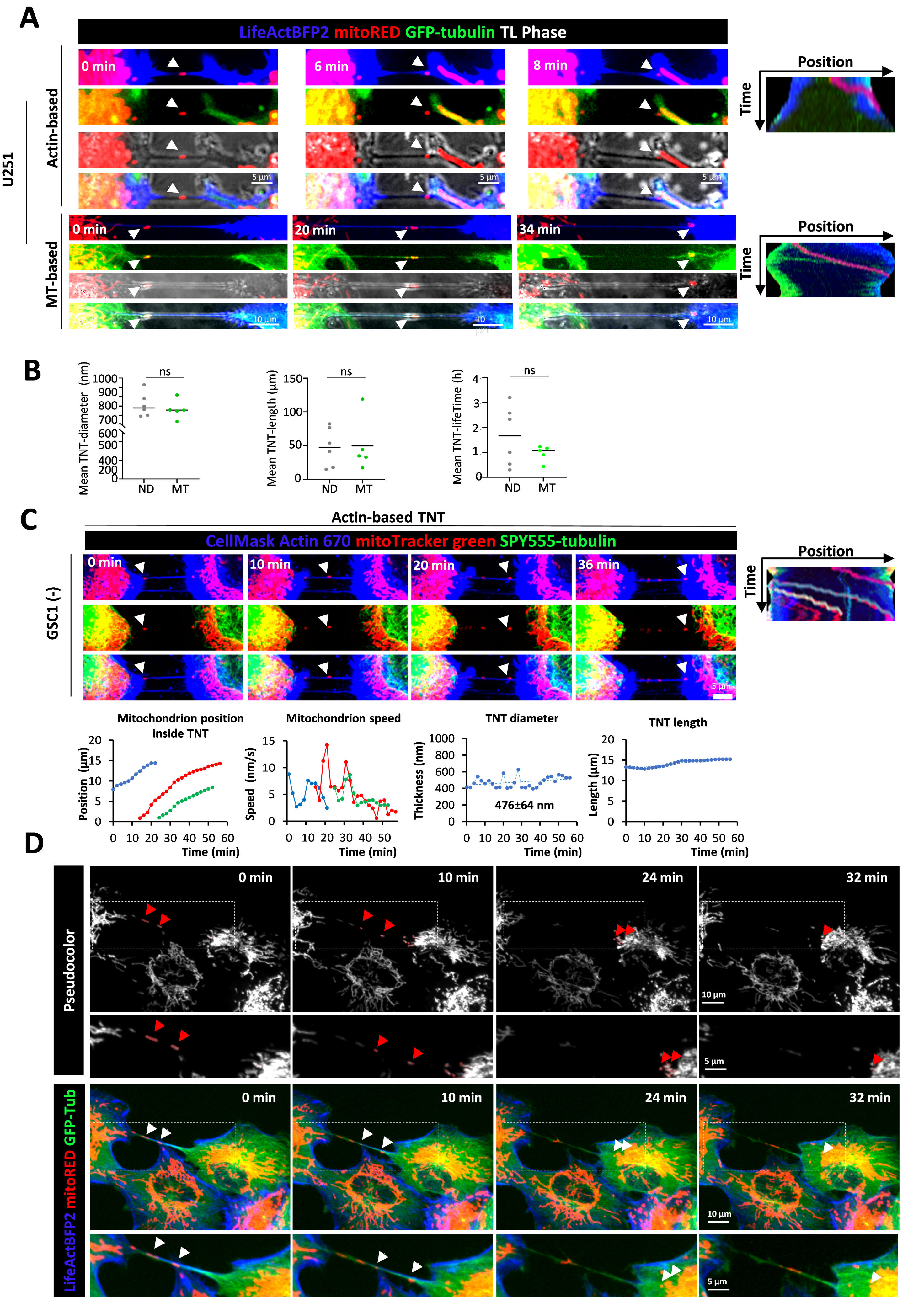

Supplementary Fig. 3 | Tracking mitochondrial transfer through Actin- and MT-based TNTs. **(A)** Representative time-lapse images of mitochondrial transfer through Actin- and MT-based TNTs in U251 cells (Supplementary Movies 6 and 8). White arrows indicate migrating mitochondria. Right, corresponding kymographs generated using the TrackMate plugin. A complete Actin-based TNT-mediated transfer was captured in one of six independent live-cell imaging experiments. Representative MT-based TNT-mediated transfer is shown from one of five complete transfer events captured across six independent live-cell imaging experiments. **(B)** Mean apparent TNT diameter (left), TNT length (middle) and TNT lifetime (right) per independent biological experiment. Each dot represents one independent biological experiment (ND-TNTs: n = 6 experiments, 10 TNTs; MT-based TNTs: n = 5 experiments, 5 TNTs). Horizontal lines indicate the median. Diameter: ND, 680.4 nm (range, 591.5–928.6 nm); MT, 657.2 nm (range, 535.4–819.4 nm). Length: ND, 47.47 µm; MT, 34.72 µm. Lifetime: ND, 1.67 h; MT, 1.07 h. Mann–Whitney U test, ns. **(C)** Representative time-lapse images of GSC(−) cells labelled with CellMask Actin 670 (actin, blue), SPY555-Tubulin (microtubules, green) and MitoTracker Green (mitochondria, red). Mitochondria move through an Actin-based TNT from donor to recipient cells (Supplementary Movie 7). White arrows indicate migrating mitochondria. Right, corresponding kymograph generated using the TrackMate plugin. Bottom, mitochondrial position, speed, TNT apparent diameter and TNT length over time. Scale bar, 5 µm. A complete Actin-based TNT-mediated transfer was captured in one of three independent live-cell imaging experiments. **(D)** Pseudo-colour time-lapse images (top) and corresponding fluorescence images (bottom) showing a transferred mitochondrion moving through a TNT and fusing with the recipient mitochondrial network in U251 (red arrowheads; Supplementary Movie 11). Fusion of transferred mitochondria into the recipient mitochondrial network was observed in five of twelve independent live-cell imaging experiments. Exact P values and summary statistics are provided in the Source Data.

Supplementary Fig. 4

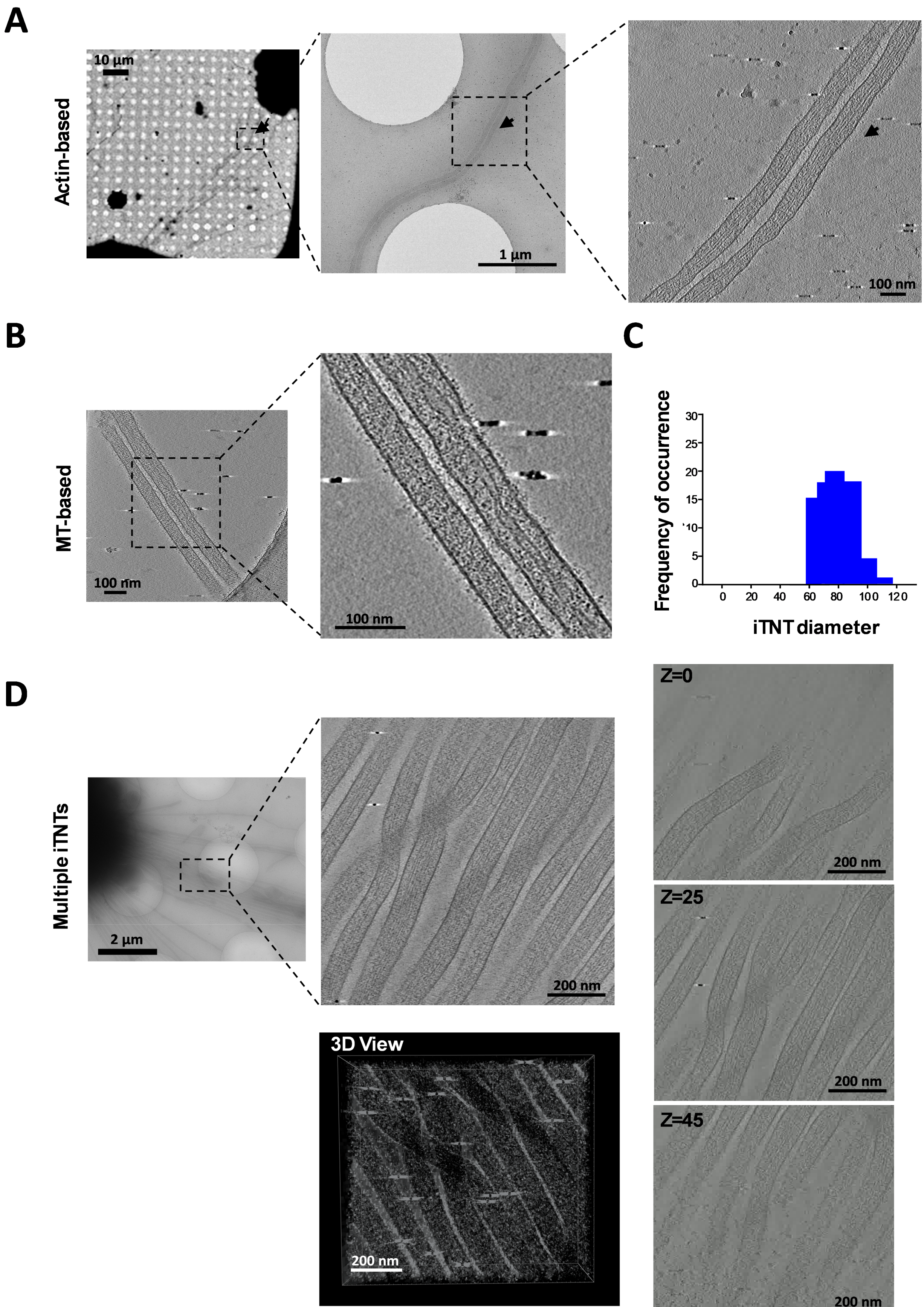

Supplementary Fig. 4 | Ultrastructure of U251 TNT-like connections. (A, B, D) Cryo-EM slices showing TNT-like structures composed of multiple individual TNTs (iTNTs). **(A)** Representative Actin-based TNT shown at low, intermediate, and high magnification. Boxes indicate enlarged regions. Scale bars: 1 μm (left) and 100 nm (right). **(B)** Representative high-magnification cryo-EM slice of a microtubule (MT)-based TNT composed of two iTNTs, one of which appears to contain microtubules. **(C)** Quantification of iTNT diameter. **(D)** Representative TNT bundle comprising up to 14 iTNTs (Supplementary Movie 9), shown as Z-stack views and a 3D rendering highlighting crossing iTNTs. Bundles reached diameters of approximately 1 μm, consistent with the thickest TNTs observed by spinning-disk microscopy. Scale bar, 200 nm. A total of 27 cryo-ET datasets were analyzed, of which 24 contained iTNT bundles and 3 showed single TNTs. Graph data are provided in the Source Data file.

Supplementary Fig. 5

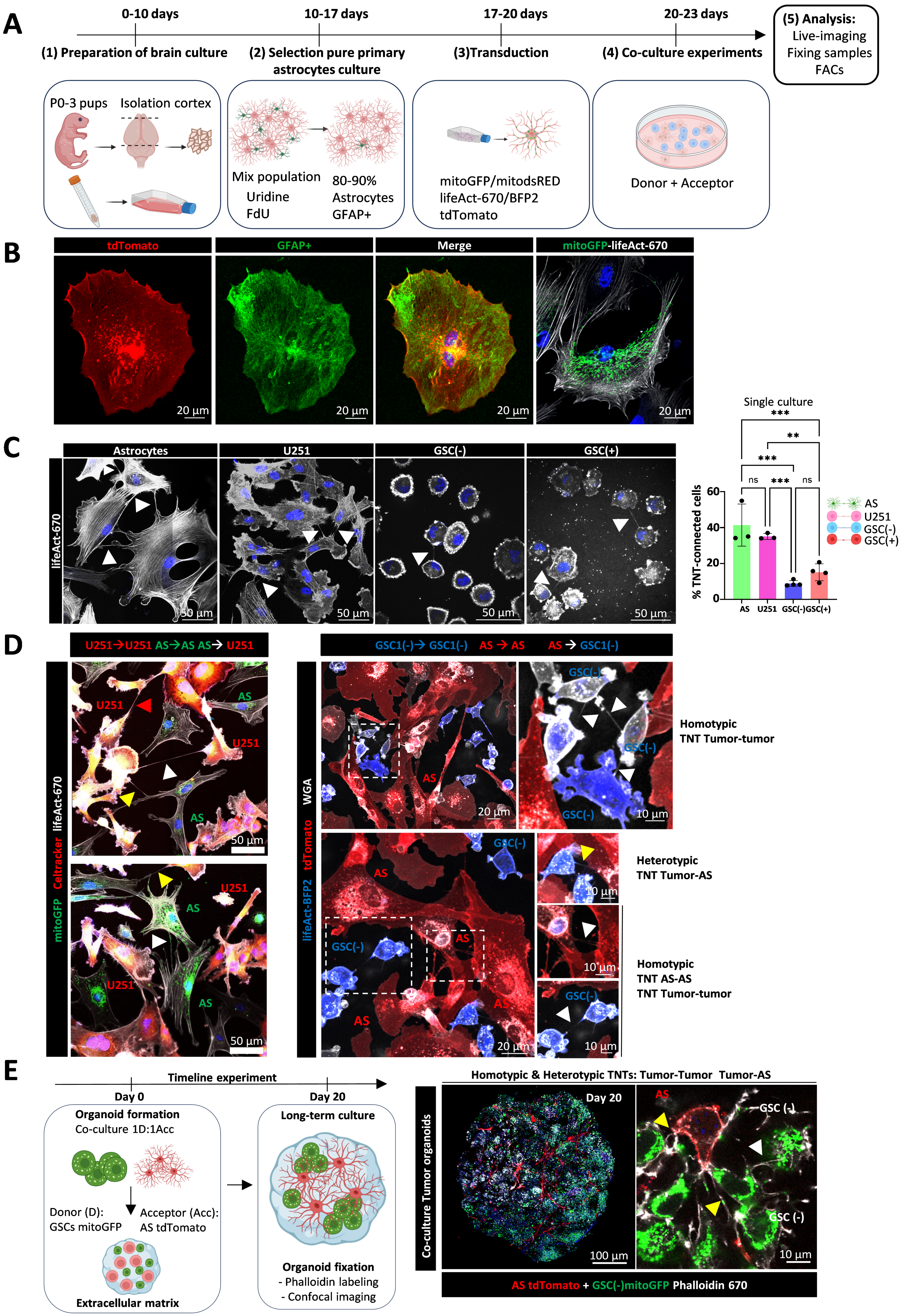

Supplementary Fig. 5 | Co-culture of GB/GS cells with primary cortical astrocytes (AS) in 2D and 3D tumor organoids. (A) Experimental workflow for the isolation, characterization, lentiviral transduction, and co-culture of primary cortical astrocytes (AS) with GB/GS cells. Created in BioRender. **(B)** Representative Z-stack projections of primary cortical AS isolated from ROSAmT/mG mice (tdTomato, red) immunolabelled for GFAP (green). Right, representative AS expressing pLV-LifeAct-670 and pLV-mitoGFP (actin, white; mitochondria, green). Scale bar, 20 μm. Representative qualitative observation from three independent experiments (*N* = 3). **(C)** Representative images of TNT-connected cells in the indicated cell types expressing pLV-LifeAct-670. White arrowheads indicate TNTs. Scale bar, 50 μm. AS and U251 cells exhibited a higher percentage of TNT-connected cells than GSC(−) and GSC(+) cells. Data are presented as the mean ± s.d. from three independent experiments for AS and U251 (*N* = 3) and four independent experiments for GSC(−) and GSC(+) (*N* = 4). One-way ANOVA with Tukey's multiple-comparisons test. **(D)** Representative co-cultures of U251 cells (CellTracker Red, left) or GSC(−) cells (blue, right) with primary AS expressing LifeAct-670/mitoGFP (white/green) or tdTomato (red). Homotypic (tumor–tumor or AS–AS; white arrowheads) and heterotypic (tumor–AS; yellow arrowheads) TNTs are shown. Insets correspond to the boxed regions. Scale bars: 50 μm (overview), 20 μm (insets), and 10 μm (higher magnification). Representative qualitative observation from three independent experiments (*N* = 3). **(E)** Experimental timeline for the generation of 3D tumor organoids (left). Representative 20-day organoid containing GSC(−) mitoGFP (green) and primary AS tdTomato (red), stained with phalloidin-670 (actin, white). Scale bar, 100 μm. Higher-magnification (40×) images show homotypic GSC–GSC and heterotypic GSC–AS TNTs. White arrowheads indicate TNTs, and yellow arrowheads indicate transferred mitochondria. Scale bar, 10 μm. Representative qualitative observation from three independent experiments (*N* = 3). Exact *P* values and summary statistics are provided in the Source Data. Created in BioRender. Saenz-de-Santa-Maria, I. (2026) https://BioRender.com/51bgktv.

Supplementary Fig. 6

FACS gating strategy to assess the percentage of acceptor (Acc) cells receiving mitochondria (corresponding to Figure 3)

A

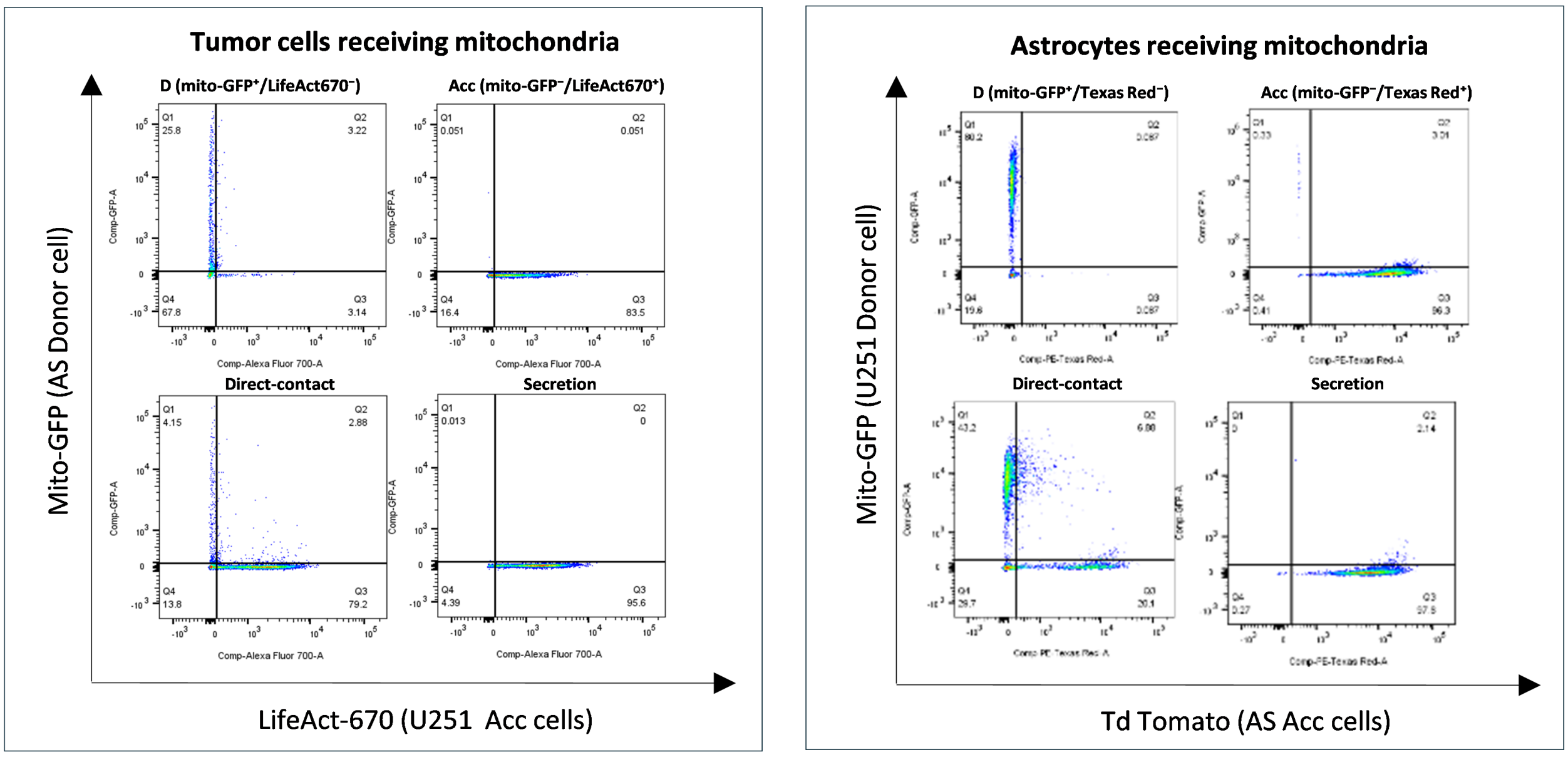

B

Sorting gate to assess the state and fate of transferred mitochondria (corresponding to Figure 4)

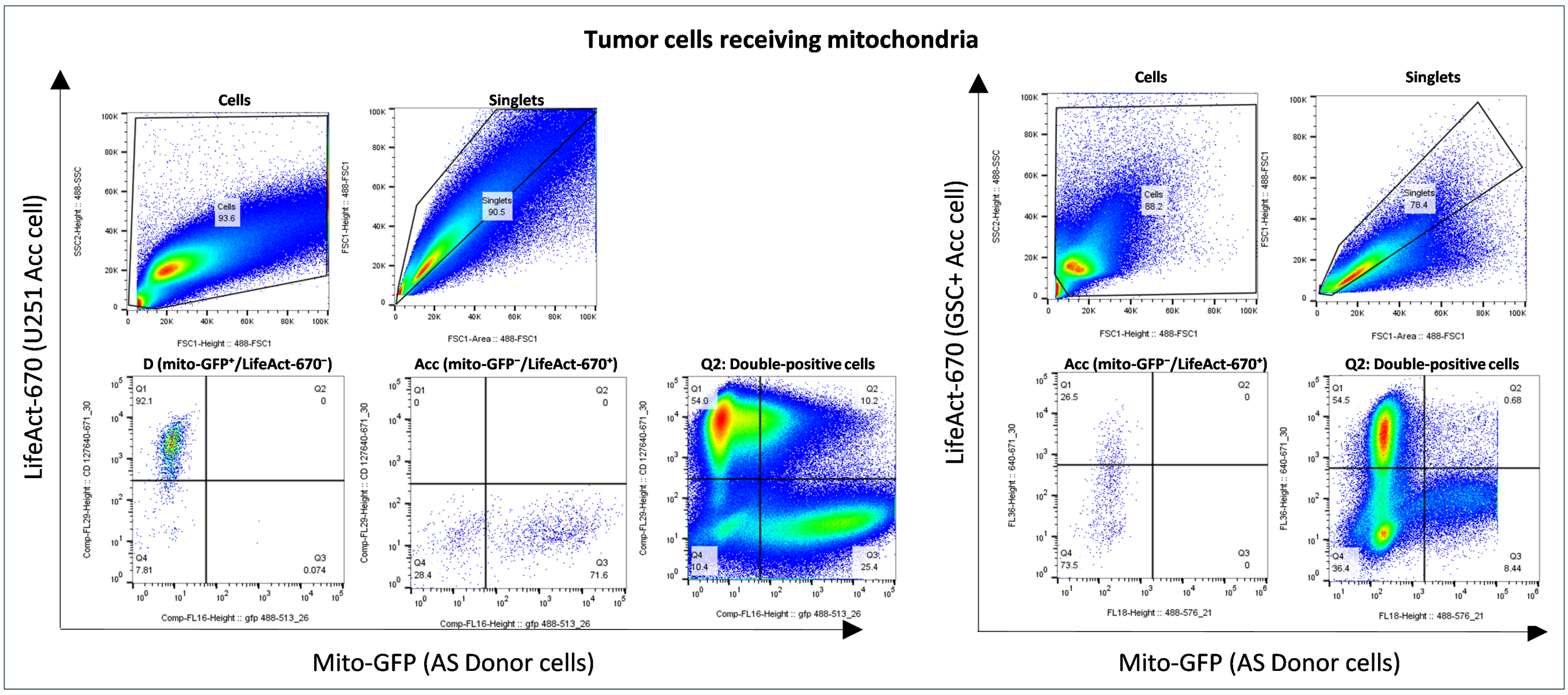

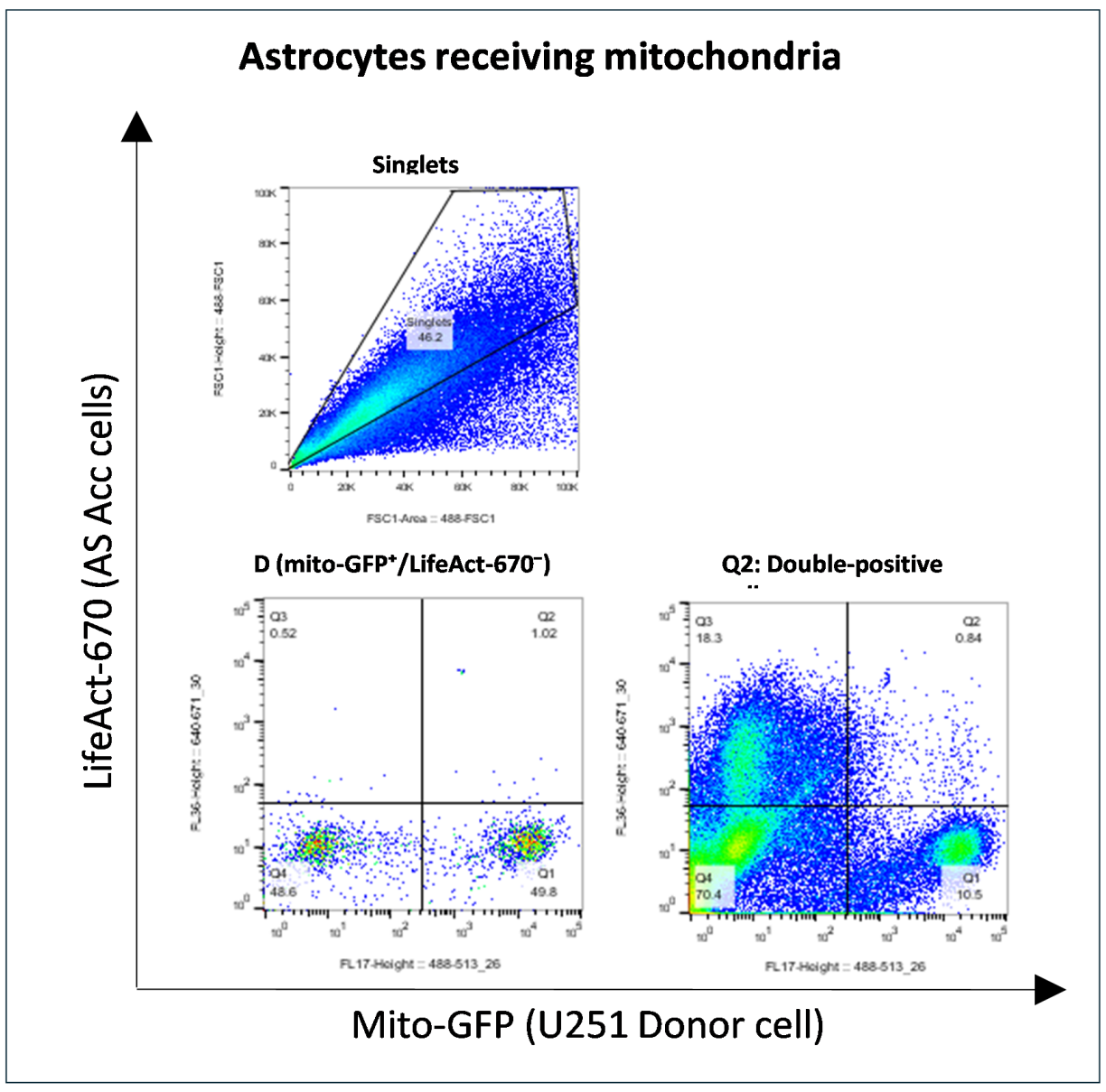

Supplementary Fig. 6 | Flow cytometry gating strategies for mitochondrial transfer quantification and acceptor cell sorting. (A) Representative gating strategy used to quantify mitochondrial transfer in the experiments shown in Fig. 3. After exclusion of debris and doublets, donor (D) and acceptor (Acc) populations were identified based on mitoGFP, LifeAct-670, or tdTomato fluorescence. Double-positive (Q2) events correspond to Acc cells containing donor-derived mitochondria (mitoGFP$^+$). Representative plots are shown for direct-contact and secretion-control assays, as well as for astrocyte-to-tumor and tumor-to-astrocyte mitochondrial transfer. **(B)** Gating strategy used to isolate acceptor cells containing donor-derived mitochondria for the analyses shown in Fig. 4. After exclusion of debris and doublets, D (mitoGFP$^+$/LifeAct-670$^-$) and Acc (mitoGFP$^-$/LifeAct-670$^+$) populations were identified, and the double-positive Q2 acceptor population was sorted for downstream analyses of the state and fate of transferred mitochondria.

Supplementary Fig. 7

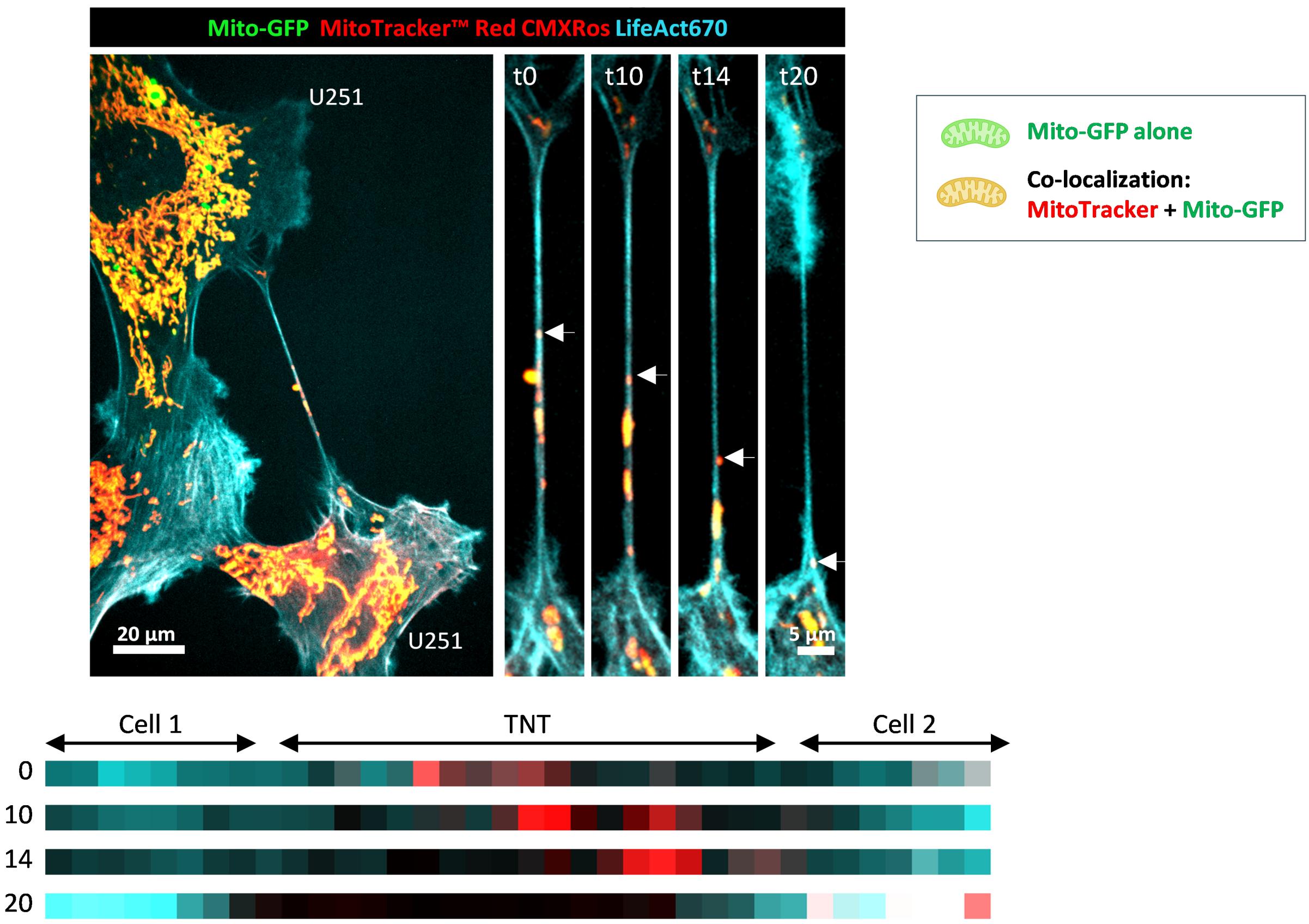

Supplementary Fig. 7 | Direct assessment of mitochondrial health during transfer between U251 cells via TNT-like connections. Representative time-lapse images of U251 cells co-expressing mitoGFP (mitochondria, green) and LifeAct-670 (actin, cyan) and stained with MitoTracker™ Red CMXRos (active mitochondria, red). Yellow mitochondria traveling through TNT-like connections indicate colocalization of mitoGFP and MitoTracker™ Red, consistent with active mitochondria. Left, magnified views of the TNT at the indicated time points. Bottom, heatmap illustrating mitochondrial transfer dynamics (Supplementary Movie 13). Representative qualitative observation from two independent experiments (*N* = 2).

Supplementary Fig. 8

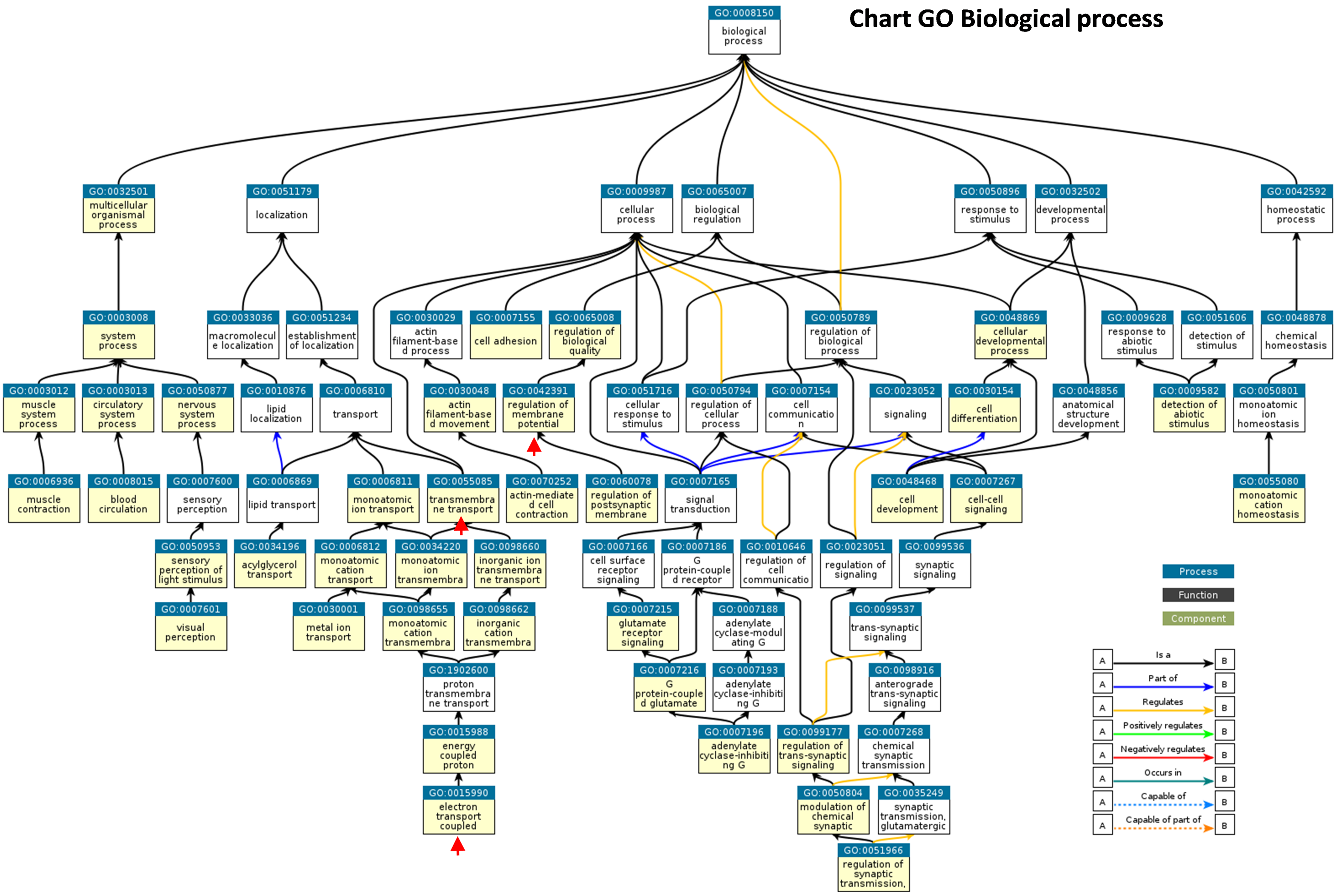

Supplementary Fig. 8 | Gene Ontology (GO) Biological Process enrichment analysis of genes upregulated in GSCs receiving astrocyte-derived mitochondria. Directed acyclic graph (DAG) showing significantly enriched GO Biological Process terms among genes upregulated in mito-dsRED$^+$ GSCs (GSC pos) compared with mito-dsRED$^-$ GSCs (GSC neg). GO terms related to mitochondrial function and bioenergetics, including electron transport-coupled proton transport, regulation of membrane potential, and transmembrane transport, are highlighted in yellow. These enriched pathways support enhanced mitochondrial respiration and metabolic activity following astrocyte-to-GSC mitochondrial transfer.

Supplementary Fig. 9

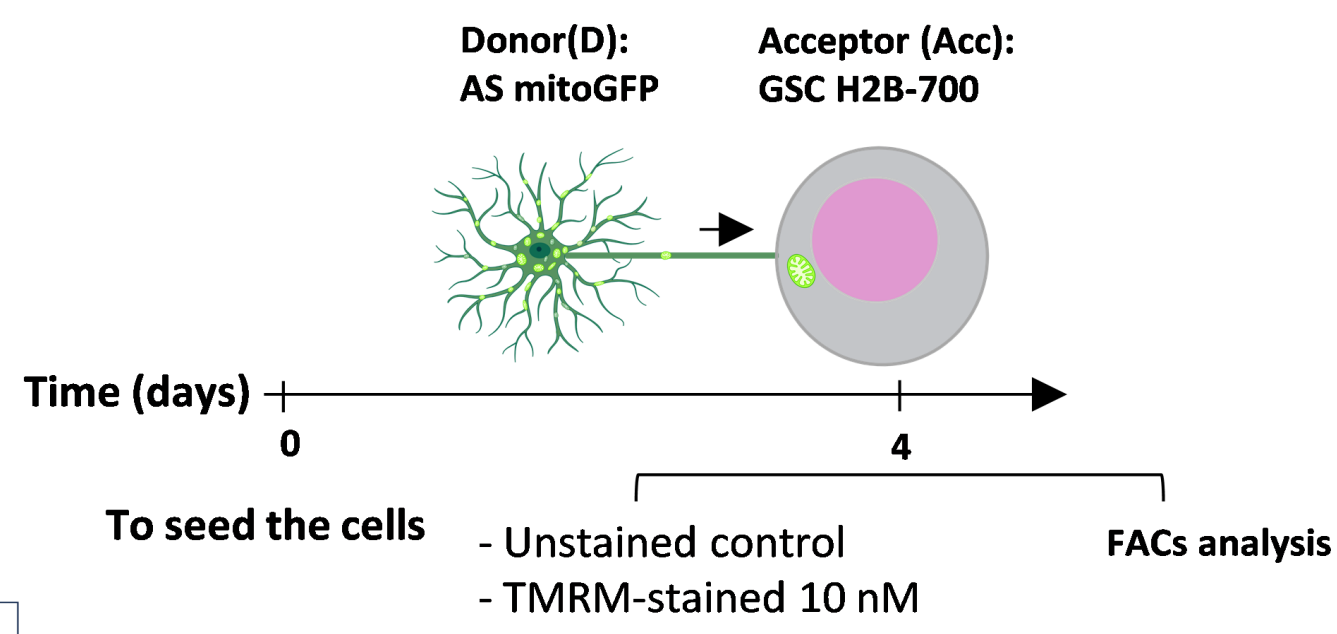

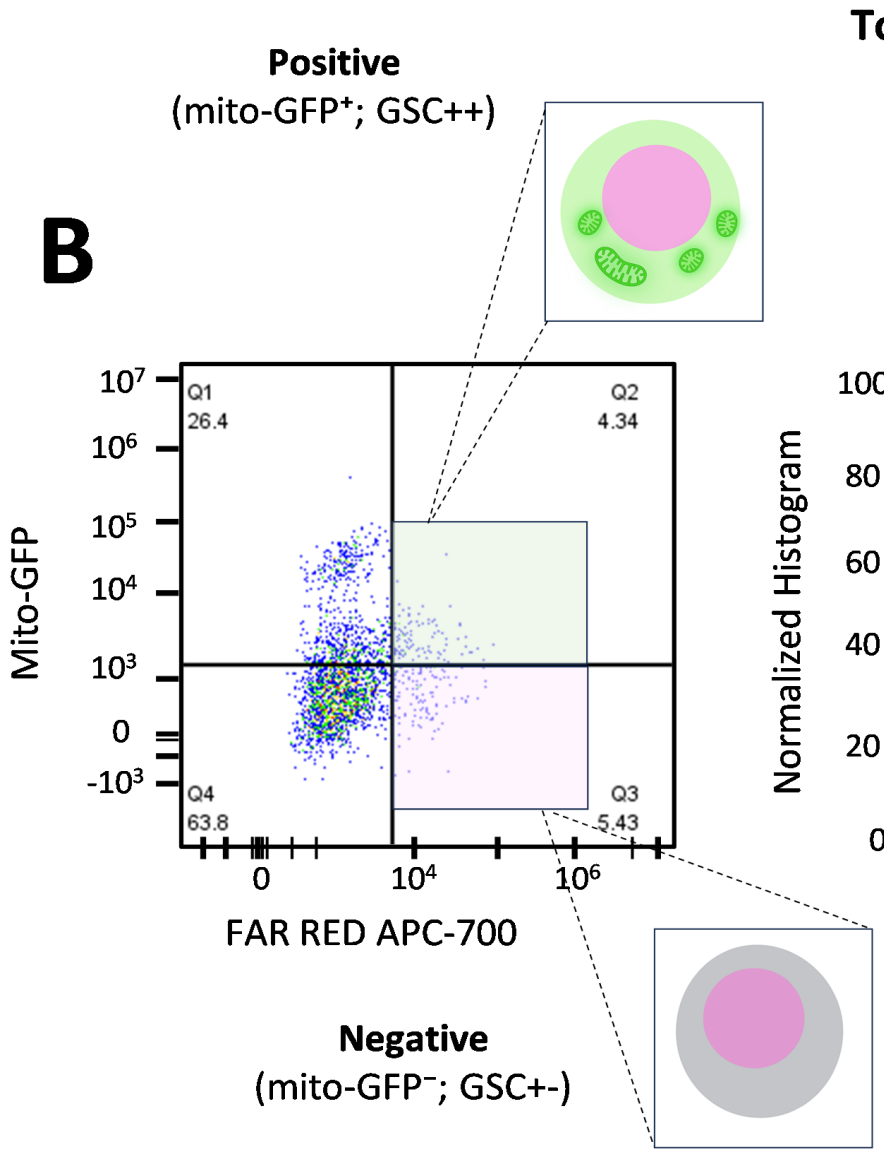

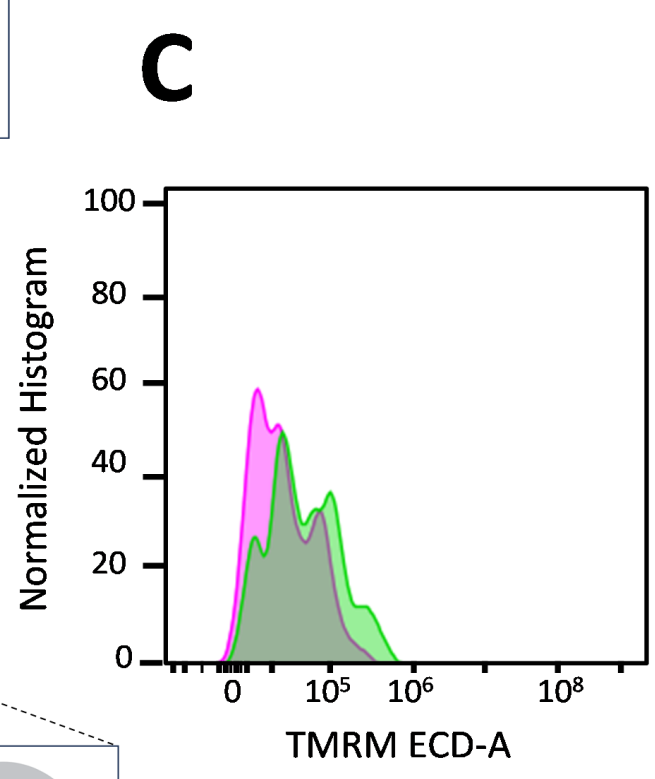

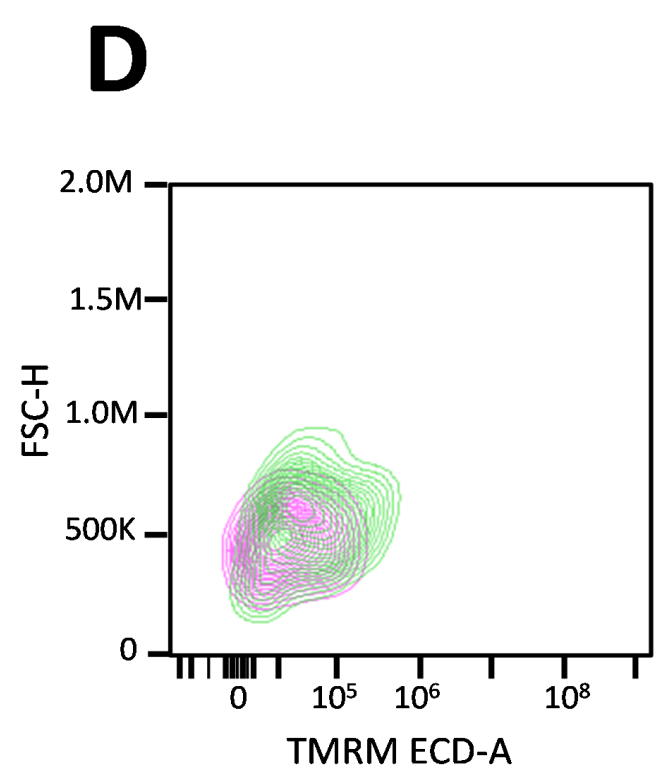

GSC mito-GFP	TMRM MFI (mean± s.d.)
Negative	36,105 ± 2,512
Positive	62,589 ±10,671

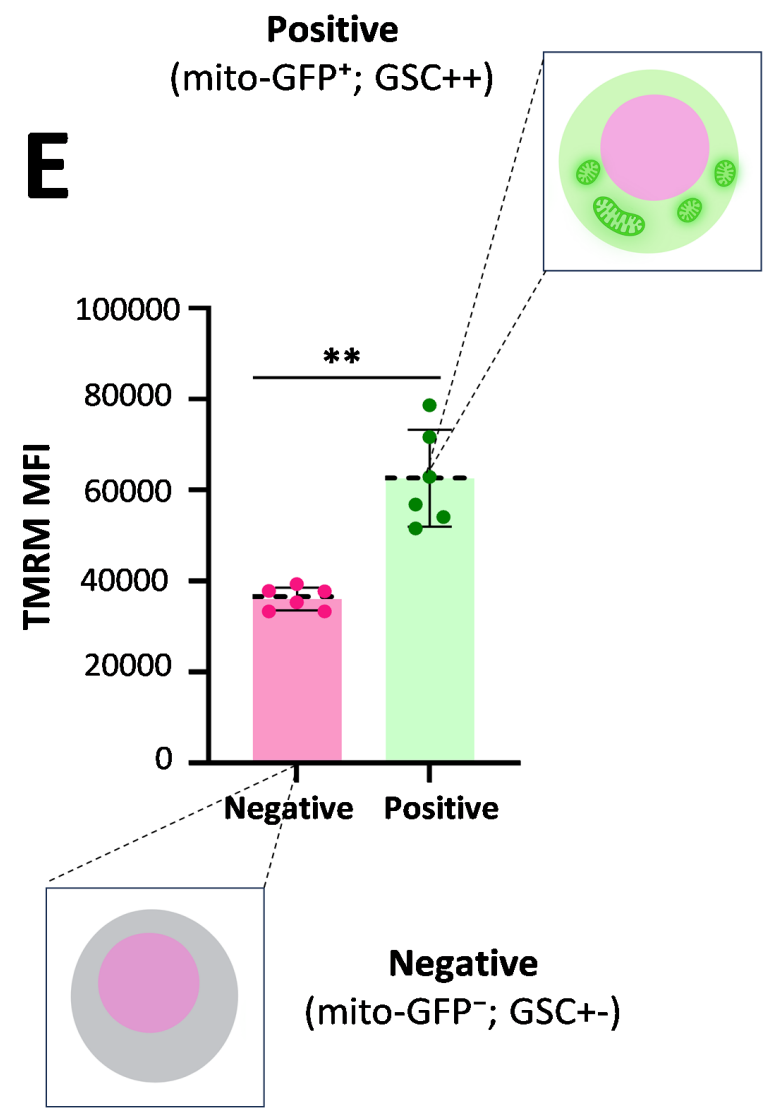

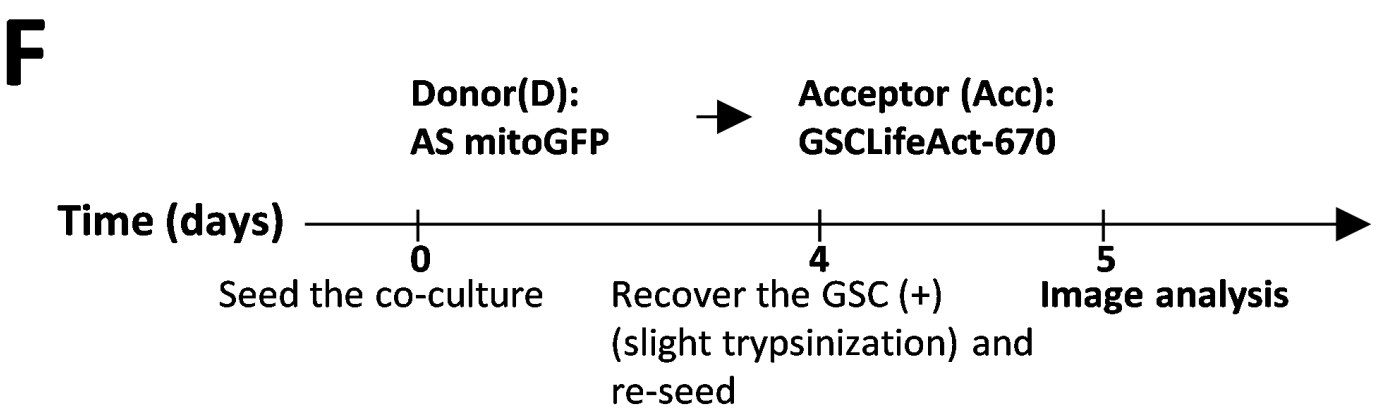

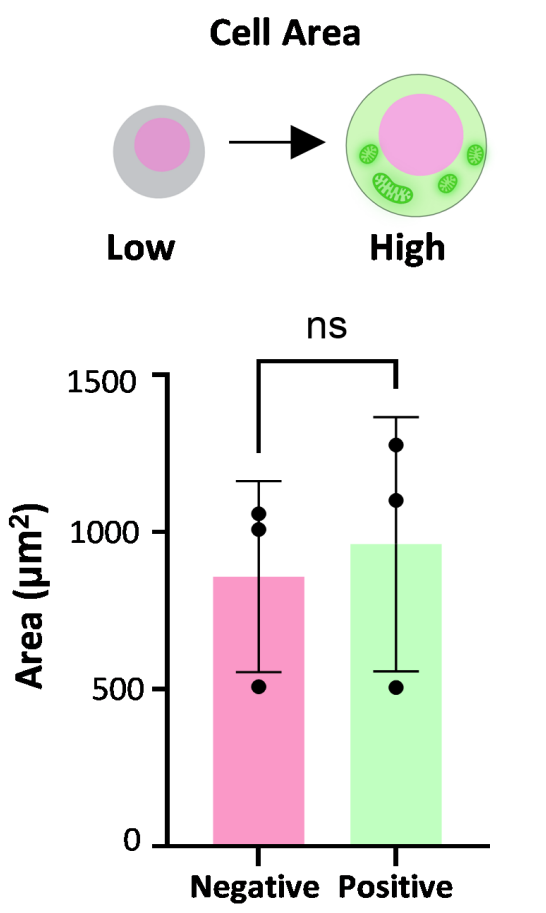

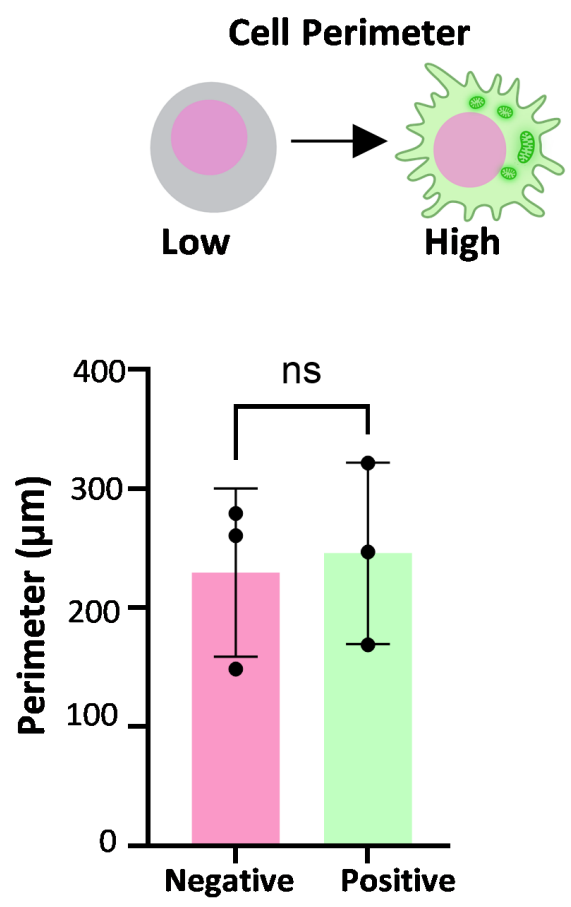

Parameter	Morphological feature
Area	Size
Perimeter	Boundary complexity
Circularity	Global shape
Aspect ratio	Elongation

Area (µm²)	
Negative	858.2 ± 303.9
Positive	961.3 ± 404.1

Perimeter (µm)	
Negative	229.4 ± 70.7
Positive	245.7 ± 76.2

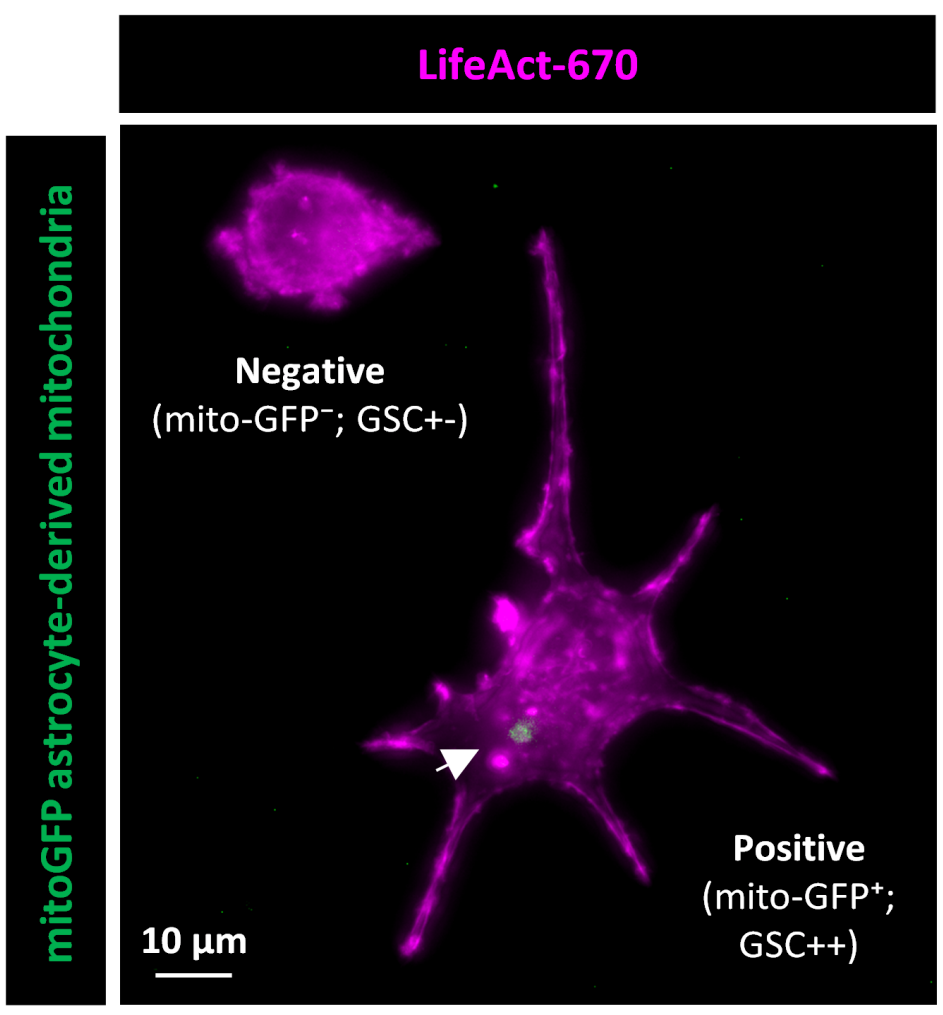

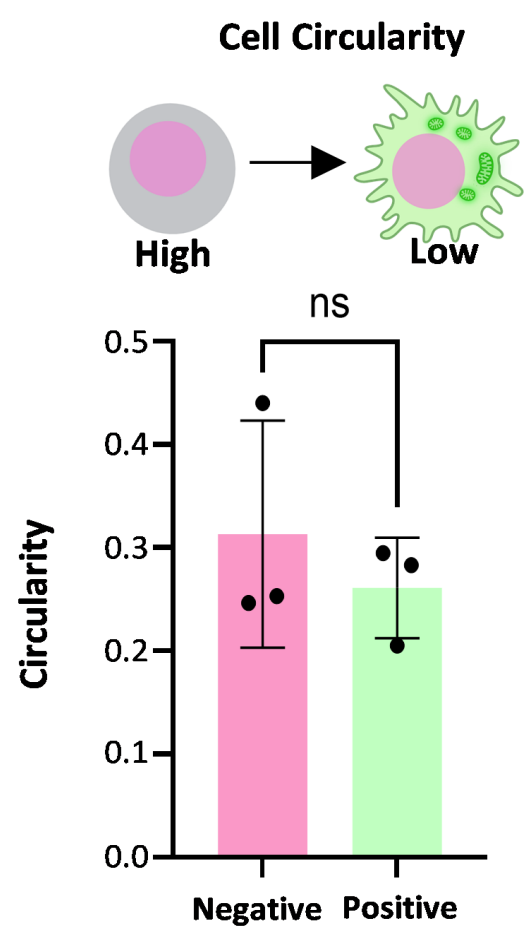

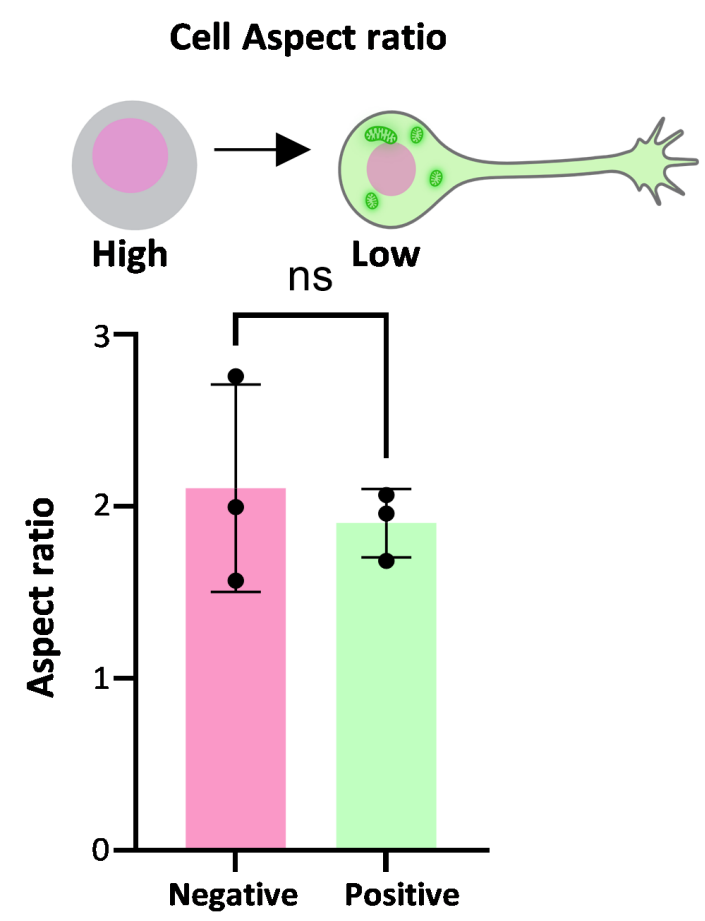

Circularity	
Negative	0.31 ± 0.1
Positive	0.26 ± 0.04

Aspect ratio	
Negative	2.11 ± 0.6
Positive	1.9 ± 0.2

Supplementary Fig. 9 | Increased mitochondrial activity in GSCs receiving astrocyte-derived mitochondria. (A) Experimental design. Primary astrocytes (AS) expressing mitoGFP (donor, D) were co-cultured with H2B-700-expressing GSCs (acceptor, Acc) for 4 days, stained with TMRM, and analyzed by flow cytometry. **(B)** Representative flow cytometry gating strategy distinguishing GSCs receiving astrocyte-derived mitochondria (mitoGFP^{+}; GSC++) from GSCs lacking transferred mitochondria (mitoGFP^{-}; GSC+−). **(C)** Representative overlay histograms of TMRM fluorescence showing a rightward shift in mitoGFP^{+} GSCs. **(D)** Representative density contour plot of cell distribution. **(E)** Quantification of TMRM mean fluorescence intensity (MFI) in mitoGFP^{-} (GSC+−) and mitoGFP^{+} (GSC++) cells. MitoGFP^{+} GSCs exhibited significantly higher TMRM fluorescence than mitoGFP^{-} GSCs (62,589 ± 10,671 vs. 36,105 ± 2,512). Two-tailed Mann–Whitney test; P = 0.0022. *N* = 2 independent experiments, each performed in triplicate. **(F)** Morphological analysis of mitoGFP^{+} and mitoGFP^{-} GSCs revealed no significant differences in cell area, perimeter, circularity, or aspect ratio. Representative LifeAct-670 images of mitoGFP^{-} and mitoGFP^{+} GSCs are shown. Scale bar, 10 µm. Total biological replicates: *N* = 3. Data are presented as the mean ± s.d. Two-tailed Mann–Whitney test. Exact *P* values and summary statistics are provided in the Source Data. Created in BioRender. Saenz-de-Santa-Maria, I. (2026) https://BioRender.com/snix0pc.

Supplementary Fig. 10

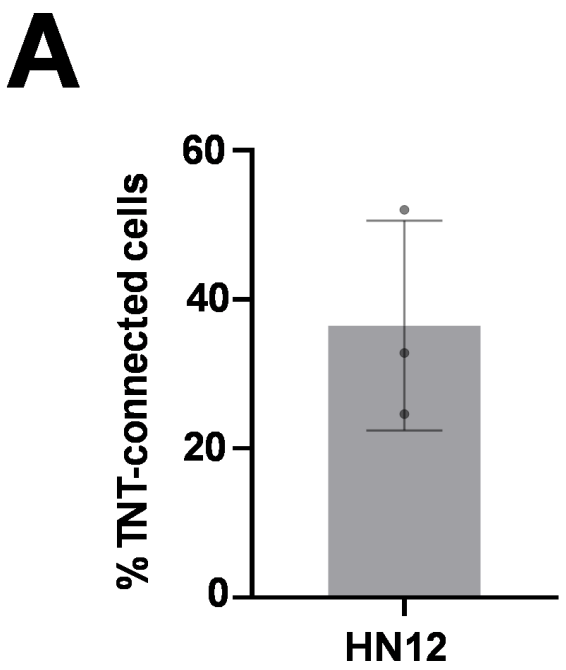

Total cells	681
Mean	36.48
SD	14.07

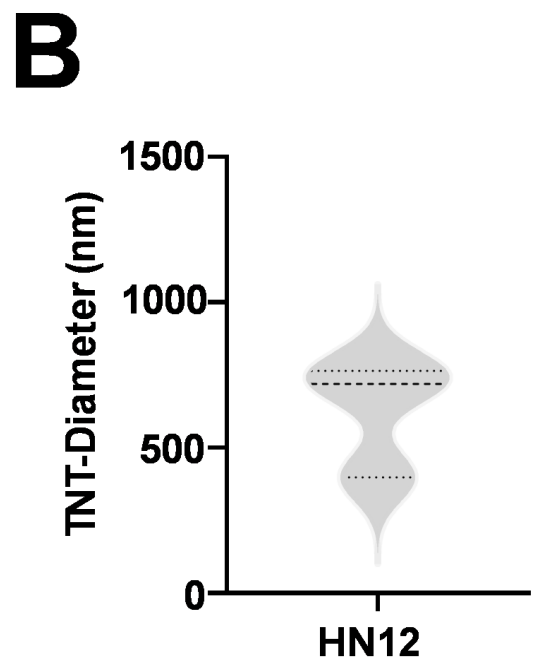

Total TNTs	122
Mean	627.08
SD	199.83

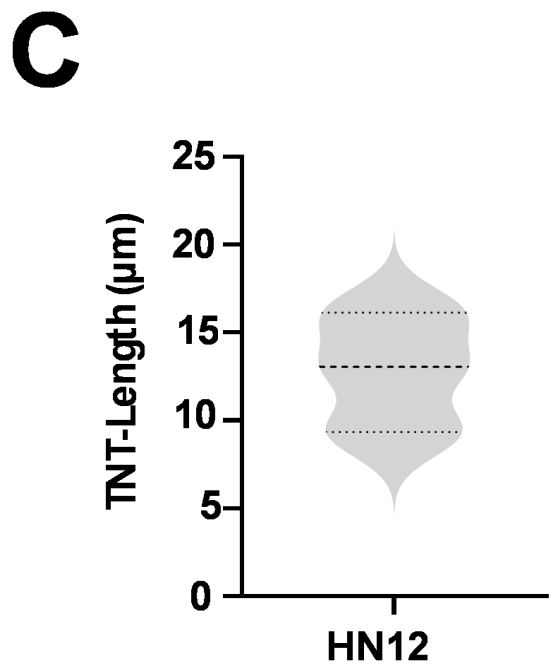

Total TNTs	122
Mean	12.85
SD	3.4

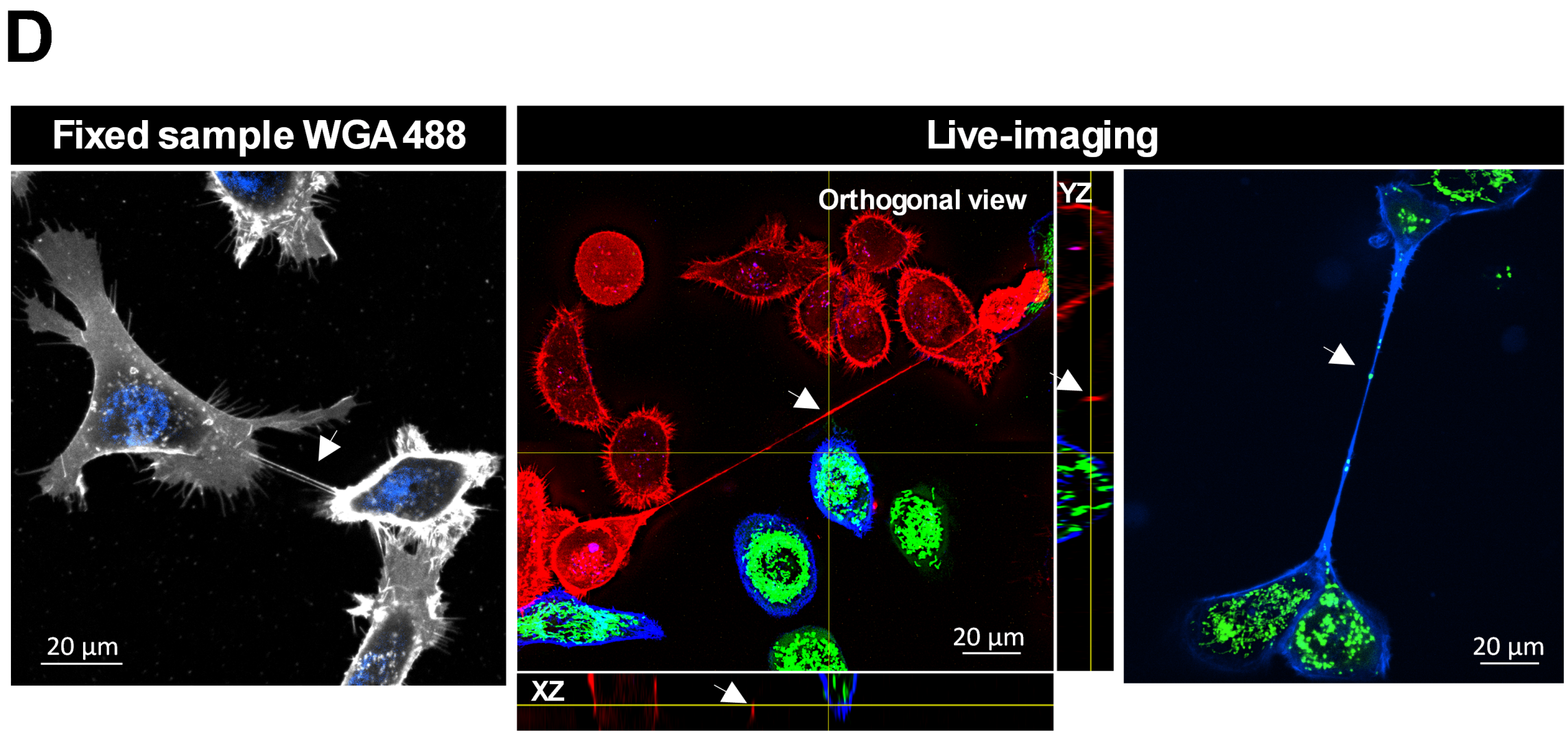

Supplementary Fig. 10 | TNT-like connections in HN12 HNSCC cells display features similar to those of U251 GBM cells *in vitro*. (A–D) Characterization of TNT-like connections in HN12 cells. Quantification of **(A)** TNT-connected cells (36.5 ± 14%), **(B)** TNT apparent diameter (627 ± 200 nm), and **(C)** TNT length (12.8 ± 3.4 µm). A total of 681 cells and 122 TNTs were analyzed across three independent experiments (*N* = 3). **(D)** Representative images of TNTs in HN12 cells. Left, fixed cells labeled with WGA-488. Middle and right, live-cell images showing TNTs connecting neighboring cells. Orthogonal (XZ and YZ) views confirm that the TNTs are suspended above the substrate. White arrowheads indicate TNTs. Scale bars, 20 µm. Representative qualitative observation from three independent experiments (*N* = 3). Summary statistics are provided in the Source Data.

Supplementary Fig. 11

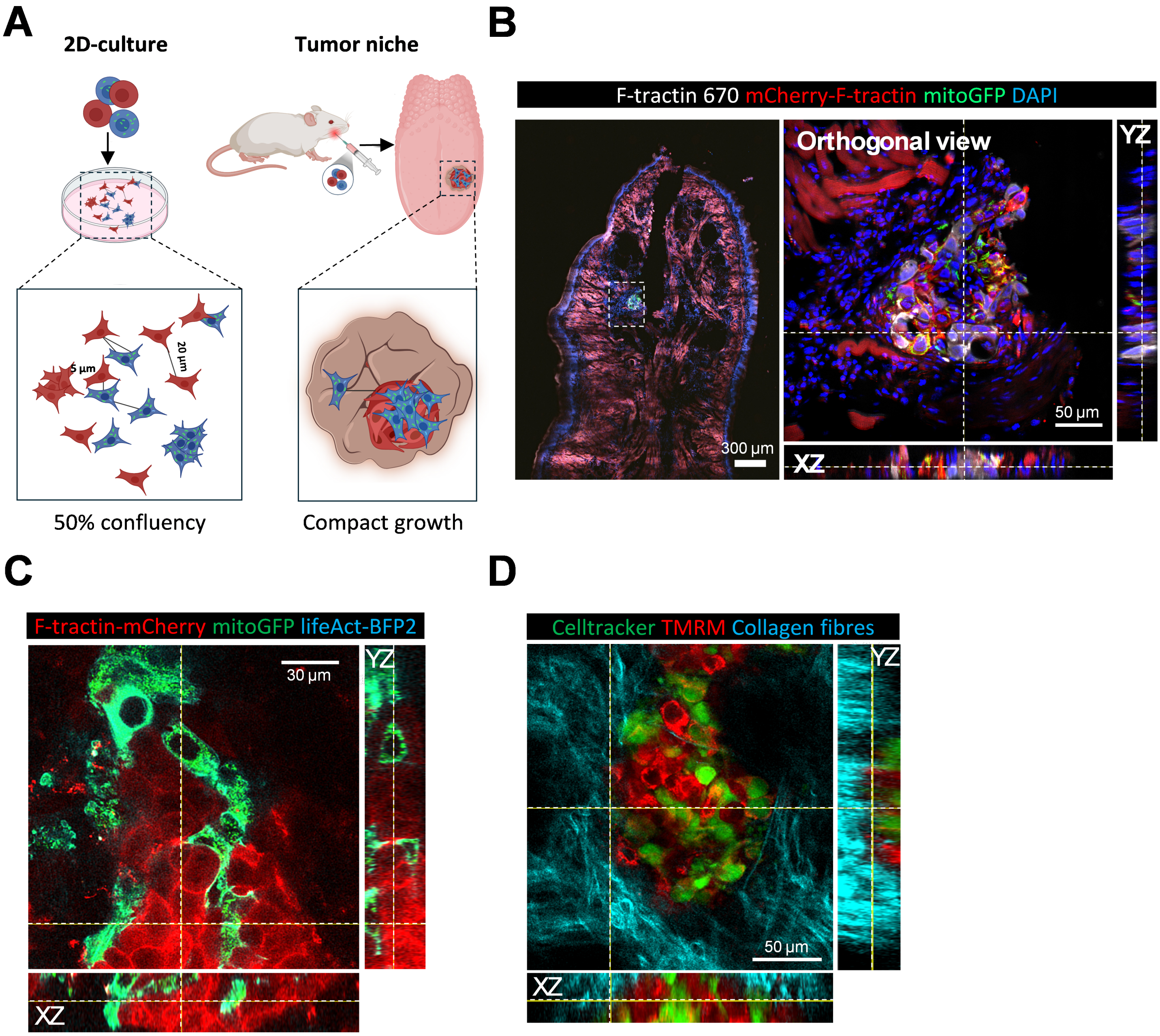

Supplementary Fig. 11 | High cell density within the tumor niche limits TNT visualization *in vivo.* **(A)** Schematic comparing TNT visualization in 2D culture and *in vivo*. A 1:1 mixture of donor (D) and acceptor (Acc) tumor cells was either seeded in culture dishes or injected into the tongues of athymic nu/nu mice. Dispersed cells in 2D culture facilitate TNT visualization (Supplementary Movie 14), whereas tumor growth *in vivo* generates a compact tumor niche that hinders TNT detection. **(B)** Representative image of a fixed tongue section from an orthotopic HN12 xenograft containing donor HN12 mitoGFP-mCherry cells (mitochondria, green; actin, red), acceptor HN12 LifeAct-670 cells (actin, white), and nuclei (blue). Tongues were fixed by cardiac perfusion with Fix1 and Fix2, cryosectioned, and imaged at 20× and 40× magnification. Right, orthogonal view of the compact tumor niche. Representative qualitative observation from three independent experiments (N = 3). **(C, D)** Representative orthogonal ISMic views of the tumor niche *in vivo* show densely packed cells in direct membrane contact, limiting TNT visualization. **(C)** HN12 donor cells expressing mitoGFP and LifeAct-BFP2 (mitochondria, green; actin, light blue) co-cultured with HN12 acceptor cells expressing F-tractin-mCherry (actin, red) (Supplementary Movies 15 and 16). Representative qualitative observation from eight independent experiments (N = 8). **(D)** HN12 cells labeled with CellTracker Green (cytoplasm, green) and TMRM/CellTracker Red (mitochondria and cytoplasm, red) (Supplementary Movie 17). Representative qualitative observation from two independent experiments (N = 2). Scale bars**,** 30 µm (C) and 50 µm (D). *Created in BioRender. Saenz-de-Santa-Maria, I. (2026) https://BioRender.com/8hk6hyd*
