# Supplementary material for: Tunneling nanotubes regulate mitochondrial homeostasis between glioblastoma and astrocytes, and between tumor cells in vivo
Source: Nat Commun. 2026 Sep 15;17:9788. doi: 10.1038/s41467-026-76619-9 (PMC13578765; doi:10.1038/s41467-026-76619-9)
Supplement: Supplementary file 2 [file 41467_2026_76619_MOESM24_ESM.pdf]

nature portfolio

Corresponding author(s): Ines Saenz-de-Santa-Maria; ines.ssantamaria@ciberonc.es
Chiara Zurzolo; chiara.zurzolo@pasteur.fr

Last updated by author(s): Jun 26, 2026

Reporting Summary

Nature Portfolio wishes to improve the reproducibility of the work that we publish. This form provides structure for consistency and transparency in reporting. For further information on Nature Portfolio policies, see our Editorial Policies and the Editorial Policy Checklist.

Statistics

For all statistical analyses, confirm that the following items are present in the figure legend, table legend, main text, or Methods section.

n/a	Confirmed	
☐	☒	The exact sample size (*n*) for each experimental group/condition, given as a discrete number and unit of measurement
☐	☒	A statement on whether measurements were taken from distinct samples or whether the same sample was measured repeatedly
☐	☒	The statistical test(s) used AND whether they are one- or two-sided<br>*Only common tests should be described solely by name; describe more complex techniques in the Methods section.*
☒	☐	A description of all covariates tested
☐	☒	A description of any assumptions or corrections, such as tests of normality and adjustment for multiple comparisons
☐	☒	A full description of the statistical parameters including central tendency (e.g. means) or other basic estimates (e.g. regression coefficient) AND variation (e.g. standard deviation) or associated estimates of uncertainty (e.g. confidence intervals)
☐	☒	For null hypothesis testing, the test statistic (e.g. *F*, *t*, *r*) with confidence intervals, effect sizes, degrees of freedom and *P* value noted<br>*Give P values as exact values whenever suitable.*
☒	☐	For Bayesian analysis, information on the choice of priors and Markov chain Monte Carlo settings
☒	☐	For hierarchical and complex designs, identification of the appropriate level for tests and full reporting of outcomes
☒	☐	Estimates of effect sizes (e.g. Cohen's *d*, Pearson's *r*), indicating how they were calculated

Our web collection on statistics for biologists contains articles on many of the points above.

Software and code

Policy information about availability of computer code

Data collection	Fluorescence image acquisition: Fluorescence images of 2D and 3D cultures were acquired using ZEN Blue software on an LSM confocal microscope or Nikon NIS-Elements software on a Nikon Eclipse Ti2 spinning-disk confocal microscope. Intravital two-photon microscopy in live rodents was performed using an inverted laser-scanning two-photon microscope (MPE-RS; Olympus, Center Valley, PA, USA) equipped with a tunable laser (Insight DS+; Spectra-Physics, Santa Clara, CA, USA).
Data analysis	RNA-seq data were analyzed using the Sequana RNA-seq pipeline (v0.19.1), which is based on the Snakemake workflow management system and is available at https://github.com/sequana/sequana_rnaseq.<br>Image analysis was performed using ICY software with the TNT-connected cells plugin (http://icy.bioimageanalysis.org) or FIJI software. Mitochondrial fusion movies were analyzed in FIJI using the Labkit annotation plugin (https://doi.org/10.3389/fcomp.2022.777728). Co-localization assays were performed using the JACoP plugin in FIJI. Mitochondrial tracking within tunneling nanotubes (TNTs) was performed using the TrackMate extension available for the FIJI platform, installed via the TrackMate-Kymograph update site in the FIJI updater.<br>Flow cytometry (FACS) data were analyzed using FlowJo software (v10.10.1).<br>Intravital microscopy image stacks were deconvolved using Huygens Professional Deconvolution software (Scientific Volume Imaging B.V., Hilversum, The Netherlands). Three-dimensional (3D) volume rendering was performed using Imaris (v9.8.2, 64-bit; Bitplane), and sample drift was corrected using the Correct 3D Drift plugin in FIJI.<br>Statistical analyses and graph preparation were performed using GraphPad Prism (v10.0). Schematic illustrations were created using BioRender.

For manuscripts utilizing custom algorithms or software that are central to the research but not yet described in published literature, software must be made available to editors and reviewers. We strongly encourage code deposition in a community repository (e.g. GitHub). See the Nature Portfolio guidelines for submitting code & software for further information.

Data

Policy information about availability of data

All manuscripts must include a data availability statement. This statement should provide the following information, where applicable:
- Accession codes, unique identifiers, or web links for publicly available datasets
- A description of any restrictions on data availability
- For clinical datasets or third party data, please ensure that the statement adheres to our policy

The data, tools, and material (or their source) that support the article are available from the corresponding author upon reasonable request. The data, materials, and software supporting the findings of this study are described in the main text, figures, Supplementary Information, and the Materials and Methods section. Microscopy image files and biological materials generated during this study are available from the corresponding authors upon reasonable request. The tool to track mitochondria in TNTs is publicly available as a TrackMate extension, in the FIJI software platform. It can be freely installed by subscribing to the TrackMate-Kymograph update site in the FIJI updater. All data supporting the findings of this study are available within the paper, supplementary material, and Source data files. All microscopy image files, and biological materials generated are available from the corresponding authors upon request. The RNA-seq dataset and DE results are deposited in a public repository: https://www.ebi.ac.uk/biostudies/ArrayExpress/studies/E-MTAB-15406?key=3b85fab0-11e9-4852-aa9d-455dbff57878. DOI10.6019/E-MTAB-15406. Source data are provided with this paper.

Research involving human participants, their data, or biological material

Policy information about studies with human participants or human data. See also policy information about sex, gender (identity/presentation), and sexual orientation and race, ethnicity and racism.

Reporting on sex and gender	N/A
Reporting on race, ethnicity, or other socially relevant groupings	N/A
Population characteristics	N/A
Recruitment	N/A
Ethics oversight	N/A

Note that full information on the approval of the study protocol must also be provided in the manuscript.

Field-specific reporting

Please select the one below that is the best fit for your research. If you are not sure, read the appropriate sections before making your selection.

☒ Life sciences ☐ Behavioural & social sciences ☐ Ecological, evolutionary & environmental sciences

For a reference copy of the document with all sections, see nature.com/documents/nr-reporting-summary-flat.pdf

Life sciences study design

All studies must disclose on these points even when the disclosure is negative.

Sample size	No statistical method was used to predetermine sample size. Sample size was determined empirically and was based on previous experience from similar studies. In vitro experiments were performed including at least 3 biological replicates to confirm reproducibility. Sample or group sizes of the experiments were chosen based on previous experience and published studies.
Data exclusions	No data points/biological replicates were excluded from analyses.
Replication	All experiments were repeated at least three times independently, except in rare cases, as specified in the corresponding figure legends.The number of biological replicates (N), technical replicates (n), sample sizes, statistical tests, and exact P values are provided in the corresponding figure legends.
Randomization	N/A
Blinding	Investigators were not blinded during experiments and analyses. There were defined groups and blinding was not necessary

Reporting for specific materials, systems and methods

We require information from authors about some types of materials, experimental systems and methods used in many studies. Here, indicate whether each material, system or method listed is relevant to your study. If you are not sure if a list item applies to your research, read the appropriate section before selecting a response.

Materials & experimental systems

n/a	Involved in the study
☐	☒ Antibodies
☐	☒ Eukaryotic cell lines
☐	☐ Palaeontology and archaeology
☐	☒ Animals and other organisms
☐	☐ Clinical data
☐	☐ Dual use research of concern
☐	☐ Plants

Methods

n/a	Involved in the study
☐	☐ ChIP-seq
☐	☒ Flow cytometry
☐	☐ MRI-based neuroimaging

Antibodies

Antibodies used: Rabbit anti-GFAP antibody (Dako, Cat. No. Z0334) was used for immunofluorescence at a dilution of 1:500. (https://www.citeab.com/antibodies/2452274-z0334-glial-fibrillary-acidic-protein-gfap)

Validation: The anti-body used was commercially available, validated, and cited by various studies.

Eukaryotic cell lines

Policy information about cell lines and Sex and Gender in Research

Cell line source(s): The human glioblastoma cell line U251 (U-251 MG) was purchased from Cytion (CLS Cell Lines Service GmbH, Germany; Cat. No. 300385).
Patient-derived glioblastoma stem cells (GSC⁺ and GSC⁻) were established from a single glioblastoma specimen obtained from a patient enrolled in the clinical trial STEMRI (ClinicalTrials.gov Identifier: NCT01872221) (Lemarié et al., 2023).
Murine primary astrocytes culture were prepared from C57BL/6 wild-type or ROSAmT/mGmT mice from our in-house colony (Institut Pasteur, Paris, France) and used at postnatal days 0 to 3 (P0–P3).

Authentication: U251 cell lines was authenticated by the supplier.
GSC cells were characterized by RT-qPCR analysis of markers associated with different neural cell lineages, including GFAP and CHI3L1 (astrocytic markers), TUBB3 and GAP43 (neuronal markers), and OLIG1, OLIG2, SOX11, and SOX2 (oligodendroglial and stem cell markers). This gene expression profile was maintained throughout serial passaging in culture, indicating preservation of the stem cell properties characteristic of glioblastoma stem cells, as previously described (Pinto et al., 2021).
Primary astrocyte cultures were characterized by GFAP immunostaining (rabbit anti-GFAP, Dako, Cat. No. Z0334) and morphology.

Mycoplasma contamination: Absence of mycoplasma contamination was verified with MycoAlertTM Mycoplasma Detection Kit (Lonza).

Commonly misidentified lines (See ICLAC register): *Name any commonly misidentified cell lines used in the study and provide a rationale for their use.*

Palaeontology and Archaeology

Specimen provenance: *Provide provenance information for specimens and describe permits that were obtained for the work (including the name of the issuing authority, the date of issue, and any identifying information). Permits should encompass collection and, where applicable, export.*

Specimen deposition: *Indicate where the specimens have been deposited to permit free access by other researchers.*

Dating methods: *If new dates are provided, describe how they were obtained (e.g. collection, storage, sample pretreatment and measurement), where they were obtained (i.e. lab name), the calibration program and the protocol for quality assurance OR state that no new dates are provided.*

☐ Tick this box to confirm that the raw and calibrated dates are available in the paper or in Supplementary Information.

Ethics oversight: *Identify the organization(s) that approved or provided guidance on the study protocol, OR state that no ethical approval or guidance was required and explain why not.*

Note that full information on the approval of the study protocol must also be provided in the manuscript.

Animals and other research organisms

Policy information about studies involving animals; ARRIVE guidelines recommended for reporting animal research, and Sex and Gender in Research

Laboratory animals	All animal procedures were approved by the National Cancer Institute Animal Care and Use Committee (NCI-ACUC, NIH) under protocol LCMB-040-2-F and were conducted in accordance with the NIH Guide for the Care and Use of Laboratory Animals. Female athymic nude mice (6–8 weeks old) were obtained from The Jackson Laboratory.Female mitoDendra2 mice (6–8 weeks old), which express Dendra2 in all mitochondria (B6;129S-Gt(ROSA)26Sor^{tm1.1(CAG-COX8A/Dendra2)Dcc}/J; The Jackson Laboratory, stock no. 018397), were used for intravital imaging experiments.
Wild animals	N/A
Reporting on sex	N/A
Field-collected samples	Mice were housed in sterile filter-top cages under a 12 h light/12 h dark cycle at 22 ± 2 °C and 40–60% relative humidity, with food and water provided ad libitum.
Ethics oversight	*Identify the organization(s) that approved or provided guidance on the study protocol, OR state that no ethical approval or guidance was required and explain why not.*

Note that full information on the approval of the study protocol must also be provided in the manuscript.

Clinical data

Policy information about clinical studies
All manuscripts should comply with the ICMJE guidelines for publication of clinical research and a completed CONSORT checklist must be included with all submissions.

Clinical trial registration	N/A
Study protocol	*Note where the full trial protocol can be accessed OR if not available, explain why.*
Data collection	*Describe the settings and locales of data collection, noting the time periods of recruitment and data collection.*
Outcomes	*Describe how you pre-defined primary and secondary outcome measures and how you assessed these measures.*

Dual use research of concern

Policy information about dual use research of concern

Hazards

Could the accidental, deliberate or reckless misuse of agents or technologies generated in the work, or the application of information presented in the manuscript, pose a threat to:

No	Yes	
☒	☐	Public health
☒	☐	National security
☒	☐	Crops and/or livestock
☒	☐	Ecosystems
☒	☐	Any other significant area

Experiments of concern

Does the work involve any of these experiments of concern:

No	Yes	
☒	☐	Demonstrate how to render a vaccine ineffective
☒	☐	Confer resistance to therapeutically useful antibiotics or antiviral agents
☒	☐	Enhance the virulence of a pathogen or render a nonpathogen virulent
☒	☐	Increase transmissibility of a pathogen
☒	☐	Alter the host range of a pathogen
☒	☐	Enable evasion of diagnostic/detection modalities
☒	☐	Enable the weaponization of a biological agent or toxin
☒	☐	Any other potentially harmful combination of experiments and agents

Plants

Seed stocks: N/A

Novel plant genotypes: *Describe the methods by which all novel plant genotypes were produced. This includes those generated by transgenic approaches, gene editing, chemical/radiation-based mutagenesis and hybridization. For transgenic lines, describe the transformation method, the number of independent lines analyzed and the generation upon which experiments were performed. For gene-edited lines, describe the editor used, the endogenous sequence targeted for editing, the targeting guide RNA sequence (if applicable) and how the editor was applied.*

Authentication: *Describe any authentication procedures for each seed stock used or novel genotype generated. Describe any experiments used to assess the effect of a mutation and, where applicable, how potential secondary effects (e.g. second site T-DNA insertions, mosiacism, off-target gene editing) were examined.*

ChIP-seq

Data deposition

☐ Confirm that both raw and final processed data have been deposited in a public database such as GEO.

☐ Confirm that you have deposited or provided access to graph files (e.g. BED files) for the called peaks.

Data access links
May remain private before publication.: *For "Initial submission" or "Revised version" documents, provide reviewer access links. For your "Final submission" document, provide a link to the deposited data.*

Files in database submission: *Provide a list of all files available in the database submission.*

Genome browser session
(e.g. UCSC): *Provide a link to an anonymized genome browser session for "Initial submission" and "Revised version" documents only, to enable peer review. Write "no longer applicable" for "Final submission" documents.*

Methodology

Replicates: *Describe the experimental replicates, specifying number, type and replicate agreement.*

Sequencing depth: *Describe the sequencing depth for each experiment, providing the total number of reads, uniquely mapped reads, length of reads and whether they were paired- or single-end.*

Antibodies: *Describe the antibodies used for the ChIP-seq experiments; as applicable, provide supplier name, catalog number, clone name, and lot number.*

Peak calling parameters: *Specify the command line program and parameters used for read mapping and peak calling, including the ChIP, control and index files used.*

Data quality: *Describe the methods used to ensure data quality in full detail, including how many peaks are at FDR 5% and above 5-fold enrichment.*

Software: *Describe the software used to collect and analyze the ChIP-seq data. For custom code that has been deposited into a community repository, provide accession details.*

Flow Cytometry

Plots

Confirm that:

☒ The axis labels state the marker and fluorochrome used (e.g. CD4-FITC).

☒ The axis scales are clearly visible. Include numbers along axes only for bottom left plot of group (a 'group' is an analysis of identical markers).

☒ All plots are contour plots with outliers or pseudocolor plots.

☒ A numerical value for number of cells or percentage (with statistics) is provided.

Methodology

Sample preparation	Cells were initially grown in either monocultures or cocultures depending on the experiment.For collection, cells were washed with PBS, trypsinized, and re-suspended in complete media before being subjected to flow cytometry-assisted sorting. Or fixed in PFA 4% for FACs analysis.
Instrument	BD FACSymphony S6 sorter and FACS data were acquired on a BD LSR Fortessa flow cytometer
Software	BD FACSDiva. FlowJo v10.1.1
Cell population abundance	Double-positive LifeAct-670/BFP2$^+$ mitoGFP/mito-dsRED$^+$ or TdTomato$^+$ mitoGFP$^+$ tumor cells or astrocytes from co-cultures were isolated by fluorescence-activated cell sorting (FACS). The purity and percentage of the sorted cell populations are indicated in the corresponding plots.
Gating strategy	The gating strategy was established using donor and recipient cells labeled individually with a single fluorophore as single-positive controls.

☒ Tick this box to confirm that a figure exemplifying the gating strategy is provided in the Supplementary Information.

Magnetic resonance imaging

Experimental design

Design type	*Indicate task or resting state; event-related or block design.*
Design specifications	*Specify the number of blocks, trials or experimental units per session and/or subject, and specify the length of each trial or block (if trials are blocked) and interval between trials.*
Behavioral performance measures	*State number and/or type of variables recorded (e.g. correct button press, response time) and what statistics were used to establish that the subjects were performing the task as expected (e.g. mean, range, and/or standard deviation across subjects).*

Acquisition

Imaging type(s)	*Specify: functional, structural, diffusion, perfusion.*
Field strength	*Specify in Tesla*
Sequence & imaging parameters	*Specify the pulse sequence type (gradient echo, spin echo, etc.), imaging type (EPI, spiral, etc.), field of view, matrix size, slice thickness, orientation and TE/TR/flip angle.*
Area of acquisition	*State whether a whole brain scan was used OR define the area of acquisition, describing how the region was determined.*

Diffusion MRI ☐ Used ☐ Not used

Preprocessing

Preprocessing software	*Provide detail on software version and revision number and on specific parameters (model/functions, brain extraction, segmentation, smoothing kernel size, etc.).*
Normalization	*If data were normalized/standardized, describe the approach(es): specify linear or non-linear and define image types used for transformation OR indicate that data were not normalized and explain rationale for lack of normalization.*
Normalization template	*Describe the template used for normalization/transformation, specifying subject space or group standardized space (e.g. original Talairach, MNI305, ICBM152) OR indicate that the data were not normalized.*

Noise and artifact removal

Describe your procedure(s) for artifact and structured noise removal, specifying motion parameters, tissue signals and physiological signals (heart rate, respiration).

Volume censoring

Define your software and/or method and criteria for volume censoring, and state the extent of such censoring.

Statistical modeling & inference

Model type and settings

Specify type (mass univariate, multivariate, RSA, predictive, etc.) and describe essential details of the model at the first and second levels (e.g. fixed, random or mixed effects; drift or auto-correlation).

Effect(s) tested

Define precise effect in terms of the task or stimulus conditions instead of psychological concepts and indicate whether ANOVA or factorial designs were used.

Specify type of analysis: ☐ Whole brain ☐ ROI-based ☐ Both

Statistic type for inference

(See Eklund et al. 2016)

Specify voxel-wise or cluster-wise and report all relevant parameters for cluster-wise methods.

Correction

Describe the type of correction and how it is obtained for multiple comparisons (e.g. FWE, FDR, permutation or Monte Carlo).

Models & analysis

n/a	Involved in the study
☐	☐ Functional and/or effective connectivity
☐	☐ Graph analysis
☐	☐ Multivariate modeling or predictive analysis

Functional and/or effective connectivity

Report the measures of dependence used and the model details (e.g. Pearson correlation, partial correlation, mutual information).

Graph analysis

Report the dependent variable and connectivity measure, specifying weighted graph or binarized graph, subject- or group-level, and the global and/or node summaries used (e.g. clustering coefficient, efficiency, etc.).

Multivariate modeling and predictive analysis

Specify independent variables, features extraction and dimension reduction, model, training and evaluation metrics.
