# Supplementary material for: Tunneling nanotubes regulate mitochondrial homeostasis between glioblastoma and astrocytes, and between tumor cells in vivo
Source: Nat Commun. 2026 Sep 15;17:9788. doi: 10.1038/s41467-026-76619-9 (PMC13578765; doi:10.1038/s41467-026-76619-9)
Supplement: Supplementary file 3 [file 41467_2026_76619_MOESM25_ESM.pdf]

nature portfolio

Peer Review File

Tunneling nanotubes regulate mitochondrial homeostasis between glioblastoma and astrocytes, and between tumor cells in vivo

Corresponding Author: Professor Chiara Zurzolo

This file contains all reviewer reports in order by version, followed by all author rebuttals in order by version.

Version 0:

Reviewer comments:

Reviewer #1

(Remarks to the Author)
The manuscript „Tunneling nanotubes mediate mitochondrial homeostasis in cancer" by Saenz-de-Santa-Maria et al (jointly, Weigert lab and Zurzolo lab) demonstrates the existence of tunneling nanotubes-like connections between tumor cells as well as tumor and non-tumor cells (astrocytes) in a glioblastoma model in mice and explores their functions related to mitochondria exchange in vivo. Using subcellularly resolved intravital microscopy, the authors found that damaged mitochondria stemming from tumor cells travel via TNC to astrocytes and induce mitophagy, whereas healthy mitochondria of astrocytes fuel the bioenergetic metabolism of tumor cells.
The manuscript describes highly relevant work for understanding key mechanisms of glioblastoma pathology related to mitochondrial metabolism and shedding light onto the function of thick TNCs in vivo. However, before recommending the manuscript for publication, I have some concerns regarding the results related to both in vitro (2D/3D) and intravital microscopy that need to be addressed in the frame of revisions. Particularly, there is information missing regarding the performance of the microscopy methods used, needed to validate the full reliability of the results, as detailed in the following.
1) In Fig. 1 the authors refer to TNC dimensions in vitro down to 20 nm. What is reasonable for diffraction-limited microscopy is an accuracy of approx. 200 - 300 nm (diffraction limited) in xy (laterally), but no more than 700-800 nm axially (at the highest NA used, 1.42). Which method has been used to assess dimensions below these values?

2) What is the spatial (lateral and axial) resolution achieved in vivo, in the orthotropic tumor model in the tongue, as how was this measured and validated? This is important for both determining the mitochondrial track length, TNC length and mitochondrial speed (as well as MSD). Although the authors refer to a highly-resolved kind of two-photon microscopy published, the information in the present manuscript should be provided.

3) The state of health of mitochondria – central to characterize which type of mitochondria is transferred from glioblastoma cells to astrocytes and which are transferred in the opposite direction, fulfilling different functions – has been assessed using various methods, all indirect methods – from using tagged GFP to transcriptional methods (RNASeq). To strengthen the results, it would be extremely helpful to additionally assess the state of health of the mitochondria directly, both in vitro and in vivo, as they are transferred. One suggestion is to use NAD(P)H and FAD fluorescence – or even better, their fluorescence lifetimes.

Reviewer #2

(Remarks to the Author)
In this manuscript, the authors provide an important addition to the previously published study of Watson et al (Nature Cancer 2023) which claimed that mitochondria exchange between astrocytes and tumor cells via tumor microtubes is increasing tumor aggressiveness.

This is indeed not likely, since mitochondria cannot pass Cx43 gap junctions, which are always interposed in TMs.

Mitochondria exchange via TNTs is an established cell biology mechanisms, and therefore this study contributes important

information for the field: TNTs (which can co-exist with TMs in glioblastoma cells) are THE road for mitochondria exchange between astrocytes and glioblastoma cells. Moreover, the authors convincingly demonstrate that this exchange happens in both directions.

I have the following recommendations to make this study even stronger:

1. The cell biology effects on mitochondria reception should be studied, e.g. effects on cell proliferation, resilience, apoptosis etc in both glioblastoma cells and astrocytes alike.

2. The strongest gene expression effect of receiving mitochondria from astrocytes is an increase in neuronal genes glioblastoma cells. This raises an exciting possibility: does astrocyte-glioma mito shuttle contribute to the cancer neuroscience biology of glioma? Can the authors provide a little more insight into this topic? In addition, does mitochondria transfer induce phenotypic changes indicative of a more neuronal tumor cell phenotype (longer TMs, more branches, spine-like structures of TMs (TNTs?), etc).

3. Since no mitochondria exchange could be observered in vivo in another tumor model, it is possible that the authors show mitochondria moving quickly in TMs (which is also described: Osswald Nature 2015). The authors should at least discuss this limitation, and/or provide evidence that open, mitochondria-containing TNTs exist in this model.

Reviewer #3

(Remarks to the Author)
Summary and General Assessment:

This manuscript presents a comprehensive investigation into the role of TNTs in mediating mitochondrial exchange between GBM cells and their microenvironment, particularly AS, both in vitro and in vivo. Using advanced live imaging techniques, including ISMic, the authors provide stunning microscopy data and compelling evidence for the existence and functionality of TNTs as conduits for bidirectional mitochondrial transfer. The study offers a very thorough characterization of TNTs, distinguishing between Actin-based and MT-based structures in terms of their dimensions, persistence, and transport dynamics. This section is exceptionally well executed and, by itself, represents a valuable contribution to the field, as from my knowledge no similarly detailed structural characterization has been reported to date.

Functionally, the authors demonstrate that AS transfer healthy mitochondria to GBM cells, enhancing their metabolic gene expression. However, it remains unclear how this occurs, and the authors do not sufficiently address the possibility that the observed transcriptional profile may reflect the selection of cells that are inherently primed to form TNTs, rather than being a direct consequence of mitochon

The inclusion of in vivo imaging in an orthotopic model of HNSCC enhances the physiological relevance of the study. Altogether, the manuscript delivers novel insights into TNT-mediated mitochondrial trafficking and its implications for tumor biology.

Overall, this is a remarkably well-executed and visually compelling study that significantly advances our understanding of TNT biology and mitochondrial exchange in cancer. The authors provide an unprecedented level of structural and functional analysis of TNT subtypes and show that the fate of transferred mitochondria depends on the identity of the recipient cell. The imaging quality is exceptional and biologically informative, and the figures communicate the findings with clarity and precision. However, the mechanistic conclusions drawn from the transcriptomic dataset are still premature and need to be expanded, better characterized, and experimentally validated. While some mechanistic gaps remain this work is an important conceptual and technical contribution.

Specific Comments:

1.The authors claim that transferred mitochondria integrate into the recipient cell’s mitochondrial network; however, the evidence provided is not fully conclusive. While time-lapse imaging suggests physical incorporation, the authors do not present quantitative colocalization metrics such as Manders' coefficients in support of this integration, unlike in Figure 4. Including such quantification would make the claim more robust and consistent with their methodology elsewhere.

2.In the flow cytometry analyses, the authors should include single-color controls to clarify gating and validate transfer specificity.

3.When citing work from other groups, the authors need to name the first author of the study, rather than the group leader, as this is the standard scientific practice for credit attribution in research manuscripts.

4.The authors conclude that AS degrade dysfunctional mitochondria from GBM cells via mitophagy. This is convincingly supported by colocalization with lysosomal markers and absence of membrane potential in the transferred mitochondria. However, it is unclear why similar quantitative methods are not applied in Figure 2, where the authors claim integration of transferred mitochondria into the tumor mitochondrial network. Currently, this is inferred visually from live imaging. Applying Manders' coefficient analysis to these events would reinforce the interpretation and align their methodological rigor across the study.

5.The RNA-seq analysis concludes that transcriptional reprogramming occurs in GBM cells following mitochondrial acquisition from AS, with upregulation of genes involved in mitochondrial function. However, the mechanistic link between mitochondrial uptake and gene expression changes remains speculative and should be supported by functional assays.

6.The authors should also consider the possibility that the observed gene expression profile reflects pre-existing heterogeneity, where a subpopulation of cells is more likely to form TNTs and receive mitochondria. Including a control group of GBM cells cultured alone and sorted by the same parameters would help clarify the findings.

7. The RNA-seq dataset and DE results are not provided in the supplementary materials nor deposited in a public repository and are therefore not accessible for review.

Reviewer #4

(Remarks to the Author)
In my view, this is the "state of the art" contribution that provides compelling evidence (both in vitro and in vivo) for the horizontal TNT-mediated transfer of mitochondria between tumor cells (GB in this case) and non-malignant astrocytes (AS) in the TME. The in vivo component, which represents the innovative and physiologically- relevant confirmation of the existing in vitro studies is the highlight of this report. The studies were performed with exceptional scientific rigor.
This work by far exceeds my expertise in in studies of intercellular communication in cancer. I stand in admiration for the depth, intellectual input, technical detail and thoughtful selection of experimental models. The bidirectional exchange of mitochondria between the tumor and non-tumor cells in the TME is beautifully documented and illustrated in the videos. The superb data presentation of both in vitro and in vivo findings is truly impressive.
I would like to ask the authors for inserting a brief comment about the role of extracellular vesicles (EVs), which were reported to transfer mitochondria, vis a vis TNT-mediated transfer. Would the TNT-mediated transfer operate between the cells closely situated to each other in the TME, while EV-mediated transfer represents systemic, as opposed to local, horizontal transfer?

Version 1:

Reviewer comments:

Reviewer #1

(Remarks to the Author)
The authors fully addressed my questions in the revised version of the manuscript and I now recommend the manuscript for publication.

Reviewer #2

(Remarks to the Author)
The authors have responded well to my suggestions and comments.

Reviewer #3

(Remarks to the Author)
I was initially very positive about this work and its scope, which is remarkably well executed for the imaging part, in vitro and in vivo. The study addresses an important and timely question, and I would like to reiterate that I appreciate both the quality of the imaging and the strong relevance of the work. However, it appears that some aspects of my initial recommendation may have been misunderstood, and I do not fully understand the basis for this interpretation.

A major concern remains the bioinformatic analysis, which, in my view, still represents the weakest aspect of the manuscript and has not been adequately addressed. I do not understand why the authors have chosen to retain this dataset in its current form. The manuscript would likely be strengthened either by removing it altogether or by substantially developing the downstream functional validation. At present, the interpretation lacks depth, and the associated functional validation remains somewhat anecdotal. In particular, reliance on a TMRM assay alone does not adequately support the claims being made, as it provides only a limited measure of mitochondrial membrane potential, without addressing key aspects of mitochondrial/cell function, and does not directly relate to mitochondrial homeostasis as claimed in the title. The circularity index, cell area, etc., are inappropriate for studying the cell response with respect to metabolic fitness. Overall, it remains unclear whether the transfer of mitochondria could also induce a stress response in recipient cells that accounts for the observed readouts. A more in-depth interpretation is therefore required to support the conclusion with additional complementary functional assays to substantiate the proposed claims and test the outcome in the transferred cells over different time points.

Another important point that has not been addressed concerns my previous recommendation to discuss the possible existence of cells with a pre-existing enhanced capacity for mitochondrial transfer. This suggestion was intended to highlight

a general limitation inherent to this type of study, rather than to raise a concern specific to the authors' work. Including such a discussion would improve the balance and transparency of the manuscript and provide a more nuanced interpretation of the findings, which is particularly important in the field of mitochondria transfer.

In this context, I would also like to clarify that I did not suggest performing single-cell transcriptomics as stated by the authors. Rather, I recommended expanding the discussion to consider the possibility that subpopulations of cells may be intrinsically more prone to engage in mitochondrial transfer. Such pre-existing differences could account for the observed downstream effects, which may therefore reflect upstream heterogeneity. This point should be revised accordingly. I agree that single-cell RNAseq would clarify this point.

Regarding the flow cytometry data, the inclusion of a single-cell control is appreciated and strengthens the technical rigor of the analysis. The reported ~2% positive population in the secretion assay remains unconvincing in light of these newly presented data. The identified double-positive population appears too closely aligned with the main population, raising concerns that this very weak signal may be artefactual, potentially related to cell stress known to induce autofluorescence, rather than representing a biologically distinct population of recipient cells. The direct transfer is much more convincing and shows a clear +/+ distinct population acquisition in the set.

Finally, the title does not appear to be fully aligned with the strength of the mechanistic and functional data presented. In its current form, it is too broad relative to the level of mechanistic support provided. I would recommend refining the title to better reflect the most robust and compelling aspect of the study, namely the extensive and impressive characterization of TNT-mediated mitochondrial transfer and their integration into recipient cells, which is remarkably well executed in the manuscript. In my view, this represents the core significance of the work and should be more accurately emphasized.

In conclusion, I recommend either substantially strengthening the functional validation of the bioinformatics analyses or, alternatively, removing this component and addressing it in a separate study.

Reviewer #4

(Remarks to the Author)
Thank you for providing the detailed and helpful responses to the review. In particular, I especially thank the authors for clarifying the relatonship between the EVs and TNTs in the context of intercellular horizontal tarnsfers of cellular materials.

Version 2:

Reviewer comments:

Reviewer #3

(Remarks to the Author)
The authors have addressed my comments. Congrats on this nice work.

Open Access This Peer Review File is licensed under a Creative Commons Attribution 4.0 International License, which

permits use, sharing, adaptation, distribution and reproduction in any medium or format, as long as you give appropriate credit to the original author(s) and the source, provide a link to the Creative Commons license, and indicate if changes were made.
In cases where reviewers are anonymous, credit should be given to 'Anonymous Referee' and the source.
The images or other third party material in this Peer Review File are included in the article's Creative Commons license, unless indicated otherwise in a credit line to the material. If material is not included in the article's Creative Commons license and your intended use is not permitted by statutory regulation or exceeds the permitted use, you will need to obtain permission directly from the copyright holder.
To view a copy of this license, visit https://creativecommons.org/licenses/by/4.0/

Point by point Response

Reviewer #1:

The manuscript “Tunneling nanotubes mediate mitochondrial homeostasis in cancer” by Saenz-de-Santa-Maria et al (jointly, Weigert lab and Zurzolo lab) demonstrates the existence of tunneling nanotubes-like connections between tumor cells as well as tumor and non-tumor cells (astrocytes) in a glioblastoma model in mice and explores their functions related to mitochondria exchange in vivo. Using subcellularly resolved intravital microscopy, the authors found that damaged mitochondria stemming from tumor cells travel via TNT to astrocytes and induce mitophagy, whereas healthy mitochondria of astrocytes fuel the bioenergetic metabolism of tumor cells.

The manuscript describes highly relevant work for understanding key mechanisms of glioblastoma pathology related to mitochondrial metabolism and shedding light onto the function of thick TNTs in vivo. However, before recommending the manuscript for publication, I have some concerns regarding the results related to both in vitro (2D/3D) and intravital microscopy that need to be addressed in the frame of revisions. Particularly, there is information missing regarding the performance of the microscopy methods used, needed to validate the full reliability of the results, as detailed in the following.

1) In Fig. 1 the authors refer to TNT dimensions in vitro down to 20 nm. What is reasonable for diffraction-limited microscopy is an accuracy of approx. 200 - 300 nm (diffraction limited) in xy (laterally), but no more than 700-800 nm axially (at the highest NA used, 1.42). Which method has been used to assess dimensions below these values?

We appreciate the reviewer’s careful attention to the optical resolution limits and the opportunity to clarify this point. The 20–700 nm range cited in the manuscript refers to TNT dimensions previously reported by electron microscopy in fixed samples, as referenced in the text. In our study, TNT measurements were performed using spinning-disk confocal microscopy and restricted to TNTs actively transferring mitochondria. Under diffraction-limited conditions, the smallest TNT we reliably detected exhibited an apparent diameter of ~140 nm, and the shortest TNT transferring mitochondria measured 9.15 μm in length. These values were obtained in the 670-nm fluorescence channel (LifeAct) and represent apparent measurements constrained by optical resolution limits.
As correctly noted, structures below ~200–300 nm laterally and ~700–800 nm axially cannot be resolved with diffraction-limited light microscopy at NA 1.42. We have therefore revised the Results and Methods sections to explicitly state that all TNT dimensions reported from spinning-disk microscopy represent apparent values rather than absolute physical diameters.
To further strengthen structural characterization, we performed cryo-electron microscopy (cryo-EM) analysis of TNT ultrastructure. Cryo-EM revealed that TNTs appearing as single tubes by light microscopy are composed of multiple individual TNTs (iTNTs) with diameters of 60–120 nm, containing actin alone or actin together with microtubules. We

also observed bundles of iTNTs reaching diameters up to ~1 µm, consistent with the thickest TNTs observed by spinning-disk microscopy (Supplementary Fig. 4; Supplementary Movie 9). This ultrastructural analysis provides nanoscale validation of TNT organization beyond diffraction limits.

2) What is the spatial (lateral and axial) resolution achieved *in vivo*, in the orthotropic tumor model in the tongue, as how this measured and validated? This is important for both determining the mitochondrial track length, TNT length and mitochondrial speed (as well as MSD). Although the authors refer to a highly resolved kind of two-photon microscopy published, the information in the present manuscript should be provided.

We thank the reviewer for requesting clarification of *in vivo* spatial resolution parameters. *In vivo* imaging of the orthotopic HNSCC model in the tongue was performed using intravital two-photon microscopy (MPE-RS, Olympus) with a tunable Insight DS+ laser (Spectra Physics). Excitation wavelengths were 740 nm (SHG), 900 nm (mitoGFP), and 1080 nm (mCherry–F-tractin and Texas Red–dextran). Using silicon-oil immersion objectives (30×/1.05 NA and 40×/1.25 NA), theoretical two-photon diffraction-limited resolutions were calculated based on point-spread-function limits. For the 40×/1.25 NA objective, lateral resolution ranged from ~190–277 nm depending on wavelength, with axial resolution between ~425–620 nm. These values are now specified in the Methods section. The minimum apparent TNT thickness measured *in vivo* was 590 ± 110 nm, well above lateral resolution limits, and the maximum TNT length was 27.29 ± 0.5 µm. Thus, structural identification of TNT-like connections *in vivo* was performed within reliable optical constraints. Although visualization *in vivo* is inherently more complex due to tissue density and imaging depth, we were able to quantify TNT length, diameter, and persistence, visualize mitochondria within TNTs, and document both actin-driven protrusion and cell dislodgement mechanisms of formation. Continuous full mitochondrial transfer events were not captured *in vivo*; therefore, MSD and mitochondrial transfer speed analyses were performed exclusively *in vitro*, where imaging conditions allow uninterrupted tracking of mitochondrial dynamics.

Supplementary Fig. 4

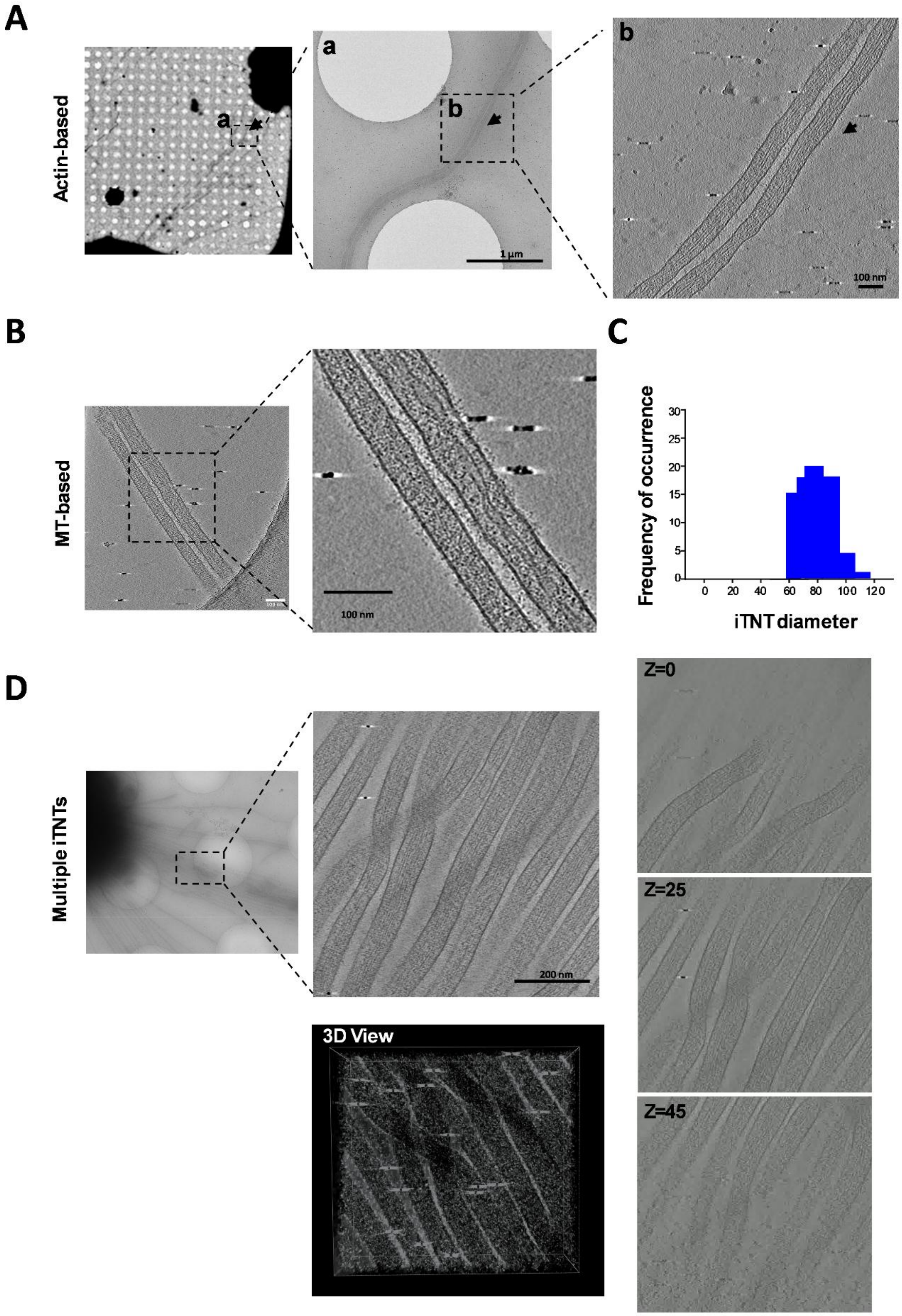

Supplementary Fig. 4 | Ultrastructure of U251 TNT-like connections. (A, B, D) Cryo-EM slices showing TNT-like structures composed of multiple individual TNTs (iTNTs). (A) Actin-based TNT shown at increasing magnification (left, low; a, intermediate; b, high). Boxes indicate the regions magnified. Scale bars: 1 µm (a) and 100 nm (b). (B) High-magnification cryo-EM slice showing a MT-based TNT composed of two iTNTs, one of which contains MT. (C) Quantification of iTNT diameter. (D) Representative TNT bundle composed of up to 14 iTNTs (see Supplementary Movie 9) shown with different Z-stack views and a 3D rendering to better visualize crossing iTNTs. Scale bar: 200 nm

Supplementary Movie 9| Cryo-EM reveals bundles of individual TNTs (iTNTs) in U251 cells. Z-stack and 3D rendering of a cryo-EM tomogram showing bundles of individual TNTs (iTNTs) in U251 cells, illustrating the assembly and intersection of thin iTNTs into thicker TNT-like structures.

---

3) The state of health of mitochondria – central to characterize which type of mitochondria is transferred from glioblastoma cells to astrocytes and which are transferred in the opposite direction, fulfilling different functions – has been assessed using various methods, all indirect methods – from using tagged GFP to transcriptional methods (RNASeq). To strengthen the results, it would be extremely helpful to additionally assess the state of health of the mitochondria directly, both in vitro and in vivo, as they are transferred. One suggestion is to use NAD(P)H and FAD fluorescence – or even better, their fluorescence lifetimes.

Direct metabolic imaging approaches such as NAD(P)H/FAD autofluorescence or FLIM would provide additional resolution; however, we strengthened the functional assessment of mitochondrial status using complementary approaches. First, we measured mitochondrial membrane potential in recipient glioblastoma stem cells (GSCs) using TMRM and flow cytometry (FACS). GSCs that received astrocyte-derived mitochondria (mitoGFP$^+$) exhibited significantly increased TMRM signal compared to non-recipient GSCs within the same co-culture, indicating enhanced mitochondrial membrane potential and functional bioenergetic contribution. Second, tumor-derived mitochondria internalized by astrocytes displayed loss of membrane potential and strong colocalization with lysosomal markers, consistent with mitophagic degradation rather than incorporation. Third, in homotypic U251–U251 transfer experiments, mitoGFP-positive mitochondria co-labeled with MitoTracker™ Red CMXRos were observed traveling through TNTs, indicating preservation of membrane potential during transfer (Supplementary Fig.6 and Movie 13). Together, membrane potential measurements, lysosomal colocalization, transcriptional profiling, and dynamic imaging provide converging functional evidence that mitochondrial transfer is direction-dependent: tumor-derived mitochondria are degraded in astrocytes, whereas astrocyte-derived mitochondria remain functional and enhance mitochondrial activity in tumor cells. While FLIM-based redox analysis would further refine metabolic characterization, the combined structural, dynamic, and functional data support differential mitochondrial fate following TNT-mediated transfer.

Supplementary Fig. 6

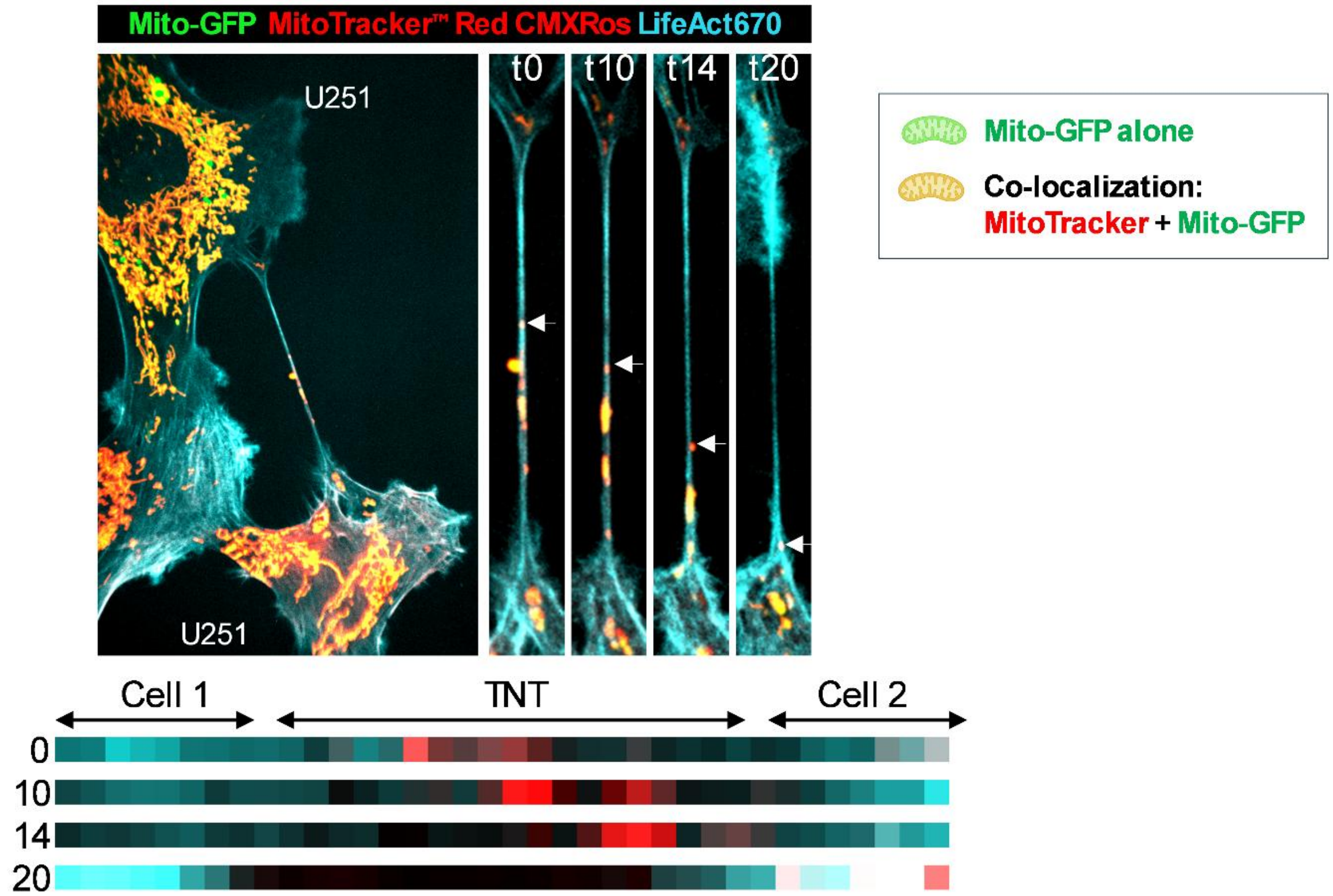

Supplementary Fig.6 | Direct assessment of mitochondrial health during transfer between U251 cells via TNT-like connections. Time-lapse recording of co-staining U251 mito-GFP-lifeAct-670 with MitoTracker™ Red CMXRos, showing yellow mitochondria traveling through TNTs between U251–U251 cells, indicating co-localization of mito-GFP with MitoTracker™ Red and thus the presence of active mitochondria. Left panel: magnified view of the TNT at different time points showing mitochondrial transfer. Below: heatmap illustrating the dynamics of the transfer. (See Supplementary Movie 13).

Supplementary Movie 13 | Mitochondrial health during homotypic U251–U251 transfer via TNTs. MitoGFP-positive mitochondria co-labelled with MitoTracker™ Red CMXRos transfer through TNTs, indicating preserved mitochondrial membrane potential. Total duration, 42 min; acquisition interval, 2 min.

Reviewer #2 (Remarks to the Author):

In this manuscript, the authors provide an important addition to the previously published study of Watson et al (Nature Cancer 2023) which claimed that mitochondria exchange between astrocytes and tumor cells via tumor microtubes is increasing tumor aggressiveness. This is indeed not likely, since mitochondria cannot pass Cx43 gap junctions, which are always interposed in TMs. Mitochondria exchange via TNTs is an established cell biology mechanisms, and therefore this study contributes important information for the field: TNTs (which can co-exist with TMs in glioblastoma cells) are THE road for mitochondria exchange between astrocytes and glioblastoma cells. Moreover, the authors convincingly demonstrate that this exchange happens in both directions.

I have the following recommendations to make this study even stronger:

1) The cell biology effects on mitochondria reception should be studied, e.g. effects on cell proliferation, resilience, apoptosis etc in both glioblastoma cells and astrocytes alike.

We thank the reviewer for this important suggestion. Assessing the biological consequences of mitochondrial transfer is indeed essential to further define its impact on tumor and stromal cells. To directly address this point, we performed functional assessment of mitochondrial activity in recipient glioblastoma stem cells (GSCs). Using flow cytometry, we quantified TMRM fluorescence intensity—a membrane potential–dependent indicator—in GSCs that had received astrocyte-derived mitochondria (mitoGFP^{+}) compared to non-recipient GSCs within the same co-culture. Recipient GSCs exhibited significantly increased TMRM signal, indicating enhanced mitochondrial membrane potential and functional bioenergetic contribution (mean MFI: 62,589.33 ± 10,670.52 vs 36,105.16 ± 2,512.06 in recipient versus non-recipient cells; **new Supplementary Fig. 7**).
In parallel, RNA-seq analysis revealed upregulation of genes associated with mitochondrial function, oxidative phosphorylation, and neuronal-related pathways in recipient cells. To farther investigate the phenotypic consequences, we performed quantitative morphological analysis of recipient and non-recipient GSCs cells. Recipient cells exhibited a significant increase in cell area (mean: 986.0 ± 507.0 vs 776.8 ± 455.2 μm^2 in mito-GFP^{+} vs mito-GFP^{-} cells, respectively) and perimeter (mean: 260.1 ± 121.9 vs 195.4 ± 113.6 μm), accompanied by a significant decrease in circularity (mean 0.26 ± 0.2 vs 0.34 ± 0.2), indicating increased cell size and boundary complexity.
Although aspect ratio shows a non-significant increase the data show a trend towards elongation. Together, these results indicate that mitochondrial acquisition is associated with increased cell size and membrane complexity without significant changes in global cell elongation.
The convergence of increased mitochondrial membrane potential, transcriptional reprogramming, and altered cell morphology supports a functional metabolic and structural consequence of mitochondrial acquisition. Additional phenotypic assays (e.g., proliferation, clonogenicity, and invasion) would further extend these findings. However, we believe that the demonstrated enhancement of mitochondrial activity, together with consistent transcriptional and morphological changes, establishes that mitochondrial transfer is not merely structural but functionally relevant to tumor cell metabolism.

2) The strongest gene expression effect of receiving mitochondria from astrocytes is an increase in neuronal genes glioblastoma cells. This raises an exciting possibility: does astrocyte-glioma mito shuttle contribute to the cancer neuroscience biology of glioma? Can the authors provide a little more insight into this topic? In addition, does mitochondria transfer induce phenotypic changes indicative of a more neuronal tumor cell phenotype (longer TMs, more branches, spine-like structures of TMs (TNTs?), etc).

We appreciate the reviewer's insightful observation regarding the enrichment of neuronal-associated genes following mitochondrial acquisition. In our experimental setting, the

intercellular connections analyzed were consistently thin, shorter, and transient, consistent with canonical TNTs rather than the thicker and longer tumor microtubes (TMs) described in glioma. To explore potential mechanistic overlap, we analyzed GAP43 localization in both homotypic and heterotypic co-cultures. GAP43 was detected within TNT-like structures, suggesting that molecular components associated with TM biology may also contribute to TNT formation or stabilization (Supplementary Fig. Y). As this reviewer points out, the upregulation of neuronal-related genes in recipient tumor cells raises the possibility that astrocyte-derived mitochondrial transfer may support aspects of cancer–neuroscience interactions through metabolic reinforcement of neuronal-like transcriptional programs.

While direct structural remodeling (e.g., altered TNT length or branching) was not assessed in recipient populations, the observed morphological changes—namely increased cell area and perimeter, decreased circularity, and a trend toward increased aspect ratio—together with transcriptional and metabolic changes are consistent with a shift toward enhanced oxidative and neuronal-associated states. We have expanded the Discussion to reflect this conceptual link while maintaining a cautious interpretation.

Supplementary Fig. 7

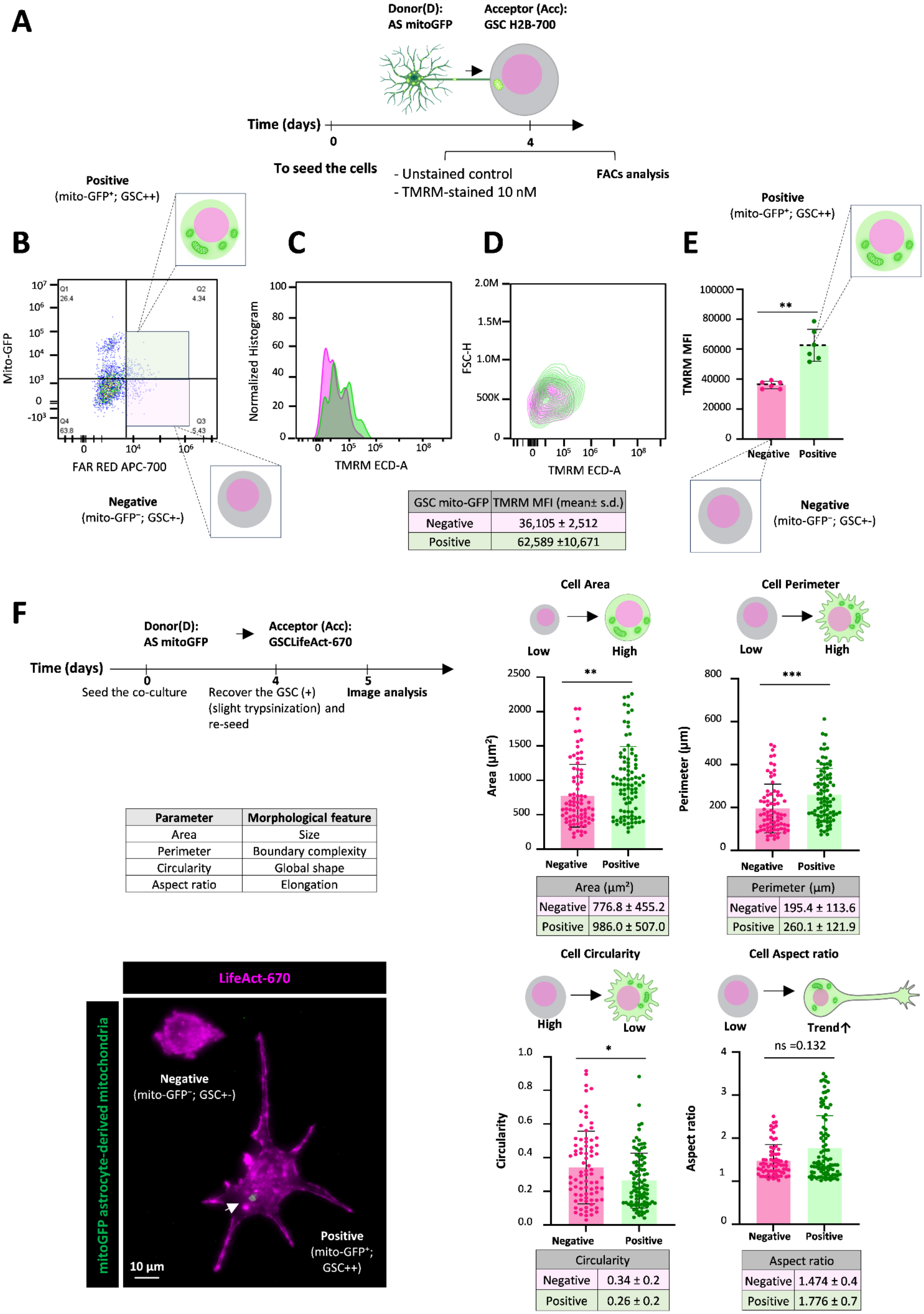

GSC mito-GFP	TMRM MFI (mean± s.d.)
Negative	36,105 ± 2,512
Positive	62,589 ±10,671

Parameter	Morphological feature
Area	Size
Perimeter	Boundary complexity
Circularity	Global shape
Aspect ratio	Elongation

Area (µm²)	
Negative	776.8 ± 455.2
Positive	986.0 ± 507.0

Perimeter (µm)	
Negative	195.4 ± 113.6
Positive	260.1 ± 121.9

Circularity	
Negative	0.34 ± 0.2
Positive	0.26 ± 0.2

Aspect ratio	
Negative	1.474 ± 0.4
Positive	1.776 ± 0.7

Supplementary Fig. 7│ Increased mitochondrial activity and altered cell morphology in GSCs receiving astrocyte-derived mitochondria. (A) Schematic of experimental design. Primary astrocytes expressing mito-GFP (donor) were co-cultured with GSCs expressing H2B-700 (acceptor) for 4 days. Cells were then stained with TMRM and analyzed by FACS. (B) Representative FACS plot showing the gating strategy used to distinguish GSCs that received astrocyte-derived mitochondria (positive, mito-GFP$^+$; GSC++) from non-recipient GSCs (negative, mito-GFP$^-$; GSC+-) within the same co-culture. (C) Overlay histograms of TMRM fluorescence and (D) density contour plot showing a rightward shift in mito-GFP$^+$ GSCs. (E) Quantification of TMRM mean fluorescence intensity (MFI) in mito-GFP$^-$ (GSC+-) and mito-GFP$^+$ (GSC++) GSC populations (mean 36105.16 ± 2512.06 vs 62589.33 ± 10670.52). Data were normally distributed (Shapiro–Wilk test) and analyzed by paired t-test (p**= 0.0010). Data represents 2 independent experiments with triplicates. (F) Morphological analysis of GSCs following mitochondrial transfer. Timeline of the experimental designed. Quantification of cell area, perimeter, circularity, and aspect ratio—reflecting cell size, boundary complexity, global shape, and elongation, respectively—in mito-GFP$^+$ (recipient) and mito-GFP$^-$ (non-recipient) GSCs. Recipient cells exhibit a significant increase in cell area (mean 986.0 ± 507.0 μm², n = 92; 4 outliers excluded using the ROUT method, Q = 1%) compared to non-recipient cells (mean 776.8 ± 455.2 μm², n = 81; Mann–Whitney test, p** = 0.0051). Perimeter is also increased in recipient cells (mean 260.1 ± 121.9 μm, n = 91; 3 outliers excluded) compared to non-recipient cells (mean 195.4 ± 113.6 μm, n = 77; 4 outliers excluded; p*** = 0.0002). Circularity is significantly decreased in recipient cells (mean 0.26 ± 0.2, n = 94) compared to non-recipient cells (mean 0.34 ± 0.2, n = 81; p*= 0.0193), indicating increased boundary complexity. Aspect ratio shows a non-significant difference (mean 1.474 ± 0.4, n = 92; 10 outliers excluded) compared to non-recipient cells (mean 1.776 ± 0.7, n = 71; 2 outliers excluded; p = 0.1317), suggesting a trend toward increased elongation. Data represents three independent experiments. Representative images of LifeAct-670–labeled GSC recipient (mitoGFP$^+$) and non-recipient (mitoGFP$^-$) cells illustrate cytoskeletal morphology. Scale bar: 10 μm.

---

3) Since no mitochondria exchange could be observed *in vivo* in another tumor model, it is possible that the authors show mitochondria moving quickly in TMs (which is also described: Osswald Nature 2015). The authors should at least discuss this limitation, and/or provide evidence that open, mitochondria-containing TNTs exist in this model.

We thank the reviewer for raising this important point regarding the potential distinction between TNTs and tumor microtubes (TMs) *in vivo*. The intercellular structures observed in our model displayed lengths, diameters, and formation mechanisms consistent with TNT-like connections, including actin-driven protrusion and cell dislodgement. In addition, mitochondria were visualized within open-ended connections consistent with TNT-mediated transfer. However, given the high cellular density within the tumor microenvironment, we acknowledge that the coexistence of TNTs and TMs cannot be fully excluded. To address this, we have added clarifying text in the revised manuscript explicitly discussing this possibility and delineating the structural and functional characteristics observed in our model. These additions can be found in the Results and in the Discussion, highlighted in yellow. These revisions clarify the interpretation while maintaining that open-ended TNT-like structures capable of mitochondrial transfer were identified and characterized in our system.

Supplementary Fig. Y

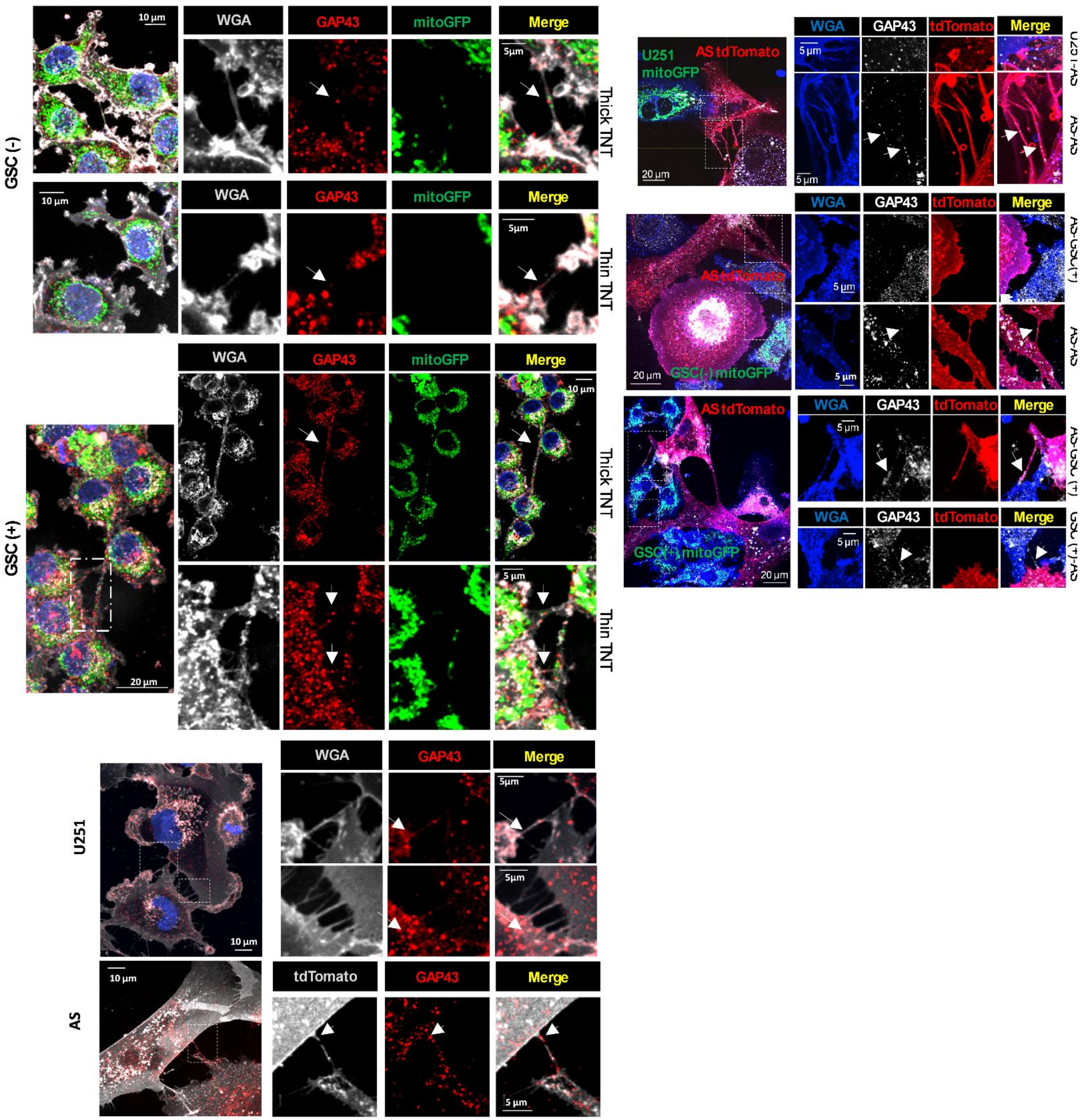

Supplementary Fig. Y│ GAP43 localizes to homotypic and heterotypic TNTs in U251/GSC and AS co-cultures. Representative confocal images showing GAP43 staining in intercellular connections consistent with TNT-like structures. Left: monocultures of GSC (−), GSC (+) expressing mitoGFP, U251, and astrocytes (AS). Right: heterotypic co-cultures of U251–AS and GSC (+)–AS. All conditions were stained for GAP43 (red) and WGA (white). GAP43 signal was detected within both homotypic and heterotypic TNT-like connections, suggesting that GAP43 may contribute to TNT formation and/or stabilization in these settings. Scale bars, 5 μm and 10 μm.

Reviewer #3 (Remarks to the Author):

Summary and General Assessment:

This manuscript presents a comprehensive investigation into the role of TNTs in mediating mitochondrial exchange between GBM cells and their microenvironment, particularly AS, both *in vitro* and *in vivo*. Using advanced live imaging techniques, including ISMic, the authors provide stunning microscopy data and compelling evidence for the existence and functionality of TNTs as conduits for bidirectional mitochondrial transfer. The study offers a very thorough characterization of TNTs, distinguishing between Actin-based and MT-based structures in terms of their dimensions, persistence, and transport dynamics. This section is exceptionally well executed and, by itself, represents a valuable contribution to the field, as from my knowledge no similarly detailed structural characterization has been reported to date.

Functionally, the authors demonstrate that AS transfer healthy mitochondria to GBM cells, enhancing their metabolic gene expression. However, it remains unclear how this occurs, and the authors do not sufficiently address the possibility that the observed transcriptional profile may reflect the selection of cells that are inherently primed to form TNTs, rather than being a direct consequence of mitochondria transfer.

The inclusion of *in vivo* imaging in an orthotopic model of HNSCC enhances the physiological relevance of the study. Altogether, the manuscript delivers novel insights into TNT-mediated mitochondrial trafficking and its implications for tumor biology.

Overall, this is a remarkably well-executed and visually compelling study that significantly advances our understanding of TNT biology and mitochondrial exchange in cancer. The authors provide an unprecedented level of structural and functional analysis of TNT subtypes and show that the fate of transferred mitochondria depends on the identity of the recipient cell. The imaging quality is exceptional and biologically informative, and the figures communicate the findings with clarity and precision. However, the mechanistic conclusions drawn from the transcriptomic dataset are still premature and need to be expanded, better characterized, and experimentally validated. While some mechanistic gaps remain this work is an important conceptual and technical contribution.

1) The authors claim that transferred mitochondria integrate into the recipient cell's mitochondrial network; however, the evidence provided is not fully conclusive. While time-lapse imaging suggests physical incorporation, the authors do not

present quantitative colocalization metrics such as Manders' coefficients in support of this integration, unlike in Figure 4. Including such quantification would make the claim more robust and consistent with their methodology elsewhere.

We appreciate the reviewer's request for quantitative support of mitochondrial integration into the recipient network. To provide clearer dynamic visualization, we performed additional image processing using FIJI with transparent pseudo-color rendering to distinguish donor-derived mitochondria from the recipient network over time. These enhanced time-resolved visualizations are now included as Supplementary Movies 11, 12, and 13, Supplementary Fig. 3 C, and the corresponding description has been added to the Results and Materials and Methods sections. With respect to quantitative colocalization metrics such as Manders' coefficients, these analyses require two independently acquired fluorescence channels representing physically distinct signals. In our experimental design, donor-derived mitochondria are tracked via mito-dsRED, and following transfer, incorporation occurs within the recipient mitochondrial network visualized through the same fluorescence signal. Because donor and recipient mitochondrial populations are not labeled with spectrally separable reporters, computation of colocalization coefficients would require artificial segmentation of a single channel, which would not provide a biologically meaningful metric of integration. A fully quantitative colocalization approach would require donor and recipient cells to express spectrally distinct mitochondrial reporters (e.g., mitoGFP in donors and mitoBFP or mito-dsRED in recipients) to enable unambiguous discrimination of mitochondrial populations. While this represents an important future refinement, the dynamic time-resolved imaging presented here directly demonstrates structural continuity and incorporation behavior over time.

Supplementary Fig. 3

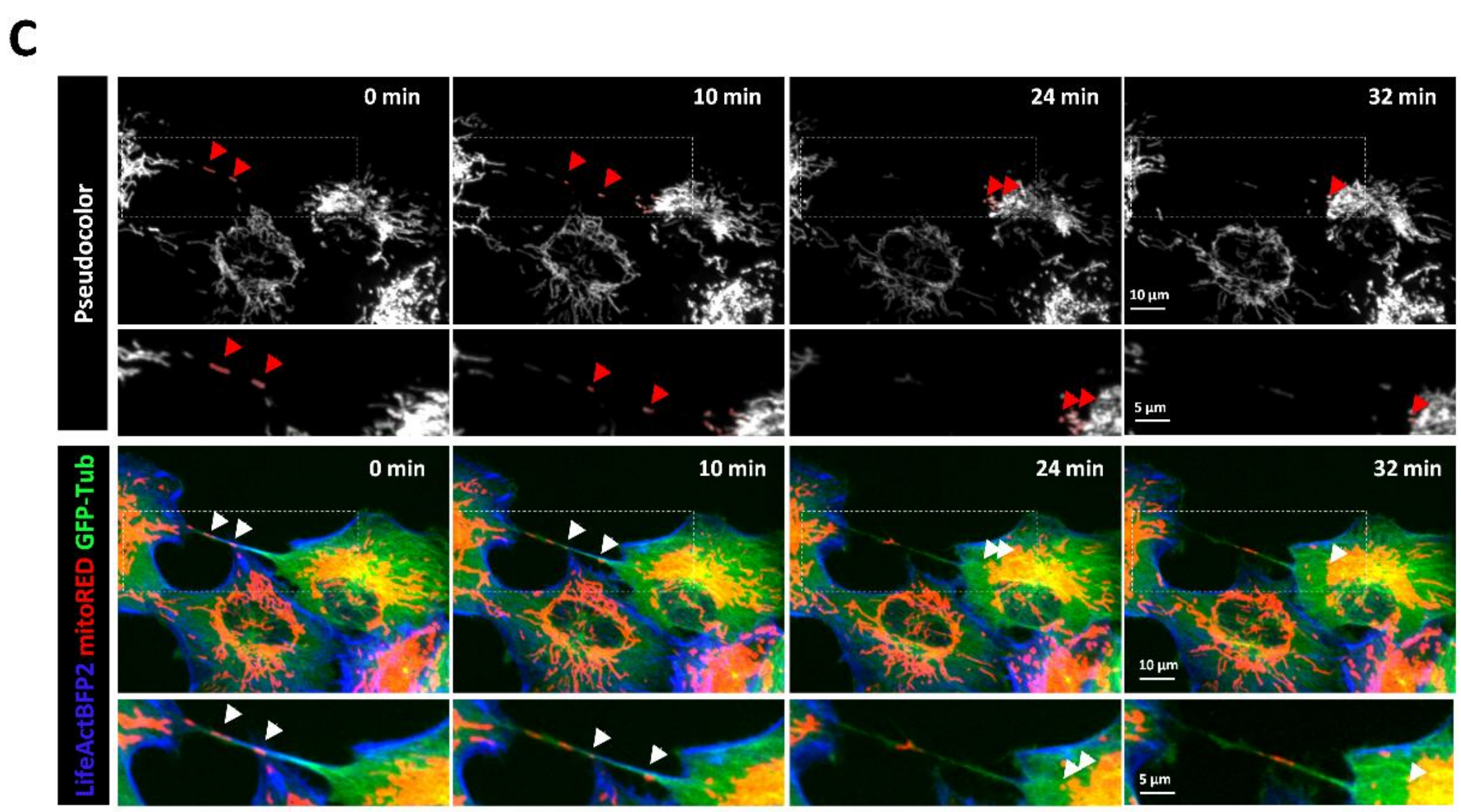

Supplementary Fig. 3 C│ Pseudo-color frames extracted from a time-lapse movie (top) and the corresponding color movie (bottom)**.** Red arrows indicate mitochondria pseudo-color in red traveling through the TNT and fusing with the acceptor cell mitochondria network (See Supplementary Movie 11).

Supplementary Movie 11| Fusion of mitochondria traveling via MT-based TNT into the recipient cell's mitochondrial network. Intermediate movie shown with a transparent pseudo-color overlay to better distinguish donor-derived mitochondria as they integrate into the recipient mitochondrial network. Total duration: 1 hour 16 minutes, with 2-minute intervals.

---

2) In the flow cytometry analyses, the authors should include single-color controls to clarify gating and validate transfer specificity.

We thank the reviewer for highlighting the importance of appropriate gating controls. We have updated Figure 3D to include single-color control samples (donor-only and acceptor-only populations), which were used to define gating boundaries for double-positive cells representing acceptor cells containing transferred mitochondria. The revised figure now explicitly illustrates the gating strategy and validates transfer specificity.

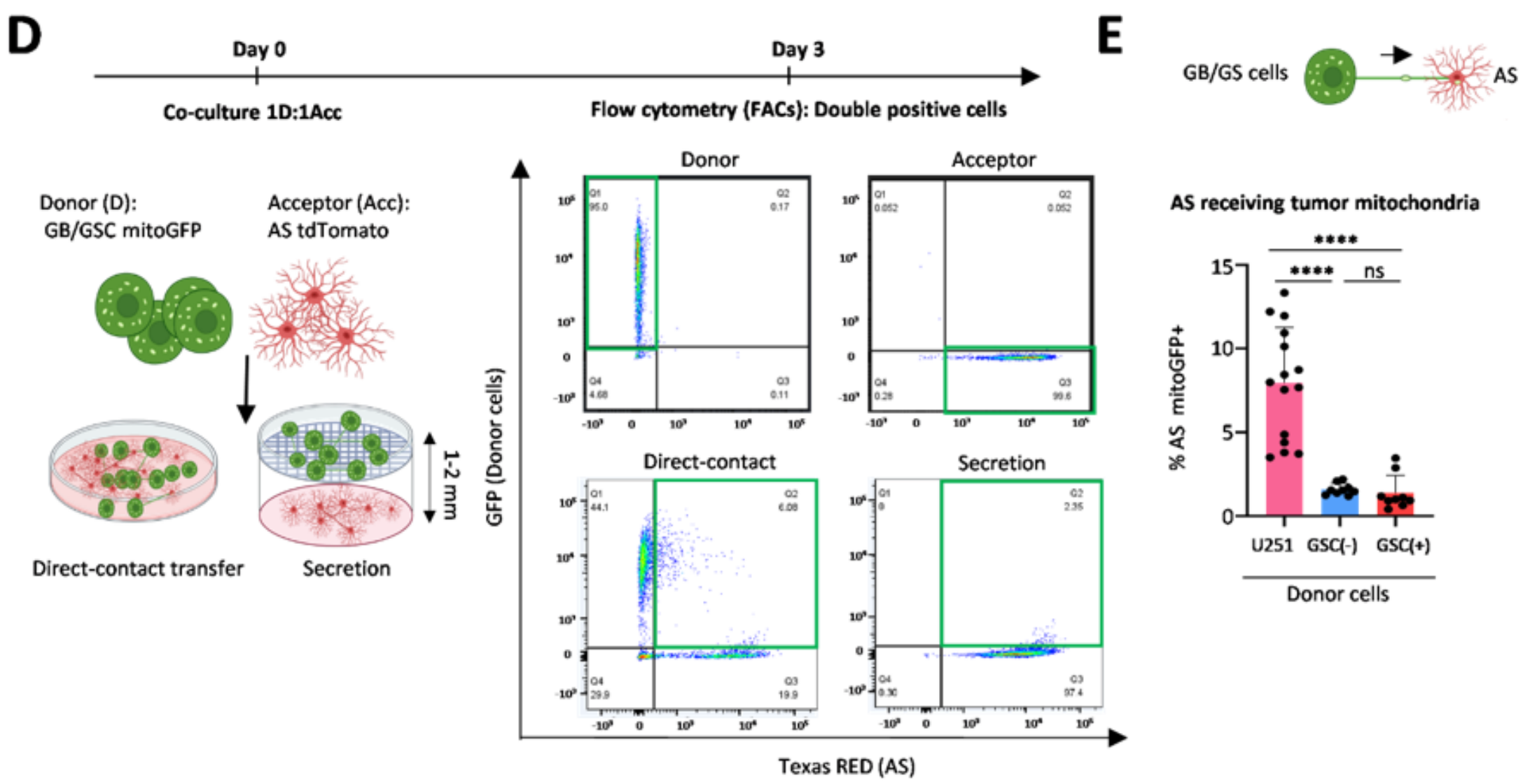

Fig. 3 Pannel D (updated)│ (D) Experimental timeline for analyzing mitochondrial transfer from tumor cells (U251, GSC (-), and GSC (+)) to AS. Experiments were performed as in (B), but with tumor cells and AS as donor (D) and acceptor (Acc) cells, respectively. Representative FACS plots show results for direct contact co-cultures (left) and secretion controls (right), together with single-labeled controls used to define gating. Double-positive events identify Acc containing internalized mitochondria. Acc and D cells are displayed on the X and Y axes, respectively; Acc cells with transferred mitochondria are gated in green. (E) FACS quantification of mitochondrial transfer from tumor cells (D) to AS (Acc). Results show 7.95 ± 3% of AS received mitochondria from U251 cells, significantly more than from GSC (-) (1.6 ± 0.3%) and GSC (+) (1.39 ± 1%). $p < 0.0001$, ns = 0.9712 (two-way ANOVA, multiple comparisons).

---

3) When citing work from other groups, the authors need to name the first author of the study, rather than the group leader, as this is the standard scientific practice for credit attribution in research manuscripts.

We appreciate the reviewer's attention to proper citation practices. The manuscript has been revised accordingly, and references now consistently cite the first author of each

study in accordance with standard scientific convention. All changes are highlighted in the revised version.

4) The authors conclude that AS degrade dysfunctional mitochondria from GBM cells via mitophagy. This is convincingly supported by colocalization with lysosomal markers and absence of membrane potential in the transferred mitochondria. However, it is unclear why similar quantitative methods are not applied in Figure 2, where the authors claim integration of transferred mitochondria into the tumor mitochondrial network. Currently, this is inferred visually from live imaging. Applying Manders' coefficient analysis to these events would reinforce the interpretation and align their methodological rigor across the study.

We thank the reviewer for emphasizing methodological consistency.

Quantitative colocalization analysis was feasible in the mitophagy experiments because transferred mitochondria were evaluated relative to independent lysosomal markers acquired in separate fluorescence channels, allowing objective channel-based colocalization measurements. In contrast, assessment of mitochondrial integration into the recipient tumor network involves redistribution within a continuous mitochondrial compartment lacking spectrally separable donor and recipient markers. Donor-derived mitochondria are tracked via mito-dsRED and, following transfer, become incorporated into a network visualized within the same fluorescence signal. Computation of Manders' coefficients would therefore require artificial segmentation of a single channel and would not provide a biologically meaningful quantitative metric. To further strengthen the evidence, we added enhanced time-resolved imaging (Supplementary Movies 11, 12, and 13, Supplementary Fig. 3 C) demonstrating structural continuity and incorporation dynamics over time. We have clarified this distinction in the revised manuscript (highlighted in yellow).

5) The RNA-seq analysis concludes that transcriptional reprogramming occurs in GBM cells following mitochondrial acquisition from AS, with upregulation of genes involved in mitochondrial function. However, the mechanistic link between mitochondrial uptake and gene expression changes remains speculative and should be supported by functional assays.

We agree that functional validation is essential to support the link between mitochondrial acquisition and transcriptional changes. To address this, we quantified mitochondrial membrane potential in recipient glioblastoma stem cells (GSCs) using TMRM and flow cytometry. GSCs that had received astrocyte-derived mitochondria (mitoGFP$^+$) exhibited significantly increased TMRM fluorescence compared to non-recipient GSCs from the same co-culture. This demonstrates enhanced mitochondrial activity associated with mitochondrial acquisition. The functional increase in membrane potential converges with transcriptional upregulation of oxidative phosphorylation and mitochondrial gene programs, strengthening the mechanistic link between mitochondrial transfer and

metabolic reprogramming. These new data are presented in Supplementary Figure 7 (A-E).

6) The authors should also consider the possibility that the observed gene expression profile reflects pre-existing heterogeneity, where a subpopulation of cells is more likely to form TNTs and receive mitochondria. Including a control group of GBM cells cultured alone and sorted by the same parameters would help clarify the findings.

We appreciate the reviewer's important concern regarding potential pre-existing heterogeneity. Recipient ($mitoGFP^+$) and non-recipient ($mitoGFP^-$) GSC populations were sorted from the same co-culture environment, ensuring identical exposure to astrocytes and shared microenvironmental conditions. This experimental design minimizes confounding baseline differences and strengthens the association between mitochondrial acquisition and transcriptional changes. Importantly, transcriptional reprogramming in recipient cells is accompanied by a measurable functional increase in mitochondrial membrane potential. The convergence of dynamic mitochondrial transfer, enhanced mitochondrial activity, and coordinated gene expression changes supports a functional relationship between mitochondrial acquisition and metabolic reprogramming. While single-cell transcriptomic approaches would provide additional resolution of subpopulation structure, the current design demonstrates a consistent association between mitochondrial acquisition and functional metabolic enhancement within a shared microenvironment.

7) The RNA-seq dataset and DE results are not provided in the supplementary materials nor deposited in a public repository and are therefore not accessible for review.

The complete RNA-seq dataset, including differential expression results, has now been deposited in ArrayExpress under accession number E-MTAB-15406. The accession link has been included in the revised manuscript to ensure full accessibility and transparency.

https://www.ebi.ac.uk/biostudies/ArrayExpress/studies/E-MTAB-15406?key=3b85fab0-11e9-4852-aa9d-455dbff57878

Reviewer #4 (Remarks to the Author):

In my view, this is the "state of the art" contribution that provides compelling evidence (both *in vitro* and *in vivo*) for the horizontal TNT-mediated transfer of mitochondria between tumor cells (GB in this case) and non-malignant astrocytes (AS) in the TME. The *in vivo* component, which represents the innovative and physiologically- relevant confirmation of the existing *in vitro* studies is the highlight of this report. The studies were performed with exceptional scientific rigor. This work by far exceeds my expertise in in studies of intercellular communication in cancer. I stand in admiration for the depth, intellectual input, technical detail and thoughtful selection of experimental models. The

bidirectional exchange of mitochondria between the tumor and non-tumor cells in the TME is beautifully documented and illustrated in the videos. The superb data presentation of both *in vitro* and *in vivo* findings is truly impressive. I would like to ask the authors for inserting a brief comment about the role of extracellular vesicles (EVs), which were reported to transfer mitochondria, vis a vis TNT-mediated transfer. Would the TNT-mediated transfer operate between the cells closely situated to each other in the TME, while EV-mediated transfer represents systemic, as opposed to local, horizontal transfer?

We sincerely thank the reviewer for the generous evaluation of our work and for recognizing both the *in vitro* and *in vivo* components of the study. We appreciate the positive assessment of the structural, dynamic, and physiological relevance of TNT-mediated mitochondrial transfer. In response to the reviewer's question regarding the relationship between extracellular vesicle (EV)-mediated and TNT-mediated mitochondrial transfer, we have expanded the Introduction and the Discussion to explicitly address this distinction (highlighted in yellow). We now clarify that while extracellular vesicles have been reported to mediate mitochondrial transfer, this mechanism likely represents a more diffuse or systemic form of intercellular communication. In contrast, TNT-mediated transfer enables direct, open-ended cytoplasmic continuity between closely apposed cells, allowing targeted and spatially restricted mitochondrial exchange within the tumor microenvironment. These additions highlight that TNT-mediated transfer may operate as a highly localized and regulated mechanism of mitochondrial redistribution, complementing EV-mediated pathways rather than excluding them. We appreciate the opportunity to clarify this conceptual distinction and believe the revised text strengthens the broader context of the study.

Reviewer 3:

I was initially very positive about this work and its scope, which is remarkably well executed for the imaging part, in vitro and in vivo. The study addresses an important and timely question, and I would like to reiterate that I appreciate both the quality of the imaging and the strong relevance of the work. However, it appears that some aspects of my initial recommendation may have been misunderstood, and I do not fully understand the basis for this interpretation.

A major concern remains the bioinformatic analysis, which, in my view, still represents the weakest aspect of the manuscript and has not been adequately addressed. I do not understand why the authors have chosen to retain this dataset in its current form. The manuscript would likely be strengthened either by removing it altogether or by substantially developing the downstream functional validation. At present, the interpretation lacks depth, and the associated functional validation remains somewhat anecdotal. In particular, reliance on a TMRM assay alone does not adequately support the claims being made, as it provides only a limited measure of mitochondrial membrane potential, without addressing key aspects of mitochondrial/cell function, and does not directly relate to mitochondrial homeostasis as claimed in the title. The circularity index, cell area, etc., are inappropriate for studying the cell response with respect to metabolic fitness. Overall, it remains unclear whether the transfer of mitochondria could also induce a stress response in recipient cells that accounts for the observed readouts. A more in-depth interpretation is therefore required to support the conclusion with additional complementary functional assays to substantiate the proposed claims and test the outcome in the transferred cells over different time points.

We thank the reviewer for all their comments that have contributed to enhance the clarity and the message of our work. We agree that the functional validation of the transcriptomic dataset could be further strengthened. However, rather than removing the dataset, we chose to retain it because it provides a valuable framework to interpret the observed mitochondrial transfer in a broader biological context.

While keeping the data, to address the reviewer's concern, we have now:

(i) substantially moderate our interpretation,
(ii) changed the title of the manuscript,
(iii) modified the subheading related to the transcriptomic analysis,
(iv) clearly stated the limitations of the functional validation, and
(v) reframed the transcriptomic analysis as hypothesis-generating rather than definitive.

In particular, we have revised the Abstract, Results and Discussion sections to avoid overinterpreting the data and to better align our conclusions with the level of experimental validation currently provided.

- Abstract lines: 27-28; 32-33; 37
- Results lines: 277-282; 395-396; 407; 420-427
- Discussion lines: 589-590; 596-605; 621-628; 629-630; 679-681

We also acknowledge that TMRM provides a limited readout of mitochondrial membrane potential and does not fully capture mitochondrial function homeostasis. We have therefore revised the manuscript to clarify that these measurements reflect changes in mitochondrial activity rather than a comprehensive assessment of mitochondrial homeostasis (see results lines **420-427**). "Recipient GSCs also exhibited increased mitochondrial membrane potential (TMRM) and morphological changes. These cells showed a significant increase in cell area (986.0 ± 507.0 vs 776.8 ± 455.2 µm² in mitoGFP$^+$ vs mitoGFP$^-$ cells, respectively) and perimeter (260.1 ± 121.9 vs 195.4 ± 113.6 µm), together with reduced circularity (0.26 ± 0.2 vs 0.34 ± 0.2), consistent with increased cell size and boundary complexity. The aspect ratio did not differ significantly (1.474 ± 0.4 vs 1.776 ± 0.7), indicating no substantial change in global cell elongation, although a tendency toward elongation was observed (Supplementary Fig. 7)."

Accordingly, we have tempered our conclusions through the manuscript and adjusted the terminology to better reflect the scope of the data (see discussion lines **621-628**). "Together, our data indicates that GSCs not only eliminate non-functional mitochondria and acquire healthy mitochondria by communicating with neighboring AS but are also stimulated to produce new active mitochondria. This might contribute to increased metabolic activity in cancer cells. However, it is important to note that our assessment of mitochondrial function is based on limited readouts and does not fully capture mitochondrial homeostasis. Further functional analyses will be required to comprehensively define the impact of mitochondrial transfer on recipient cell metabolism".

We have also modified the subheading of the transcriptomic data section accordingly (see result lines **395-396**). "The expression of mitochondrial respiration related genes is enhanced in GSC receiving astrocytic-derived mitochondria"

Finally, we have modified the abstract (see lines **32-33**). "While astrocyte-derived mitochondria to GBM cells correlates with changes in mitochondrial activity and metabolic readouts"

We agree that mitochondrial transfer could, in principle, induce stress responses in recipient cells. We have now included this point in the Discussion and clarified that the observed changes may reflect a combination of adaptative and stress-related responses (see lines **629-630**). "In addition, we cannot exclude that mitochondrial transfer may also induce stress responses in recipient cells, which could contribute to some of the observed changes". Further time-resolved and functional studies of mitochondria functionality will be required to distinguish these possibilities.

Another important point that has not been addressed concerns my previous recommendation to discuss the possible existence of cells with a pre-existing enhanced capacity for mitochondrial transfer. This suggestion was intended to highlight a general limitation inherent to this type of study, rather than to raise a concern specific to the authors' work. Including such a discussion would improve the balance and transparency of the manuscript and provide a more nuanced interpretation of the findings, which is particularly important in the field of mitochondria transfer.

In this context, I would also like to clarify that I did not suggest performing single-cell transcriptomics as stated by the authors. Rather, I recommend expanding the discussion to consider the possibility that subpopulations of cells may be intrinsically more prone to engage in mitochondrial transfer. Such pre-existing differences could account for the observed downstream effects, which may therefore reflect upstream heterogeneity. This point should be revised accordingly. I agree that single-cell RNAseq would clarify this point.

We thank the reviewer for this clarification, and we totally agree with their comments that intrinsic cellular heterogeneity, particularly in glioblastoma, may influence mitochondrial transfer. Indeed, the coexistence of multiple subpopulations with distinct phenotypes could contribute to differential uptake capacities, as well as be a confounding factor.

We have therefore revised the Discussion section accordingly (see lines **596-605**). "Our RNA bulk seq analysis of GSC containing AS-derived mitochondria reveals that transfer of heathy mitochondria (from AS to GSCs) through TNTs is associated with the upregulation of genes related to mitochondrial metabolism (see model in Fig. 8). These findings suggest that the delivery of healthy mitochondria from AS to tumor cells may contribute to increased expression of genes related to energy production and mitochondrial transport, as well as to metabolic and morphological changes consistent with enhanced oxidative states. Nonetheless, while this suggests a potential functional impact of mitochondrial transfer, we cannot exclude the possibility that pre-existing transcriptional heterogeneity in a subset of GSCs contributes to both mitochondrial uptake and the observed gene expression changes".

Regarding the flow cytometry data, the inclusion of a single-cell control is appreciated and strengthens the technical rigor of the analysis. The reported ~2% positive population in the secretion assay remains unconvincing in light of these newly presented data. The identified double-positive population appears too closely aligned with the main population, raising concerns that this very weak signal may be artefactual, potentially related to cell stress known to induce autofluorescence, rather than representing a biologically distinct population of recipient cells. The direct transfer is much more convincing and shows a clear +/+ distinct population acquisition in the set.

We thank the reviewer for this comment. We have therefore revised the Results section to clarify the interpretation of the flow cytometry data (see lines **277-282**). "However, only a minimal double-positive population was detected under secretion conditions, likely reflecting background or artefactual fluorescence rather than effective mitochondrial transfer. In contrast, direct co-culture revealed a clearly distinct double-positive population, supporting TNT-mediated transfer as the predominant mechanism. We have therefore excluded secretion as a major contributor").

Finally, the title does not appear to be fully aligned with the strength of the mechanistic and functional data presented. In its current form, it is too broad relative to the level of mechanistic support provided. I would recommend refining the title to better reflect the most robust and compelling aspect of the study, namely the extensive and impressive characterization of TNT-mediated mitochondrial transfer and their integration into recipient cells, which is remarkably well executed in the manuscript. In my view, this represents the core significance of the work and should be more accurately emphasized.

We agree that the title should better reflect the mechanistic strength and main contribution of the study.

We proposed the following alternative title for publication, and we hope that these changes will satisfy the reviewer:

"Tunneling nanotubes bidirectional transfer of functional mitochondria integration in glioblastoma cells and disposal by astrocytes"

Point by point Response

Third Revision

Reviewer #3 (Remarks to the Author):
The authors have addressed my comments. Congrats on this nice work.
