# Supplementary material for: Tunneling nanotubes regulate mitochondrial homeostasis between glioblastoma and astrocytes, and between tumor cells in vivo
Source: Nat Commun. 2026 Sep 15;17:9788. doi: 10.1038/s41467-026-76619-9 (PMC13578765; doi:10.1038/s41467-026-76619-9)
Supplement: Supplementary file 4 [file 41467_2026_76619_MOESM2_ESM.pdf]

Description of Additional Supplementary Files

Supplementary Movie 1 | Mitochondrial transfer through a TNT between U251 cells expressing mitoDsRED-LifeAct-670 (mitochondria, red; actin, cyan) in 2D culture. Total duration: 2 h 18 min.

Supplementary Movie 2 | Live-cell imaging illustrating TNT apparent diameter measurements over time. Magnified ROI shows a TNT between U251 cells expressing mitoDsRED and LifeAct-670 (mitochondria, red; actin, cyan). The dashed line indicates the measurement region, excluding gondola-like mitochondrial enlargements. Total duration: 32 min; 2-min intervals.

Supplementary Movie 3 | TNT connecting U251 donor cells expressing mitoGFP-LifeAct-BFP2 (mitochondria, green; actin, cyan) and U251 acceptor cells expressing LifeAct-670 (actin, magenta). The TNT persists for up to 7 h. Images were acquired every 2 min. Right, magnified view of the TNT.

Supplementary Movie 4 | TNT connecting U251 donor (D) cells expressing mitoGFP-LifeAct-BFP2 (mitochondria, green; actin, cyan) and acceptor (Acc) cells expressing LifeAct-670 (actin, magenta). The transmitted-light (TL) channel is shown on the right. A mito-GFP+ punctum is visible within the acceptor cell. Total duration: 1 h 10 min; 5-min intervals.

Supplementary Movie 5 | U251 cells co-expressing mito-dsRED, LifeAct-BFP2 and GFP-tubulin (mitochondria, red; actin, blue; microtubules, green) undergoing mitosis in 2D culture. Actin-based TNTs connect dividing and neighboring cells.

Supplementary Movie 6 | Tracking mitochondria traveling through an Actin-based TNT in U251 cells co-expressing mito-dsRED, LifeAct-BFP2 and GFP-tubulin (mitochondria, red; actin, blue; microtubules, green). Right, magnified view of the TNT highlighting mitochondrial tracking and TNT composition. The transmitted-light (TL) channel visualizes cell morphology. Total duration: 24 min; 2-min intervals.

Supplementary Movie 7 | Complete mitochondrial transfer through an Actin-based TNT in GSC (-) cells labelled with MitoTracker Red (mitochondria, red), CellMask Actin 670 (actin, blue), and SPY555-Tubulin (microtubules, green). Total duration: 54 min; 2-min intervals.

Supplementary Movie 8 | Tracking mitochondria traveling through an MT-based TNT in U251 cells co-expressing mito-dsRED, LifeAct-BFP2 and GFP-tubulin (mitochondria, red; actin, blue; microtubules, green). Right, magnified view of the TNT highlighting mitochondrial tracking and TNT composition. Total duration: 1 h 2 min; 2-min intervals.

Supplementary Movie 9 | Cryo-EM tomogram showing bundles of individual TNTs (iTNTs) in U251 cells. Z-stack and 3D rendering illustrate the assembly and intersection of thin iTNTs into thicker TNT-like structures.

Supplementary Movie 10 | Fusion of mitochondria transferred through an Actin-based TNT with the recipient mitochondrial network. Color, pseudo-color and mitochondrial tracking in the red channel are shown. Total duration: 58 min; 2-min intervals

Supplementary Movie 11 | Fusion of mitochondria transferred through a MT-based TNT with the recipient mitochondrial network. A transparent pseudo-color overlay highlights donor-derived mitochondria integrating into the recipient mitochondrial network. Total duration: 1 h 16 min; 2-min intervals.

Supplementary Movie 12 | Fusion of mitochondria transferred through an MT-based TNT with the recipient mitochondrial network. Total duration: 1 h 14 min; 2-min intervals.

Supplementary Movie 13 | Homotypic U251–U251 mitochondrial transfer showing mito-GFP-positive mitochondria co-labelled with MitoTracker™ Red CMXRos traveling through TNTs,

consistent with preservation of mitochondrial membrane potential during transfer. Total duration: 42 min; 2-min intervals.

Supplementary Movie 14 | Imaris 3D rendering of a HN12 F-tractin-mCherry TNT in 2D culture, showing the TNT suspended above the culture surface.

Supplementary Movie 15 | ISMic Z-stack of the tumor niche in an HNSCC xenograft showing HN12 donor cells expressing mito-GFP-LifeAct-BFP2 and HN12 acceptor cells expressing F-tractin-mCherry in direct contact.

Supplementary Movie 16 | Imaris 3D rendering of the tumor niche shown in Supplementary Movie 15, highlighting cell density and compaction. The tumor niche resembles that observed in HNSCC tissue.

Supplementary Movie 17 | ISMic recording of an HN12 tumor niche containing TXR-70-labelled and CellTracker Green-labelled cells, showing the limited migratory behavior of HN12 cells.

Supplementary Movie 18 | Representative ISMic recording showing a TNT connecting HN12 donor cells expressing mitoGFP-LifeAct-BFP2 in the tongue of a live mouse. Left, overview of the tumor niche showing donor cells (mitochondria, green; actin, blue) and acceptor cells expressing F-tractin-mCherry (red). Total duration: 1 h 50 min; 5-min intervals.

Supplementary Movie 19 | TNT formation by the filopodia-growth mechanism in vivo. HN12 cells expressing mitoGFP-LifeAct-BFP2 (mitochondria, green; actin, magenta) form a TNT-like connection within the tumor niche. Left, all channels showing the tumor niche and collagen fibers (light blue). Right, only showing mito-GFP-LifeAct -BFP2 (mitochondria in green, actin filaments magenta) highlighting TNT formation.

Supplementary Movie 20 | TNT formation by the cell dislodgement mechanism in vivo. The tumor niche contains HN12 donor cells expressing mitoGFP-LifeAct-BFP2 (mitochondria, green; actin, cyan) and acceptor cells expressing mCherry-F-tractin (actin, red). Two adjacent cells move apart while maintaining a TNT-like connection. Total duration: 32 min; continuous acquisition.

Supplementary Movie 21 | Imaris 3D rendering of an in vivo TNT-like connection between HN12 donor cells expressing mitoGFP-LifeAct-BFP2 and acceptor cells expressing mCherry-F-tractin.
